# Supplementary material for: Critical time intervention for people leaving prison at risk of homelessness in England and Wales (PHaCT trial): a pilot feasibility randomised controlled trial
Source: BMJ Open. 2025 Dec 16;15(12):e097753. doi: 10.1136/bmjopen-2024-097753 (PMC12719885; doi:10.1136/bmjopen-2024-097753)
Supplement: Supplementary data [file bmjopen-15-12-s002.pdf]

Supplementary Material - Results

Descriptive characteristics for all participants.

	n (%)		
	**Overall sample**	**Intervention**	**Control**
Overall number	**34 (100.0)**	**19 (55.9)**	**15 (44.1)**
Age in years, mean (SD)	38.05 (11.73)	38.68 (13.65)	37.27 (9.14)
Male sex	34 (100.0)	19 (100.0)	15 (100.0)
Ethnicity			
White	31 (91.28)	17 (89.5)	14 (93.3)
Mixed or multiple ethnic group	1 (2.94)	0 (0.0)	1 (6.7)
Caribbean or Black	1 (2.94)	1 (5.3)	0 (0.0)
Any other ethnic group (Bangladeshi)	1 (2.94)	1 (5.3)	0 (0.0)
Smoking status			
Never/Ex-smoker	8 (23.53)	5 (26.3)	3 (20.0)
Current smoker (not every day/every day)	26 (76.47)	14 (73.7)	12 (80.0)
In the 12 months before entering prison did you use illegal drugs?			
No	3 (8.82)	3 (15.8)	0 (0.0)
Yes	31 (91.18)	16 (84.2)	15 (100.0)
Education (could have more than one, selected the highest)			
No formal education	12 (35.29)	5 (26.3)	7 (46.7)
GCSE, GCE or O-level	8 (23.53)	5 (26.3)	3 (20.0)
Vocational training (e.g., Apprenticeship, City & Guilds)	12 (35.29)	9 (47.4)	3 (20.0)
Higher education	1 (2.94)	0 (0.0)	1 (6.7)
Do not know	1 (2.94)	0 (0.0)	1 (6.7)

Employment and benefit receipt before entering prison

Income source of participants before entering prison (part a) and the type of State benefits they received before entering prison.

(a) Source of income before entering prison	N	(b) In receipt of State benefits	N
State benefits only	17	No benefits	6
State benefits & employed	1	Universal credit only	8
State benefits & self employed	1	Universal credit and housing benefits	5
State benefits & proceeds of crime	6	Universal credit, and housing benefits council tax reduction and job seeker's allowance	1
State benefits & help from family/friend	1	Universal credit and sickness or disability	1
State benefits & other (not specified)		Job seeker allowance	1
Employed/self-employed	4	Pension benefit	1
Proceeds of crime	2	Sickness or disability	1
Missing/no response	1		

Note: multiple response allowed

Secondary outcomes results

**Immediately before entering prison, were you in settled accommodation (this is medium to long term accommodation such as social housing or a private rented or owner-occupied home)?**			
No	25 (73.53)	12 (63.2)	13 (86.7)
Yes	9 (26.47)	7 (36.8)	2 (13.3)
If No to the previous question (n=25)			
Hostel	7 (28.00)	3 (25.0)	4 (30.8)
Emergency accommodation (B&B or night shelter)	3 (12.00)	1 (8.3)	2 (15.4)
A caravan or a squat	1 (4.00)	1 (8.3)	0 (0.0)
Temporarily at a friend's or family's house –on an informal basis (sofa surfing)	7 (28.00)	2 (16.7)	5 (38.5)
Rough sleeping, on transport or in transport hub (bus stop or train station), in a tent or car	2 (8.00)	0 (0.0)	2 (15.4)
Other	5 (20.00)	5 (41.7)	0 (0.0)
**Times in life been homeless (Median, IQR)**	3. 0 (2 to 4); n=29	2.0 (1 to 2); n=17	3.5 (3 to 5), n=12
Once	7 (20.59)	6 (31.6)	1 (6.7)
2-9 times	19 (55.88)	10 (52.6)	9 (60.0)
10 or more	3 (8.82)	1 (5.3)	2 (13.3)
Do not know	3 (8.82)	2 (10.5)	1 (6.7)
Missing	2 (5.88)	0 (0.0)	2 (13.3)
**AUDIT-C* 12 months prior prison (Median IQR)**	5.5 (0 to 12)	3 (0 to 12)	9 (0 to 12)
**Visited a GP in prison**			
No	15 (44.12)	9 (47.4)	6 (40.0)
Yes	19 (55.88)	10 (52.6)	9 (60.0)
**Visited a nurse in prison**			
No	10 (29.41)	5 (26.3)	5 (33.3)
Yes	24 (70.59)	14 (73.7)	10 (66.7)
Have you had a hospital admission while in prison			
No	29 (85.29)	15 (78.9)	14 (93.3)
Yes	5 (14.71)	4 (21.1)	1 (6.7)
**Have used any substance use services (e.g. alcohol or drug treatment or support)?**			
No	15 (44.12)	12 (63.2)	3 (20.0)
Yes	19 (55.88)	7 (36.8)	12 (80.0)
Have you had any other healthcare contact / contact with other healthcare worker?			
No	26 (76.47)	12 (63.2)	14 (93.3)
Yes	8 (23.53)	7 (36.8)	1 (6.7)
**Do you currently have any supportive relationships?**			
No supportive relationships	10 (29.41)	8 (42.1)	2 (13.3)
Supportive relationships with friends	2 (5.88)	1 (5.3)	1 (6.7)
Supportive relationships with family	6 (17.65)	2 (10.5)	4 (26.7)
Supportive relationships with family and friends	16 (47.06)	8 (42.1)	8 (53.3)
**ICECAP_A overall score (median, IQR)**	0.672 (0.373	0.698 (0.373	0.589 (0.36

	to 0.888)	to 0.888)	to 0.898)

*AUDIT-C has three questions, each scored from 0 to 4 resulting in a total score on a scale of 0 to 12 (scores of 0 reflect no alcohol use). In men, a score of 4 or more is considered positive. The higher the AUDIT-C score, the more likely it is that the participant's drinking is affecting health and safety.)

Health Economics results

1. Intervention Costs

We liaised with the CTI team, using and adapting their data collection systems to capture the cost of delivering CTI. We captured a broad set of intervention cost components, including: staff costs (i.e. time the CTI staff spends with the participant or communicating with an external agency, such as addiction service), housing costs (i.e. cost of paying rent or purchasing household equipment), travel cost, administrative costs (i.e. time spent by the CTI staff to review and discuss cases) and miscellaneous costs (i.e. any other costs recorded that does not fall under any of the categories above, e.g. costs for clothing).

Table 1 shows the average and total cost of the CTI intervention, by intervention phase. Of the 17 participants randomized to the CTI intervention, 13 completed the pre-engagement phase and phase 1, 9 progressed through phase 2 and only 2 participants completed all the intervention stages. The average cost per participant is £165.5 for the pre-engagement phase only; £194.5 for phase 1, £75.9 for Phase 2, £157.3 for Phase 3. The average cost for those who progress through all the 4 phases of was £674.6.

Table 1 Mean and Median Intervention Cost per Participant by Phases

Phase (n)	**Pre-engagement (17)**	**Phase 1 (13)**	**Pre-engagement+ Phase 1 (13)**	**Phase 2 (9)**	**Pre-engagement to Phase 2 (9)**	**Phase 3 (2)**	**Pre-engagement to Phase 3 (2)**
Mean	£165.5	£194.5	£248.6	£75.9	£355.1	£157.3	£674.6
Median	£74.7	£198.4	£263.7	£37.2	£311.5	£157.3	£674.6

- n= the number of participants who were enrolled in phase

Table 2 shows the total cost of the CTI intervention, by intervention phase and cost category. The cost of staff delivering the intervention represents the main cost component, accounting for 46.3% of total overall costs.

These estimates need to be taken with caution. First, descriptive statistics are based on a very small number of observations, overall and across CTI phases. Delays in recruitment have led to participants not completing all the four phases of the CTI intervention. Descriptive statistics are thus based on a different number of observations, with only 2 participants completing all the CTI intervention phases (from pre-engagement to phase 3). Also, while we initially aimed to collect travel costs as both the actual cost sustained to travel (retrieved from travel claims) and the opportunity costs (i.e. time spent by staff travelling, multiplied by the hourly staff wage), it was not possible to collect the staff opportunity cost of travel to prison. Also, housing costs were only recorded for one participant.

Table 2 Total Intervention Cost by Cost categories and phases

Phases / ***Cost categories***	**Pre-engagement**	**Phase 1**	**Phase 2**	**Phase 3**	**All phases**
Staff Cost	£775.1	£1,585.3	£380.1	£201.3	£2,941.8
% Staff cost	27.5%	62.6%	54.9%	63.9%	46.3%
Travel Cost	£96.8	£147.6	£9.2	£17.8	£271.4

%Travel Cost	3.4%	5.8%	1.3%	5.7%	4.3%
Miscellaneous Cost	£3.6	£87.5	£0.0	£10.0	£101.1
% Miscellaneous	0.1%	3.5%	0.0%	3.2%	1.6%
Housing Cost	£1,467.9	£0.0	£0.0	£0.0	£1,467.9
%Housing Cost	52.1%	0.0%	0.0%	0.0%	23.1%
Review/Admin Cost	£475.8	£710.2	£302.7	£85.5	£1,574.2
% Review Admin Cost	16.9%	28.1%	43.7%	27.2%	24.8%
Total Cost	£2,819.2	£2,530.5	£692.1	£314.6	£6,356.4

2. EQ-5D and ICECAP-A descriptive statistics

Table 3 shows average EQ-5D-3L and ICECAP-A scores at baseline for the 34 participants. The average EQ-5D-3L score of participants at baseline is 0.57 (SD=0.42), which is lower than the population norm[1]. The average ICECAP-A score of participants at baseline is 0.62 (SD=0.29); this is similar to a previous study, considering the population[2].

Table 4 summarizes EQ-5D and ICECAP-A scores by homelessness status and use of drugs and tobacco before entering prison. As expected, drug and tobacco users have a lower quality of life and capability score than non-users, while the association between living in a settled accommodation before entering prison and wellbeing and capability score seems to go in the opposite direction.

Table 3

**EQ-5D-3L and ICECAP-A, average value at baseline**			
	N	Mean	SD
**EQ-5D-3L index**	34	**0.577**	0.420
**ICECAP-A index**	34	**0.615**	0.285

Table 4

**EQ-5D index and ICECAP-A index by key categories**						
**Lived in settled accommodation before entering prison**						
	**Yes**			**No**		
	N	Mean	SD	N	Mean	SD
**EQ-5D index**	9	**0.492**	0.484	25	**0.608**	0.401
**ICECAP-A index**	9	**0.597**	0.374	25	**0.621**	0.254
**Drug and tobacco use before entering prison**						
	**Yes**			**No**		
	N	Mean	SD	N	Mean	SD
**EQ-5D index**	31	**0.565**	0.434	3	**0.703**	0.251
**ICECAP-A index**	31	**0.603**	0.287	3	**0.733**	0.279

When considering EQ-5D-3L single dimension, we found that the percentage of participants reporting severe or moderate problems is lower to the general population across all dimensions. This is especially true for anxiety and depression, where 47% and 27% of participants report experiencing moderate and severe depression and anxiety (vs. respectively, 19% and 1.8% in the general population).

3. Resource use at baseline

Table 5 shows descriptive statistics on healthcare resource use in prison (visits to the GP, nurse, substance use services and hospital admission).

Table 5

**Visited GP in prison**			
	N	%	**Number of visits (mean, SD)**
Yes	19	55.88	4.9 (8.1)
No	15	44.12	
**Visited nurse in prison**			
	N	%	**Number of visits (mean, SD)**
Yes	24	70.59	10.2 (20.1)
No	10	29.41	
**Hospital admission**			
	N	%	**Number of hospital admissions (mean, SD)**
Yes	5	14.71	1.6 (0.89)
No	29	85.29	
**Substance use services**			
	N	%	**Number of visits (mean, SD)**
Yes	19	55.88	2.2 (26)
No	15	44.12	
**Other**			
	N	%	**Number of visits (mean, SD)**
Yes	8	23.53	2.8 (3.2)
No	26	76.47	

References

1. Kind P, Hardman G, Macran S. *UK population norms for EQ-5D*. York: Centre for Health Economics, University of York; 1999.

2. Hunter RM, Anderson R, Kirkpatrick T, Lennox C, Warren F, Taylor RS, et al. Economic evaluation of a complex intervention (Engager) for prisoners with common mental health problems, near to and after release: a cost-utility and cost-consequences analysis. *Eur J Health Econ*. 2022;23(2):193–210. doi: 10.1007/s10198-021-01360-7
