# Supplementary material for: Critical time intervention for people leaving prison at risk of homelessness in England and Wales (PHaCT trial): a pilot feasibility randomised controlled trial
Source: BMJ Open. 2025 Dec 16;15(12):e097753. doi: 10.1136/bmjopen-2024-097753 (PMC12719885; doi:10.1136/bmjopen-2024-097753)
Supplement: Author's manuscript [file bmjopen-2024-097753.draft_revisions.pdf]

BMJ Open

BMJ Open is committed to open peer review. As part of this commitment we make the peer review history of every article we publish publicly available.

When an article is published we post the peer reviewers' comments and the authors' responses online. We also post the versions of the paper that were used during peer review. These are the versions that the peer review comments apply to.

The versions of the paper that follow are the versions that were submitted during the peer review process. They are not the versions of record or the final published versions. They should not be cited or distributed as the published version of this manuscript.

BMJ Open is an open access journal and the full, final, typeset and author-corrected version of record of the manuscript is available on our site with no access controls, subscription charges or pay-per-view fees (http://bmjopen.bmj.com).

If you have any questions on BMJ Open's open peer review process please email info.bmjopen@bmj.com

BMJ Open

Preventing Homelessness, improving health for people leaving prison: a pilot randomised controlled trial of a Critical Time Intervention

Journal:	*BMJ Open*
Manuscript ID	bmjopen-2024-097753
Article Type:	Original research
Date Submitted by the Author:	09-Dec-2024

Complete List of Authors:	Williams, Adam; Cardiff University<br>Jacob, Nina; Cardiff University, Centre for Trials Research<br>Grozeva, Detelina; Cardiff University<br>Lloyd, Barry; Cardiff University<br>Moriarty, Yvonne; Cardiff University, Centre for Trials Research<br>Deidda, Manuela; University of Glasgow, Health Economics and Health Technology assessment<br>Achiaw, Samuel Owusu; University of Glasgow<br>Thomas, Ian; Cardiff University<br>Lewis, Kelly; Cardiff University<br>Cannings-John, Rebecca; Cardiff University, Centre for Trials Research<br>Madoc-jones, Iolo; Wrexham University<br>Fitzpatrick, Suzanne; Heriot-Watt University<br>Katikireddi, Srinivasa; University of Glasgow, MRC/CSO Social & Public Health Sciences Unit<br>Mackie, Peter; Cardiff University, School of Geography and Planning<br>White, James; Cardiff University, Centre for Trials Research<br>Lewsey, Jim; University of Glasgow

Keywords:	Vulnerable Populations, Randomized Controlled Trial, Quality of Life, Prisons, Feasibility Studies

SCHOLARONE™
Manuscripts

I, the Submitting Author has the right to grant and does grant on behalf of all authors of the Work (as defined in the below author licence), an exclusive licence and/or a non-exclusive licence for contributions from authors who are: i) UK Crown employees; ii) where BMJ has agreed a CC-BY licence shall apply, and/or iii) in accordance with the terms applicable for US Federal Government officers or employees acting as part of their official duties; on a worldwide, perpetual, irrevocable, royalty-free basis to BMJ Publishing Group Ltd ("BMJ") its licensees and where the relevant Journal is co-owned by BMJ to the co-owners of the Journal, to publish the Work in this journal and any other BMJ products and to exploit all rights, as set out in our licence.

The Submitting Author accepts and understands that any supply made under these terms is made by BMJ to the Submitting Author unless you are acting as an employee on behalf of your employer or a postgraduate student of an affiliated institution which is paying any applicable article publishing charge ("APC") for Open Access articles. Where the Submitting Author wishes to make the Work available on an Open Access basis (and intends to pay the relevant APC), the terms of reuse of such Open Access shall be governed by a Creative Commons licence – details of these licences and which Creative Commons licence will apply to this Work are set out in our licence referred to above.

Other than as permitted in any relevant BMJ Author's Self Archiving Policies, I confirm this Work has not been accepted for publication elsewhere, is not being considered for publication elsewhere and does not duplicate material already published. I confirm all authors consent to publication of this Work and authorise the granting of this licence.

For peer review only

DRAFT V.04 26.11.24

Preventing Homelessness, improving health for people leaving prison: a pilot randomised controlled trial of a Critical Time Intervention

Short Title: PHaCT: a pilot RCT

Authors:
Adam DN Williams 1* ORCID ID: 0000-0002-4825-8997
Nina Jacobs 1
Detelina Grozeva 1 ORCID ID: 0000-0003-3239-8415
Barry Lloyd 1 ORCID ID: 0000-0003-4763-1726
Yvonne Moriarty 1 ORCID ID: 0000-0002-7608-4699
Manuela Deidda 2 ORCID ID: 0000-0002-0921-6970
Samuel Owusu Achiaw 2 ORCID ID: 0009-0000-5711-7240
Ian Thomas 3 ORCID ID: 0000-0001-9808-0202
Kelly Lewis 1
Rebecca Cannings-John 1 ORCID ID: 0000-0001-5235-6517
Iolo Madoc-jones 4 ORCID ID: 0000-0002-3501-9639
Suzanne Fitzpatrick 5
Srinivasa Vittal Katikireddi 6 ORCID ID: 0000-0001-6593-9092
Peter Mackie 7, ORCID ID: 0000-0003-1494-0864
James White 1,8 ORCID ID: 0000-0001-8371-8453
Jim Lewsey 2 ORCID ID: 0000-0002-3811-8165

Affiliations:
1 – Centre for Trials Research, School of Medicine, College of Biomedical & Life Sciences, Cardiff University, Wales, UK (United Kingdom).
2 – Health Economics and Health Technology Assessment, School of Health and Wellbeing, University of Glasgow, Glasgow, UK
3 – Administrative Data Research Wales/ Wales Institute of Social and Economic Research and Data, School of Social Sciences, Cardiff University, Wales, UK (United Kingdom).
4 – Cyfiawnder The Social Inclusion Research Centre, Wrexham University, Wales, UK
5 - Institute for Social Policy, Housing and Equalities Research (I-SPHERE), Heriot-Watt University
6 - MRC/CSO Social & Public Health Sciences Unit, School of Health and Wellbeing, University of Glasgow, Glasgow, UK
7 - School of Geography and Planning, Cardiff University, UK
8 - DECIPHer, School of Social Sciences, Cardiff University, UK

Corresponding author: *Adam D N Williams PhD. Centre for Trials Research, School of Medicine, Cardiff University, Wales, UK. E-mail: WilliamsAD7@cardiff.ac.uk.

For peer review only - http://bmjopen.bmj.com/site/about/guidelines.xhtml

Abstract

Objective To determine whether a full-scale RCT assessing the efficacy and cost-effectiveness of a housing-led Critical Time Intervention (CTI) is feasible and acceptable.

Design Pilot parallel two-arm individual-level RCT, including process evaluation and embedded exploratory health economic evaluation.

Setting Four male prisons across England and Wales, UK.

Participants Men leaving prison at risk of homelessness and intervention delivery staff.

Intervention CTI has four components: (1) Pre-engagement phase: assessing client's needs and implementing a plan pre-discharge, (2) Transition to community: forming relationships and goal setting, (3) Try out: encouraging problem-solving and managing practical issues, (4) Transfer of care: developing long-term goals and transferring responsibilities to community providers.

Outcome measures Progression criteria: recruitment, retention, acceptability of the processes (CTI and trial method) and fidelity. We also assessed the completeness of primary, secondary and exploratory outcome measures and estimated intervention costs.

Results The recruitment progression criterion was met with 92% of approached individuals agreeing to participate (target: 50%). Retention was poor (18% at the final follow-up, against a 60% threshold) impeded by a reduction in the support provided by the probation service. The process evaluation showed that while the CTI model was acceptable to staff and service users, the trial design was not. Staff had concerns over randomisation, exacerbated by the lack of support usual care provided. Intervention fidelity met the progression criteria. It is feasible to collect health economics outcomes and resource use at baseline, as well as the cost of the CTI intervention.

Conclusions Findings demonstrated substantial challenges, particularly around retention, trial acceptability and systemic support, indicating a full-scale RCT is not viable at this time.

Trial Registration: ISRCTN (Reference: ISRCTN46969988).

Funding: National Institute for Health and Care Research (NIHR), grant number NIHR134281.

Keywords: Homelessness prevention, prison discharge, randomised controlled trial, housing-led critical time intervention (CTI)

Strengths and limitations of this study:

DRAFT

For peer review only

For peer review only - http://bmjopen.bmj.com/site/about/guidelines.xhtml

1. The study employed a pilot parallel two-arm individual-level RCT, including a process evaluation and embedded exploratory health economic evaluation, which provides a robust framework for assessing the feasibility and acceptability of the intervention.
2. The study faced significant recruitment and retention issues, with only 18% of participants providing follow-up data at any time point.
3. There were ethical concerns and practical challenges related to randomisation, particularly regarding the acceptability of random allocation to the intervention or usual care, which affected the trial's implementation.
4. The study encountered logistical challenges, such as delays in approvals and difficulties in maintaining contact with participants post-release, which impacted the overall execution of the trial.
5. External factors, such as delays with Clinical Research Nurses, changes in prison resettlement and post-custody supervision arrangements, posed significant barriers to recruitment and retention, affecting the study's feasibility and outcomes.

DRAFT

For peer review only

For peer review only - http://bmjopen.bmj.com/site/about/guidelines.xhtml

DRAFT V.04 26.11.24

MAIN TEXT [words 9174]

Introduction

For people leaving prison, re-integrating into the community can be a difficult process. The absence of suitable affordable housing and a stable support network, in the context that many may have complex needs linked to substance use and mental ill-health, makes people leaving prison more susceptible to homelessness[1-5]. The absence of stable accommodation can then have further repercussions – people experiencing homelessness are at a higher risk than the general population of infectious and noncommunicable diseases[2], mental health problems, alcohol and substance use[3], have higher rates of hospital admissions[4] and report lower levels of wellbeing and health-related quality of life[5, 6]. Poor nutrition, inadequate self-management of preventable conditions and poor adherence to medications contribute to an all-cause mortality rate which is between 3 and 11 times higher in people experiencing homelessness, compared to other socio-economically disadvantaged people[7, 8]. Health outcomes are substantially worse amongst people with a history of both imprisonment and homelessness than those in isolation[9]. The detrimental consequences of homelessness following imprisonment can lead to a huge societal burden in terms of healthcare and societal costs[10].

Critical Time Interventions (CTIs) are focused and time-limited case management interventions that aim to improve an individual's engagement with treatment and community services through developing problem-solving skills and are targeted to continuity of care during a period of transition (e.g., release from prison)[11]. The key elements of CTIs are small caseloads, active community outreach, individualised case management plans, psychosocial skill building, and motivational coaching, with a phased approach that decreases in intensity over time[11-12].

There exists some evidence that CTIs improve housing stability outcomes in people experiencing homelessness or groups that are vulnerably housed[13-15], although these studies are not focused on prison discharge nor based in the UK. Evidence suggests there is a limited impact of CTI on improving health outcomes[11] and on quality of life[15-16]. A US-based cost-effectiveness study found the CTI to be cost-effective compared to the status quo for people experiencing homelessness with severe mental illness[17]. We identified that gaps in the evidence base included Randomised Controlled-Trials (RCTs) across multiple sites with a robust economic evaluation. The lack of UK-based studies also limits the applicability of evidence given international differences in the health care and criminal justice systems. A UK-based RCT focusing on CTI in prison populations experiencing homelessness is warranted due to the limited data within a UK setting, as well as a lack of RCTs conducted with people experiencing

For peer review only - http://bmjopen.bmj.com/site/about/guidelines.xhtml

homelessness and prison leavers. Our overall aim was to conduct a pilot RCT in male prisons to determine whether a future full-scale RCT of a housing-led CTI is feasible.

Methods

Study design and participants

This pilot study undertook a parallel two-arm, individual-level RCT of a pre-existing CTI intervention. The pilot included a process evaluation and embedded exploratory health economic evaluation. This pilot study is reported following the CONSORT extension for pilot and feasibility trials [18]. It was conducted in four prisons across England and Wales, housing adult males, with the intention to recruit 20 participants from each prison. The locations were predetermined by where the intervention was already being delivered by the intervention provider (CTI team).

Participants were eligible for the trial if they met all inclusion criteria and none of the exclusion criteria set by the organisation delivering the intervention. Included individuals were aged 18 years or over; due to be released into the local authority areas covered by the intervention delivery team, able to access benefits and local authority housing assistance; and had experienced prison and homelessness at least once. Individuals were excluded if they were deemed high risk to the public by probation and/or eligible for or receiving Housing First (i.e., support needs too high/complex to benefit from CTI).

Randomisation and blinding

Randomisation was completed after baseline data collection. Individuals were randomised on a 1:1 ratio and stratified by site, using random block sizes. A randomisation protocol tested the process and the system. Due to the prohibition of using electronic equipment in prison settings, randomisation was completed using concealed paper envelopes. Recruitment teams received a box containing 60 sealed sequentially numbered envelopes (a total of 4 boxes). Envelopes contained the arm allocation and participant identification number (PID) number. Boxes and envelopes were clearly labelled with the prison name and sequence number. Training was provided to recruitment staff to outline the randomisation method.

Data collection

Baseline measures were collected from October 2023 to August 2024, with measures intending to be collected at 3-, 6-, 9- and 12-months post-randomisation. Baseline data collection took place before randomisation within prison settings with follow-ups intending to be conducted at probation offices, at the intervention delivery team's offices and over the telephone. We intended to use Clinical Research Nurses (CRNs, registered nurses specialised in managing and

For peer review only - http://bmjopen.bmj.com/site/about/guidelines.xhtml

DRAFT V.04 26.11.24

conducting clinical trials and research studies, primarily in healthcare settings) in England but due to logistical challenges, this did not occur. In Wales, trial data collection was completed by the local R&D delivery team (the Welsh version of CRN) while data collection in England was completed by the research team. Probation services were intended to help track people leaving prison and follow-up data collection.

Observations of the intervention delivery across various phases, and interviews with staff delivering the intervention (from across both sites), examined intervention acceptability, delivery and implementation as well as explored the acceptability of conducting RCTs with this population. Cost logs from the intervention delivery team examined the time and resources spent implementing the intervention. We intended to interview trial participants (control and intervention), but this was not achieved.

Ethics and consent

The study received NHS ethics approval from Wales REC 3 (22/WA/0347), NRC approval (the National Research Council governs research within prison and probation settings), and permissions from each prison Governor and Probation Regional Manager. The study was registered in ISRCTN (46969988). Access to routine data via the Secure Anonymised Information Linkage (SAIL) Databank was granted by the SAIL Information Governance Review Panel (SAIL ID:1365).

Potential participants were referred to the CTI team for eligibility screening by the Prison, Community Offender Management Team or a self-referral. The CTI team would engage the individual and complete their referral assessment. Those deemed eligible were provided with a brief verbal explanation of the trial with the support of a summary information sheet and asked if they were interested in taking part. For those interested, prison visits were organised for the study to be explained, information sheets and consent forms to be signed and, following informed consent, baseline data collection occurred.

Patient and Public Involvement (PPI)

Patients and the public were first involved in the PHaCT study during the early stages of research design. An individual with lived experience of homelessness contributed to the design phase when applying for funding. The research questions were developed to align with the needs and experiences of the population at risk of homelessness. The involvement of individuals with lived experience ensured that the study addressed relevant and pressing issues, making the research more impactful and meaningful.

DRAFT
For peer review only

Intervention

The intervention is described following the Template for Intervention Description and Replication (TIDieR) guidelines[19].

Name and a brief description

Figure 1 shows the logic model for the CTI intervention. CTI is a psychosocial individual-level intervention that facilitates integration and continuity of care during a transition from institutions to community settings[20]. This CTI emphasises a "housing-led" principle which prioritises securing stable housing for clients as soon as possible to provide a platform to address other wider social or health needs.

Why, the rationale of essential elements

The CTI approach supports individuals by facilitating long-term ties to services, family, and friends through providing time-limited, emotional, and practical support during the transition. Those receiving CTI create a personal development plan and identify short-medium-long term goals depending on their individual needs, to develop self-management skills, and feelings of empowerment and improve their health and quality of life. Supporting individuals to secure housing is key as it provides stability, removing practical and emotional issues tied to experiencing homelessness.

What, a description of materials and procedures

CTI involves a pre-engagement phase. Starting before discharge, an assessment panel meeting identifies those at higher risk of homelessness. The caseworker meets the client and begins a process of engagement and relationship building. They assess the client's needs and implement a tailored (and agreed) transition plan to link the client to services and supports in the community. After the client is released from the institution, they are supported into a tenancy (immediately or following a brief period in temporary accommodation). This is followed by three phases (lasting 3 months each):

1. Phase one ("Transition to the community"): an intense period of forming links and relationships and shared goal setting; aiming to improve crisis-resolution skills, provide support and advice tailored to their needs, and mediate any conflicts. This phase involves weekly home visits and other meetings with the client, the client's caregivers, and community service providers.
2. Phase two ("Try out"): The caseworker monitors and adjusts the systems of support that were developed during phase one. This phase involves fewer meetings with the client, as the caseworker, with the help of community resources and family members, encourages

For peer review only - http://bmjopen.bmj.com/site/about/guidelines.xhtml

DRAFT V.04 26.11.24

the client to problem-solve and manage practical issues (e.g., sorting bills and general money/benefits management; living skills; social support and meaningful use of time; managing housing provider relationship). At this point, the caseworker intervenes assertively only if the client is receiving inadequate support or if a crisis occurs.

3. Phase three ("Transfer of care"): the caseworker reduces the level of support and begins to 'step away'. They help the client to develop a plan to achieve long-term goals (e.g., employment, family reunification) and finalise the transfer of responsibilities to caregivers and community providers (i.e., the CTI intervention is terminated with support in place).

Who delivers the intervention?

The intervention was delivered by CTI caseworkers based in a UK charity; specific teams focused on supporting those leaving prison to secure housing.

How, are modes of delivery?

CTI caseworkers met with people in prison and upon release in office settings (CTI or probation offices), in the community or at a residence. Caseworkers would work from CTI offices or at prison and probation locations.

Where, locations where intervention has occurred?

England and Wales.

Intervention funding

The intervention is funded by a UK charity.

Outcome measures

The primary outcome of the pilot RCT was to determine whether a full-scale RCT of the intervention was feasible in terms of progression criteria (Table 1). These criteria sought to assess acceptability and fidelity in the delivery of the intervention and RCT design with thresholds set according to a traffic light system. If progression criteria were green, it would indicate the study should progress to a full-scale evaluation. That said, criteria are applied with discretion so it may be that a study progresses if a method of improving an amber criterion is identified.

We also examined the levels of missing data and distribution of the indicative primary outcome, health-related quality of life, measured using the validated SF-12 [21-22]. The secondary indicative outcomes included housing stability - measured by 1) number of days in settled accommodation (stable, long-term housing where individuals can live securely without facing imminent risks of eviction or displacement), 2) capability well-being via the validated measure

For peer review only - http://bmjopen.bmj.com/site/about/guidelines.xhtml

ICECAP-A[23] and 3) EQ5-D[23]. Hazardous alcohol consumption was measured via the validated AUDIT-C questionnaire[24] and substance use was measured via ad hoc questions. Deaths were recorded throughout the study including cause of death.

Our exploratory outcomes assessed whether participants consented to link to routinely collected healthcare and criminal justice data. Of those who consented, we also sought to explore the proportion of people who could be successfully linked to their healthcare records, via SAIL/NHS England, and criminal justice records, via the Ministry of Justice Data Lab, by applying to use these data linkage services. Attempting 'test linkages' sought to inform the potential use of data linkage to provide additional secondary outcomes, such as re-incarceration, re-conviction, education, training and employment, and welfare benefits receipt.

Analysis

Statistical analysis

The primary analysis sought to determine whether the pre-specified progression criteria were met. The analyses were primarily descriptive, providing estimates of recruitment, response and retention rates. Recruitment, randomisation and retention of participants at follow-up were summarised in a CONSORT flow diagram (see Figure 2). We tabulated demographic characteristics of participants within settings by study arm (intervention or control) and assessment time point (baseline or follow-up) using descriptive statistics means and standard deviations (or medians and interquartile ranges [IQR], as appropriate) for continuous outcomes, and frequencies and percentages for discrete outcomes. Analysis was performed in Stata version 18[25].

Qualitative analysis

Qualitative data generated through semi-structured interviews were audio-recorded, transcribed and coded. Field notes from observations and free-text entries in logbooks were coded using a similar system. Members of the research team (NJ, AW) analysed the data using inductive and deductive thematic analysis[26]. A coding scheme was developed by the researchers. The coding scheme evolved during analysis, with the new codes discussed and confirmed by the team, before being applied to previously coded data. NVivo 12 supported data storage and analysis[27].

Fidelity to the CTI model was measured against five fidelity items: housing-led approach, time-limited and phased approach, caseloads and supervision, person-centred approach/community focussed, and harm reduction/recovery orientated approach. From the interviews and

DRAFT
For peer review only

DRAFT V.04 26.11.24

observations, a score was provided: (Low=1, Medium=2, High=3) against each fidelity item with an overall score calculated.

Economic evaluation

We reported descriptive statistics for health economics outcomes (EQ-5D-3L and ICECAP-A) and resource use by collection time point and intervention group. Quality of life and capability well-being scores were compared with reference values for the general population. The cost of delivering the CTI intervention was captured by adapting a tailored cost log to the existing data collection system used by the charity delivering the intervention.

Results

The study faced extensive delays that affected recruitment timelines, beginning with approval and contractual issues. The study was planned to open within six months, but this was extended; site 1 began recruiting after 16 months, while site 2 began after 22 months. Delays included a prolonged NRC approval process and difficulties in obtaining accurate contact details for relevant HMPPS staff to gain approval. Site 2 faced staff shortages, halting recruitment to the intervention for a prolonged period. The study encountered issues with the CRN teams, whose contracts were delayed due to site-specific requirements in the prison setting. Initially, CRN teams were assigned to handle all recruitment and follow-up at sites 1 and 2, but contract signoffs were unexpectedly required even after initial discussions suggested they wouldn't be needed. At site 2, unforeseen delays led to a protocol amendment allowing the research team itself to take on recruitment and follow-up. However, as the team was not local, recruitment and follow-up required intensive planning and clustered visits, limiting participant engagement opportunities.

In total, 34 individuals were recruited in the study across three sites (one site did not recruit) from an intended 80 participants (that is, 43% recruited from the target of 80 participants). Fifteen individuals (44%) were randomised to the control arm and 19 (56%) to the intervention arm (see Figure 2, consort diagram). Of the recruited participants, the mean age was 38.1 years (standard deviation (SD): 11.2 years, min: 19, max: 67), and 9% were from ethnic minority backgrounds - comprising Mixed or Multiple ethnicities, Bangladeshi, and Caribbean or Black. Overall, 35% (n=12) reported to not have any formal educational qualifications. Illicit drug use in the period of 12 months before entering prison was reported by 91% (n=31) of participants. Participants reported experiencing homelessness on average 3 times since the age of 16 [IQR: 2-4], with 74% (n=25) reporting they were not in settled accommodation before entering prison.

Progression criteria

For peer review only - http://bmjopen.bmj.com/site/about/guidelines.xhtml

Table 1 summarises the results of the pilot RCT against the progression criteria. Two of the four progression criteria were rated green (recruitment and fidelity). The process evaluation was rated amber, with retention receiving a red rating.

1. Recruitment

Figure 1 shows the CONSORT flow diagram. CTI caseworkers referred 61 people to the research team but 24 people were unable to be approached within the prison setting (so could not be recruited); reasons included early release (12/24, 50%), moving prison (7/24, 29%), not being accessible within the prison, i.e., held in cell for misbehaviour, attending other meetings/prison visit (5/24, 21%). Of the 37 approached, 34 (92%) agreed to take part (the 'green' threshold for this criterion was ≥50%); for the three who refused, the reasons included distrust of research and experiencing mental health difficulties.

Table 1. Summary of results against the progression criteria

Progression Criterion	Red	Amber	Green	Actual
1. Recruitment: 50% of those approached agreed to participate.	<40%	40-49%	≥50%	92%
2. Retention: 60% of those recruited are retained at final follow-up.	<50%	50-59%	≥60%	18%
3. Process evaluation: the process (CTI and trial process) is acceptable for participants and staff.	Neither is accepted by the participant or staff	CTI or trial process is accepted by participant or staff	Both are accepted by participants and staff	CTI = acceptable Trial = not acceptable
4. Fidelity: the intervention is implemented with fidelity in all settings.	Fidelity score <20	Fidelity score 20-25	Fidelity score ≥25	Overall score = 26

Throughout the study period, the early release from the prison scheme (End of Custody Supervised License scheme, ECSL[28]) posed challenges for recruitment. Across all sites, individuals referred by CTI caseworkers were being released before the recruiter was able to attend the prison. In some cases, the referral was received on a Wednesday and by the following Monday the individual had been released. Recruiters' experiences also highlight the chaotic

For peer review only - http://bmjopen.bmj.com/site/about/guidelines.xhtml

DRAFT V.04 26.11.24

nature of the release as one participant was informed on the morning they were recruited that they were being released the following day. This provided no time for accommodation to be organised or approved by probation even if a location was known, such that individuals could be released homeless.

An additional challenge to recruitment was the high number of inappropriate referrals from prison and probation. Conversations with CTI caseworkers indicated that many referrals were not appropriate for the intervention, or the referral did not include all relevant information. The risk category of offenders was often missing, due to lone working the CTI caseworkers cannot work with anyone categorised as high-risk. The lack of appropriate referrals delayed the recruitment as everyone was assessed by a caseworker after a referral, and only so many people could be assessed in a single prison visit. When information was missing from referrals, this further delayed the recruitment process.

The high level of agreement to take part led to the research team questioning whether some participants may have been unintentionally compelled into agreeing to participate simply because they were in a setting conditioning them to agree (prison). The CTI caseworkers felt similarly, but that the agreement to take part resulted from a mix of a lack of understanding and desperation for support.

> "I'm not sure they fully understand what they're consenting to. And I don't think it's a capacity thing. I don't think explaining it anymore could actually make a difference; I just think it's not their priority. Erm, and they're so desperate, they'll agree to anything that they think might help them." *CTI caseworker, site 1*

However, discussions with experts in the field of prison research expressed that high engagement was common due to a desire to engage with people outside of the prison setting, particularly if it provided additional opportunities for them to leave their cells or the wing itself.

2. Retention

Of the 34 participants recruited, there was a death which occurred before follow-up, three withdrawals, and eight participants returned to prison, leaving 22 people to attempt follow-up. Six of the 34 recruited participants had follow-up data for at least 1-time point (18%, the 'green' threshold was 60%), two participants were allocated to the control group and four to the intervention. At 3-months, three participants were followed up (9%, 3/34). Similarly, at 6 months, three participants were followed up (9%, 3/34). One participant was followed up at 9 months (3%, 1/34). One participant was followed up for two time points, the rest were followed up at a single

For peer review only - http://bmjopen.bmj.com/site/about/guidelines.xhtml

time point. Due to delays at trial initiation, no participants achieved the 12-month follow-up point. These delays were related to the time taken by the NRC to consider our application and approve amendments in response to delays and unexpected contractual requirements by the Clinical Research Network to work within prisons. Of the 22 participants who could be followed up, eight (8/22, 36%) did not provide any contact details, and those who did provide contact details (email, telephone or both) were contacted four times at each follow-up point. For many of the participants who provided a telephone number, it had been disconnected when contact was attempted (8/14, 57%).

Our methodology involved maintaining contact with participants post-release via their probation practitioners, to whom prison leavers would have to report regularly for no less than 12 months post-release, following the Offender Rehabilitation Act (2014). However, progressively lower levels of staffing in the probation service and higher caseloads for those that remained posed significant barriers. Of our participants, 12/34 (35%) did not have any details for their probation practitioner at baseline, 12/34 (35%) had only a name with a partial address, and 10 participants (30%) had a name and contact information for their probation practitioner. Throughout the study period, the strain on the Probation Service following the ECSL and Probation Reset scheme resulted in many of our participants not having contact with probation staff[28,30-32].

Maintaining engagement proved difficult as many participants had competing and complex support needs, marked by frequent issues such as homelessness, substance use and re-arrest/imprisonment. Although meetings were scheduled for follow-up data collection and qualitative interviews, participants routinely missed these appointments. Early in the study, CTI caseworkers noted that engaging with participants in the control group, who would not receive CTI support, would be especially difficult. They felt that without the benefits of the intervention, and with no material incentive permitted, these participants would potentially be less motivated to stay in contact or respond to researchers' outreach efforts.

> "The people that go into the control group, you're going to try and check in with them at 3, 6, 9 months, I don't think that's going to work. They're not interested now, we've offered them nothing, they will not pick up the phone." *CTI caseworker, site 1*

3. Process evaluation

The process evaluation aimed to assess the acceptability of the CTI model and the trial methodology, to both participants and staff delivering the intervention.

<u>Acceptability of the CTI model</u>

For peer review only - http://bmjopen.bmj.com/site/about/guidelines.xhtml

Early in the evaluation, it became evident that the CTI was highly valued by the staff, who shared numerous success stories attributed directly to the intervention. The widespread acceptance of CTI introduced challenges related to the trial methodology, particularly around the acceptability of random allocation to the intervention delivery staff. It became clear that intervention delivery staff believed the intervention was already effective. This is likely to be similar to other social policy interventions where those delivering it will take the view that the intervention will generate some positive outcome.

> "I do firmly believe it works. I think housing-wise because of the state of the market, it's not as successful KPI-wise on paper as I'd like it to look. But I think the softer outcomes outweigh that every time, like having people feel supported, feeling like there's people there for them. I think that motivates people to stop reoffending." *CTI caseworker, site 1*

> "I think when it works, it works amazingly well." *CTI caseworker, site 2*

Observational data highlighted that many service users expressed a high level of acceptance of the CTI model. Individuals who were housed expressed gratitude for the support provided. The phased approach was deemed appropriate as service users became settled, expressing their agreement with having less contact once settled. A minority of service users challenged the provision of CTI, wanting greater support than the model provided and to be provided accommodation rather than search for their own accommodation.

Due to the challenges retaining participants, we were not able to conduct interviews with trial participants which limits our understanding of the acceptability of the intervention to participants. We intended to interview service users not part of the trial but receiving the intervention. Service users would agree to be observed but not interviewed.

Overall, the intervention (CTI) was deemed acceptable by both staff and service users (based on observations), but our findings are limited by the lack of trial participants able to be interviewed with acceptability assumed from service users continuing to engage with the intervention. Those who found the model unacceptable likely did not engage with the CTI caseworkers and hence could not be observed.

<u>Acceptability of the trial process</u>

CTI caseworkers highlighted concerns around trial methodology, particularly regarding randomisation. Many staff viewed the randomisation element of the trial as unethical as it would prevent a highly vulnerable population from accessing the intervention without providing

For peer review only - http://bmjopen.bmj.com/site/about/guidelines.xhtml

alternative support. Site 1 had strong concerns regarding the fairness of a control group due to the limited services that make up usual care.

> "…we're dealing with people and not just people but people's lives and if they don't have support coming out of prison this might be the time that they take too many drugs and they die, and I don't feel comfortable being any contributing factor to that." *CTI caseworker, site 1*

> "… the whole point of CTI being [here] is because there's not any support like it. So when they're saying like all the usual care, that's next to nothing basically." *CTI caseworker, site 1*

The concerns around those receiving usual care led to both sites attempting to refer control arm participants to other services delivered by their organisation as participants released from prison without support would be signposted to their services as part of usual care.

Informed consent was also questioned by the CTI caseworkers due to concerns about the vulnerability of the prison population. CTI caseworkers felt potential participants struggled to understand concepts like randomisation and research in general.

> "But the reality of the population here, is they don't know what a randomised control trial is. They don't understand what research is. And a lot of them have got like, a lot of them didn't go to school, a lot of them can't read and write. It's not really realistic to have that kind of in-depth conversation." *CTI caseworker, site 2*

> "I've had one guy who is actually in the intervention group now, he kept saying to me how do I get into the group with your support and I was like, it's randomised, there's nothing you can do and he's like what shall I say in the answers, and I'm like, it's randomised, there's nothing, can you put a good word in for me and I was like, it's not up to me, I'm really sorry. You go in and you tell the truth and then you find out." *CTI caseworker, site 1*

Multiple concerns surrounding the trial process were continually raised by CTI caseworkers and the lack of data from participants led to the conclusion that acceptability of the trial process was low.

The high acceptability of the CTI model but low acceptability of the trial process, results in this process evaluation acceptability scoring being amber. The 'amber' threshold was 'CTI or trial process is accepted by participant or staff', see Table 1.

4. Fidelity

For peer review only - http://bmjopen.bmj.com/site/about/guidelines.xhtml

For peer review only DRAFT

Table 3 presents the summary of the fidelity scores across sites.

Table 3. Fidelity scores across Sites.

	Site 1	Site 2	Overall
Housing-led approach	2	3	5
Time-limited and phased approach	2	2	4
Caseloads and supervision	3	2	5
Person-centred approach/ community focussed	3	3	6
Harm reduction/recovery-orientated approach	3	3	6
Overall	**13**	**13**	**26**

Housing-led approach: the model of CTI adopted for the intervention emphasises a housing-led principle that prioritises securing stable housing for clients as soon as possible as a platform for addressing other wider social or health needs. In adherence to the housing-led approach, CTI caseworkers prioritised finding stable housing for clients from the outset. Housing was a focus of all conversations observed, from the first meeting with clients in prison during the pre-CTI period to all subsequent follow-up appointments where housing was yet to be secured.

To be true to a housing-led approach is to say that priority should be given to enabling people to access settled housing as quickly as possible and given support to sustain it. For site 1, supporting people to gain a tenancy was a major focus but the dire housing situation of site 1's location meant that individuals could not have immediate or speedy access to housing. Thus, the team adhered to the housing-led philosophy, but the structural context made delivery problematic. For this reason, they scored a medium on fidelity to being housing-led.

> "…we'll chase up the housing applications and then we'll meet them on the day of release to go with them. At the moment in [Site 1 area], it's working that you have to go to housing options still on the day of release, but we can meet them at probation or there and obviously vouch for them because quite a lot of ours have been told, oh you need to find somewhere to stay, stay with a friend and we've had to go back in with and be like, they don't have a friend. There is no other option. If they're not given temporary, they'll be sleeping on the beach tonight. They get temporary and then from there, at the moment it's working that from there we start looking for, we register them with social landlords, we start looking for private landlords and then help them get like the rent, the bond, we work out what their budget is and then linked to that if they want to go into employment or education we look at that, at the same time." *CTI caseworker, site 1*

DRAFT
For peer review only

“No, like [our KPIs are] how many people have been housed. Is their housing sustained? But our progress is that he’s come to three appointments this week. Like or he’s gone a week without using. And that’s huge in CTI.” *CTI caseworker, site 1*

“Because we are in such a state obviously like everywhere with housing. Nine months is not a long time to get accommodation. So it’s making them obviously feel settled where they are if it’s in temp and then if there are any other groups in the community which they are interested in.” *CTI caseworker, site 1*

For site 2, housing was prominent from the beginning of the intervention and moving individuals into their own, private rented accommodation was a priority. The focus of initial discussions centred on making sure someone has the resources available to them to sustain a tenancy.

“…will they be able to keep up with the rent and keeping the flat and everything else that goes with it.” *Observation 15.5, site 2*

“Are you looking for work, are you putting in a claim for disability benefits … cos you’re not, you’re never affording a tenancy on your own.” *CTI caseworker, site 2*

While the housing market in site 2 was also challenging, there was greater availability of homes. Observations at site 2 witnessed the close connections with various private property owners throughout the area, with property owners contacting CTI caseworkers individually to inform them of new available homes which were then shared among the team. This resulted in faster results in getting individuals into settled accommodation, with site 2 able to provide costs to cover the bond and first month’s rent for new tenants to speed up access. Additionally, site 2 could sometimes cover or pay off bill debts that may occur if a service user was recalled to prison for a short sentence, support service users to ensure their benefits could be maintained and assist with rental payments to allow tenancies to be retained during short prison recalls. In site 2, the team here managed in practice to move participants into settled housing at a faster rate. For this reason, site 2 scored a high on being housing-led.

The contextual challenges around housing were compounded by guidelines and restrictions on who could access certain housing. Across both sites, there were restrictions on people leaving prison or those with criminal records on their ability to access Registered Social Landlords (RSL) properties via the allocation processes. In Wales, RSLs manage a significant portion of social housing stock meaning this population cannot access most of the social housing. Despite vulnerable prison leavers with a local connection being identified as a priority need group for

For peer review only - http://bmjopen.bmj.com/site/about/guidelines.xhtml

DRAFT V.04 26.11.24

housing in Wales. Current restrictions within social housing result that even if prison leavers are given priority, they will still be denied certain social housing,

Time-limited and phased approach: CTI should be implemented in phases: with a pre-CTI phase, followed by the main phases of the intervention, transition to the community, try out, and transfer of care, each main phase lasting three months. The time-limited nature is a key focus of the intervention. For both sites, fidelity to the time-limited and phased approach was routinely adhered to, with the three-phase structure clearly guiding the transition process for participants.

> "We stick to it rigidly. So when the nine months is up we obviously discuss it leading up to the last phase, it is a topic of conversation that we have so anything that's identified, you know hopefully by that time they either settle in their own accommodation or they are very high in the list to be offered accommodation." *CTI caseworker, site 1*

> "[During phase 1] we'll go and see them every week, for the twelve weeks and then we sort of, step right back then. During that time, we should have explored the local area, to go like, I don't know, there's a community centre round the corner, or there are your local GPs, and there's your sports centre, or there's a library there, or there's a men's group round the corner, there's a walking group there... so, we step right back then and by that point, they should have everything in place. And, then I'll go and see you every two weeks then [during phase 2]. And then the last phase is once a month." *CTI caseworker, site 2*

However, both sites also indicated that there could be instances where caseworkers would extend support, outside of the intended 9 months, if they felt it was necessary.

> "If it's the case that that individual's needs change because something's happened, I don't think we'd necessarily stick to it because that's what the model says. If there's a live event where that individual just needs a little extra assistance at that time, then I don't think as a service we could justify not doing that." *CTI caseworker, site 1*

> "Interviewer: going back to you thinking about decreasing support, so, that's totally up to you, whether or not they are able [to continue without support] and then if it only lasts for nine months, what happens at the end of that nine months?
> Interviewee: We can extend, if we feel we need to, we can extend it [the intervention]. *CTI caseworker, site 2*

Adapting the level and intensity of support through the phases was observed at both sites, with more frequent meetings at the initial phase, and less frequent check-ins later on as stability was achieved.

For peer review only - http://bmjopen.bmj.com/site/about/guidelines.xhtml

The pre-CTI phase is the initial stage of the intervention, where the focus is on caseworkers developing a trusting relationship with the client. Conducted while the client is still in custody, this phase allows caseworkers to build rapport, tailor support and ensure that essential needs – such as applying for benefits – are addressed. However, discharge from prison is often reported to be unpredictable and unplanned[1,7], making this the pre-CTI phase challenging from the offset. The introduction of the ECSL scheme resulted in even more disruption to the pre-CTI phase across both sites. Without this groundwork, caseworkers reported that establishing trust and tailoring support became difficult, often leading to releases where vital needs, such as connecting to support services and securing benefits, went unmet.

> "CTI is meant to be a set amount of time, and we go in and see them every week. We've had referrals pass to us, we've gone, and they've already left prison. So that means those people have been missed, so they've just gone." *CTI caseworker, site 1*

> "A key element of CTI is the sort of pre-CTI phase, which is meeting with members whilst they're in custody. And I think that's really important because you've almost got this pinch-point on release where there's so much to do, potentially individuals who perhaps haven't got the coping mechanisms, you know, it's probably at that point where they need that intense support, and it's an opportunity to build a bit of rapport, a bit of trust and things like that as you're going through the various things that need doing." *CTI caseworker, site 2*

Both sites broadly complied with the phased approach and generally reduced support over time, but some deviation was present if caseworkers felt additional support was required. Sites were hampered by the same contextual issues which limited their ability to provide the pre-CTI element of the intervention as intended, thus both scored medium for fidelity.

Caseloads and supervision: Key principles of CTI are small caseloads, team-based supervision and frequent case reviews. The training slides for the provision of the CTI intervention from the intervention delivery team indicated intended caseloads of 20 or fewer, monitored by a case management system. A meta-analysis of previous studies identified that the average number of cases within a CTI caseload is 25 per caseworker[29]. Team-based supervision allows for a collaborative approach where team members work together to provide comprehensive care. Frequently reviewing cases helps to adjust and improve the intervention strategies as needed.

In site 1, there was only one prison with a smaller capacity providing referrals, this resulted in average caseloads for site 1 being 15 throughout the trial period. In site 2, there were three

For peer review only - http://bmjopen.bmj.com/site/about/guidelines.xhtml

prisons of larger capacity providing referrals. The CTI caseworkers reported caseloads of between 18 and 28 cases on average (for some caseworkers, caseloads included both the intervention being researched and a shorter community CTI model lasting 6 months).

> Discussion indicates that the maximum caseload is 40, “but we never get that many", with caseloads generally being between 18 and 28. *Fieldnotes, site 2*

> "I've got 33 cases at the moment, but a few of them need to be closed but as a list, it's 33." *Fieldnotes, site 2*

Observations identified that team-based supervision and frequent case reviews were key features at both sites. Caseloads were managed at each Site during weekly formal supervisory meetings where the progress of each beneficiary was reviewed considering any key events that might have happened. This was essential to supporting staff during difficult experiences, such as the death of a service user.

> “with that whole process, CTI team meet weekly, so that is quite, quite an intense type of meeting really just to manage referrals, but also there’s then discussions of people who are actively being assisted in the service as well.” *CTI caseworker, site 1*

> “I’ll also look at how that caseload is with [name] and talk through any particularly challenging cases, and hopefully um, you know, come up with a plan in terms of moving things forward if, you know, sometimes you might feel a bit stuck with some complexities, um so I do that. Um and then yeah, just try to work really um, I suppose collaboratively with [name], um making sure that, you know, I’m up to speed with as much as possible around what’s going on with CTI.” *CTI caseworker, site 1*

Both sites were observed to meet the key principles of team-based supervision and frequent case reviews. Site 1 maintained small caseloads resulting in them receiving a high-fidelity score, while site 2 received a medium score due to the potential to go over the allotted 20-25 cases.

Person-centred approach/ community focussed: The person-centred approach of CTI includes personalised goal setting, strengthening social support networks, providing flexible, needs-based support, and empowering through skill-building. Observations showed collaborative goal-setting between CTI caseworkers and service users, focusing on priorities like employment, housing stability, and social connections. This process empowered individuals to take ownership of their transition, with caseworkers helping them establish practical steps towards their goals. Observations found that staff across both sites were proactive in delivering a person-centred approach with a community focus.

DRAFT

For peer review only - http://bmjopen.bmj.com/site/about/guidelines.xhtml

Observations witnessed that CTI caseworkers assisted with both everyday and complex issues, such as accessing phones, setting up bank accounts, engaging with substance misuse services, and securing housing. They supported individuals in navigating complex systems like housing, healthcare, and employment services, and advocated on their behalf when barriers arose. This included accompanying service users to appointments, assisting with paperwork, and negotiating access to services.

> Explanations were provided of the various community needs faced by many people CTI caseworkers support, these include health, economic, social, environmental, and cultural. Various barriers were reported that prevent people from achieving community integration and well-being. Some examples included not having a bank account or bank card, having no GP difficulty accessing required healthcare, and having difficulty gaining employment. *Fieldnotes, site 2*

During early phases, it was observed that caseworkers taught service users how to search for properties, use websites and filters, and draft emails to property owners. They attended property viewings with service users or provided support remotely if they couldn't attend. They also suggested bringing a family member if they were unavailable.

> A service user was living in their car and facing having their case closed by the local council due to part owning a home with their partner. However, due to their conviction, they could not return to the property. The CTI caseworker was the sole spokesperson striving to have the local council provide accommodation, setting up meetings with various relevant groups to deal with the situation on the service user's behalf. *Fieldnotes, site 2*

CTI caseworkers would help individuals connect or reconnect with family, friends, or community groups. This could include coordinating family meetings, linking individuals with peer support groups, or assisting with finding community-based programmes that fit their interests and cultural backgrounds.

> "then if there's any other groups in the community which they are interested in, just for argument's sake like art, if they've been doing like the art learning activities here we look for something in the community then that they can start going to prior to finishing with us. So they've still got that support, somewhere to go but they are not reliant on us either." *CTI caseworker, site 1*

For peer review only

DRAFT

For peer review only - http://bmjopen.bmj.com/site/about/guidelines.xhtml

DRAFT V.04 26.11.24

> "So, you're, during that time, we should have explored the local area, to go like, I don't know, there's a community centre round the corner, or there's your local GPs, and there's your sports centre, or there's a library there, or there's a men's group round the corner, there's a walking group there." *CTI caseworker, site 2*

Both Sites were identified to have high fidelity for this item.

Harm reduction/ recovery orientated approach: This approach in CTI focuses on minimising the negative consequences associated with certain behaviours (such as substance use without requiring complete abstinence) while supporting individuals in their recovery journey. It involves providing safer alternatives, education, and resources to help individuals make healthier choices. The goal is to support individuals in achieving their personal recovery goals, which might include stable housing, employment, and improved mental health.

During the referral process, potential service users were asked about their specific support needs, including inquiries about alcohol and substance use, physical and mental health and any other areas of support they might need. Plans being made to help connect service users with identified support services upon release from prison were observed. Additionally, the model did not require abstinence and thus CTI caseworkers openly discussed substance use. Where appropriate, goals around substance use started with reducing current use rather than beginning a conversation with abstinence as the first goal, highlighting the harm reduction approach.

While CTI caseworkers strived to meet housing needs, the nature of the housing market sometimes meant service users were moved into accommodation unsuitable to their recovery needs. The lack of housing, particularly in site 1, resulted in many service users being placed in temporary accommodation. This situation often necessitates an increased focus on harm reduction as longer periods in temporary accommodations with other substance users make it more difficult to maintain sobriety. Both Sites were categorised as high fidelity.

> "I think making it feel as if they are comfortable there and before that period, they've got somewhere to come to if they want to use anything that's going on here. We've always got something going on and that's what [organisation] are really good at is like there's always drop-in sessions, you know if they are having a bad day they can come in for a coffee. But they've got that support there and I think that's what makes a difference. If they haven't and they are spending all day every day in B&B, the likelihood is they've got an issue with substances, so they are going to start using again because they are surrounded by it in B&B as well or temporary accommodation. So if they can start doing

For peer review only - http://bmjopen.bmj.com/site/about/guidelines.xhtml

> things that fill their days up, that does help, it works well for them. The housing part of it is hard". *CTI caseworker, site 1*

> Discussions of the challenges in trying to keep service users engaged with support services - mainly addiction services. Caseworker provided an example of how they previously attended AA group sessions with a previous service user to support them to start going. *Fieldnotes, site 2*

Indicative outcomes

The indicative primary outcome of a future full-scale trial was to be health-related quality of life assessed using the SF-12. However, due to the limited retention, we do not have enough data to meaningfully report on this. The average EQ-5D score of participants at baseline is 0.57 (SD=0.42). The average ICECAP-A score of participants at baseline is 0.62 (SD=0.29). Based on baseline data, it was observed that 73.5% (25/34) of participants reported not being in settled accommodation before entering prison. The figure for not being in settled accommodation before entering prison was higher among the control group (12/13, 86.7%) than the intervention group (12/19, 63.2%), with more instances of homelessness (since the age of 16) among the control group (median=3.5, IQR=3 to 5) than intervention group (median=2.0, IQR=1 to 2). From the six participants with follow-up data, one participant was living with parents, one in an emergency B&B, one in a hostel, one in supported accommodation and two participants had returned to prison. The control group reported higher scores on the AUDIT-C in the 12 months prior to entering prison (9, IQR=0 to 12) compared to the intervention group (3, IQR=0 to 12). Resource use and employment and benefit receipt data are provided in Tables in the supplemental material. There was a single death throughout the study period. We found low rates of missing data for almost all variables. Overall, for the six participants who were followed up at various time points, there were no missing data in the SF-12, EQ-5D, ICECAP-A variables or resource use data, and less than 5% missing data in income and benefit variables, thus supporting the feasibility of the data collection instruments.

Exploratory outcomes

Of the 34 participants enrolled at baseline, all participants consented to data linkage to healthcare data sources, and to link to criminal justice data sources. Due to delays in recruitment, and as most of the sample were initially recruited from the Welsh study setting, test linkages using NHS England were not conducted. The intervention delivery organisation attempted to access the Justice Data Lab but was unable to use the service due to issues around the timings of data collection and not enough time had elapsed for one-year offending to have

For peer review only - http://bmjopen.bmj.com/site/about/guidelines.xhtml

taken place following exit from the CTI intervention. Test linkages were however attempted via the SAIL Databank. As SAIL only relates to people who have been residents in Wales, people recruited from sites in England were excluded from the test linkage (14/34, 41%). In total, data relating to 17 participants at baseline were sent to SAIL for matching. The results of the matching were not available by the time of publication.

Economic Evaluation

In consideration of the small number of participants at follow-ups, it was not possible to conduct the planned economic evaluation (i.e., cost-utility, cost-consequence, and cost-effectiveness analysis), or to present a meaningful comparison of participants' outcomes and costs by data collection time point. Instead, we provided a characterisation of the cost of the CTI intervention in terms of key cost components. Also, we used the baseline data to summarize the quality of life (EQ-5D-3L), capability wellbeing (ICECAP-A) and resource use of PHaCT participants.

In consideration of the substantial number of drop-outs and lost to follow-ups, only 2 participants out of the 17 randomised to the CTI intervention completed all the intervention stages. The total cost of delivering the CTI intervention in the PHaCT trial was £6,356. A breakdown of costs by cost category and intervention phase, as well as the average cost by intervention phase s are reported in the supplementary material.

The cost estimates should be taken cautiously due to the small number of observations and the substantial number of losses to follow-ups. Also, while we initially aimed to collect travel costs as both the actual cost sustained to travel (retrieved from travel claims) and the opportunity costs (i.e. time spent by staff travelling, multiplied by the hourly staff wage), it was not possible to collect the staff opportunity cost of travel to prison. Likewise, the actual cost of travelling does not include travel to prison. However, we show the feasibility of consulting with the CTI team as well as using and adapting data collection systems used by CTI caseworkers to capture the cost of delivering CTI.

The supplementary statistics show average EQ-5D-3L and ICECAP-A scores for PHaCT participants at baseline, as well as summary statistics on resource use. Overall, we showed the feasibility of using EQ-5D and ICECAP-A to collect information on the quality of life, capability wellbeing and resource use in the PHaCT people in prison population at baseline. However, due to low retention, we could not test the feasibility of collecting information on health economics outcomes and resource use at follow-ups.

Discussion

For peer review only - http://bmjopen.bmj.com/site/about/guidelines.xhtml

DRAFT V.04 26.11.24

In this pilot randomised controlled trial, only two of the four progression criteria were met suggesting there should not be a follow-on full-scale RCT of this housing-led CTI.

Three of the four planned sites opened to recruitment with 92% of individuals approached agreeing to participate, well above our target of 50%.The high level of agreement to take part led to the research team questioning whether some participants may have been unintentionally compelled into agreeing to participate simply because they were in a setting conditioning them to agree (prison). The CTI caseworkers felt similarly, but that the agreement to take part resulted from a mix of a lack of understanding and desperation for support. The planned recruitment process faced contextual challenges related to changes in prison resettlement and post-custody supervision arrangements. The End of Custody Supervised License (ECSL) scheme, was launched in 2023, allowing for people in prison to be released up to 18 days early. This was then increased to 35 days early in March 2024 and 70 days early in May 2024. Current records state that 10,003 individuals were released early from their sentences between October 2023 and June 2024[28], requiring accelerated responses from prison and probation staff. This significantly disrupted the recruitment process as interested individuals were often released before recruitment appointments could be scheduled, making it difficult to engage them in the study and having knock-on effects on retention.

Retention rates were notably low, with only 18% of participants providing follow-up data at any time point. Our methodology involved maintaining contact with participants post-release via their probation practitioners, to whom prison leavers would have to report regularly for a period of no less than 12 months post-release, following the Offender Rehabilitation Act (2014). This low retention rate reflected broader probation sector challenges, including staffing shortfalls and restructuring pressures that deprioritised lower-risk offenders (largely our target group under the Offender Rehabilitation Act).[30-32] Retention was further hindered by high licence recall rates; a sharp 17% rise in licence recalls led to constant movement in and out of prison, creating instability[33]. The introduction of the schemes mentioned which affected both recruitment and retention created a churning effect of people being sentenced, early released, recalled finishing a very short sentence then released again but without the need to be in contact or supported by probation.

The staff delivering CTI had widespread acceptance of the model but raised concerns related to the trial methodology, particularly around the acceptability of random allocation to the intervention or usual care. It became clear that CTI caseworkers believed the intervention was effective thus the concept of random allocation became extremely hard to justify, both because

For peer review only - http://bmjopen.bmj.com/site/about/guidelines.xhtml

staff were invested in the intervention and helping a vulnerable population. This disagreement with randomisation is common for most social policy interventions as it is difficult to argue the intervention does no good at all, with uncertainty focussed on the extent of any benefits and their relative cost-effectiveness[34]. This means that equipoise is likely unsustainable as the moral basis for social policy-based trials, thus justification must be focused on 'positive expected outcomes' and prioritising personal autonomy.

Reflections and Recommendations

Future research should consider the following recommendations to improve research in prison settings with this population:

Design and approvals: The study highlights the need for change to effectively deliver RCTs within this setting and with this population. The NRC need to remove the prohibition against incentives, as they will be an important step in maintaining contact with control group participants. Additionally, the NRC need to improve the process for gaining approvals as well as approving amendments to maximise efficiency and reduce delays. Developing alternative trial designs that address ethical concerns is crucial. One approach could be the use of quasi-experimental designs. Follow-up could be augmented by using routine data, given the high levels of consent to linkage found in this pilot study. However, the use of routine data would be limited to ‘hard outcomes’ such as healthcare diagnoses, mortality, and public sector service use within a very limited number of areas—mainly healthcare and criminal justice. An alternative moral basis to equipoise to justify randomisation should be used, focussed on expected positive benefits and personal autonomy.

Dedicated Data Collection Teams: Establishing dedicated data collection teams within intervention sites is paramount to improve recruitment, follow-up rates and data quality. These teams should be flexible and able to adapt to the ad hoc nature of participant engagement. For this population, regular methods of recruitment and follow-up will likely not work. The issues around delays with CRN contracts were due to not having worked in this setting or with this population previously. Unlike in other settings where CRNs or research teams can be placed on any study, this is not the case for people who are in prison or have left prison. Dedicated teams need to be formed who are comfortable working in prison settings as well as with people leaving prison.

Contextual Factors: HMPPS is currently facing unprecedented challenges which need to be addressed. The staffing issues within probation and prison settings need to be resolved. Until

DRAFT For peer review only

staffing levels are up to par with need, conducting research within this setting will be challenging as staff members will not have the capacity to aid research as it will not be a priority.

Conclusion

This pilot trial did not achieve two out of the four progression criteria, and a full-scale evaluation is not currently recommended. The intervention was well perceived by intervention delivery staff but they did not believe there was uncertainty in the benefit of the intervention so were uncomfortable with random allocation. These concerns were exacerbated by the lack of support for the release of people leaving prison in the control arm. Recruitment and retention of participants were impeded by the start of the UK government's co-occurring early release of people in prison and reduction in the staffing and requirements of the probation service. Levels of missing data were low on primary and secondary outcomes.

Other information

Acknowledgements We would like to thank all CTI caseworkers and the intervention delivery organisation for working closely with us on this project along with all of our collaborators. This study made use of anonymised data held in the Secure Anonymised Information Linkage (SAIL) Databank. We would like to acknowledge all the data providers who made anonymised data available for research via the SAIL Databank. Responsibility for the interpretation of the information supplied by SAIL is the authors' alone.

Contributors: JL was the chief investigator. JL, MD, JW, RCJ, YM, PM, IMJ, SVK, SF, and IT designed the study. AW created the first draft of this manuscript. All authors made substantive contributions to the development of the manuscript, critically reviewed and gave final approval to the manuscript. NJ and AW conducted the process evaluation. AW and YM were responsible for the management of the trial. DG conducted the statistical analysis. MD and SOA developed and conducted the economic evaluation.

Funding: This research was supported by the National Institute for Health and Care Research (NIHR) Public Health Research Programme, grant number NIHR134281. The Centre for Trials Research, Cardiff University receives infrastructure funding from Health and Care Research Wales. JW is supported by the Centre for Development, Evaluation, Complexity and Implementation in Public Health Improvement (DECIPHer), which is funded by the Welsh Government through Health and Care Research Wales. SVK also acknowledges funding from the Medical Research Council (MC_UU_00022/2 and the Scottish Government Chief Scientist Office (SPHSU17)

For peer review only - http://bmjopen.bmj.com/site/about/guidelines.xhtml

DRAFT V.04 26.11.24

Disclaimer: The views expressed are those of the authors and not necessarily those of HMPPS, the CTI delivery organisation, the NIHR or the Department of Health.

Competing interests: All authors declare no competing interests.

Data sharing statement: Following completion of the study, data will be made available upon request from the corresponding author. Individual-level data from the test data linkage are not publicly available due to data sharing agreements limiting access to the research team only. Researchers wishing to use the SAIL Databank should direct their queries to the contact section of the SAIL webpage at https://saildatabank.com/contact/

References

1. Volker B, De Cuyper R, Mollenhorst G, Dirkzwager A, van der Laan P, Nieuwbeerta P. Changes in the social networks of prisoners: A comparison of their networks before and after imprisonment. Soc Networks. 2016;47:47-58. doi: 10.1016/j.socnet.2016.04.004.
2. Fazel S, Geddes JR, Kushel M. The health of homeless people in high-income countries: descriptive epidemiology, health consequences, and clinical and policy recommendations. Lancet. 2014;384(9953):1529-40. doi: 10.1016/S0140-6736(14)61132-6.
3. Goering P, Tolomiczenko G, Sheldon T, Boydell K, Wasylenki D. Characteristics of persons who are homeless for the first time. Psychiatr Serv. 2002;53(11):1472-4. doi: 10.1176/appi.ps.53.11.1472.
4. Kushel MB, Perry S, Bangsberg D, Clark R, Moss AR. Emergency department use among the homeless and marginally housed: results from a community-based study. Am J Public Health. 2002;92(5):778-84. doi: 10.2105/AJPH.92.5.778.
5. Lewer D, Aldridge RW, Menezes D, Sawyer C, Zaninotto P, Dedicoat M, et al. Health-related quality of life and prevalence of six chronic diseases in homeless and housed people: a cross-sectional study in London and Birmingham, England. BMJ Open. 2019;9(4):e025192. doi: 10.1136/bmjopen-2018-025192.
6. Sun S, Irestig R, Burström B, Beijer U, Burström K. Health-related quality of life (EQ-5D) among homeless persons compared to a general population sample in Stockholm County, 2006. Scand J Public Health. 2012;40(2):115-25. doi: 10.1177/1403494811435493.
7. Aldridge RW, Story A, Hwang SW, Nordentoft M, Luchenski SA, Hartwell G, et al. Morbidity and mortality in homeless individuals, prisoners, sex workers, and individuals

For peer review only - http://bmjopen.bmj.com/site/about/guidelines.xhtml

with substance use disorders in high-income countries: a systematic review and meta-analysis. Lancet. 2018;391(10117):241-50. doi: 10.1016/S0140-6736(17)31869-X.

8. Hwang SW, Gogosis E, Chambers C, Dunn JR, Hoch JS, Aubry T. Health status, quality of life, residential stability, substance use, and health care utilization among adults applying to a supportive housing program. J Urban Health. 2011;88(6):1076-90. doi: 10.1007/s11524-011-9592-3.
9. Tweed EA, et al. Health of people experiencing co-occurring homelessness, imprisonment, substance use, sex work and/or severe mental illness in high-income countries: a systematic review and meta-analysis. J Epidemiol Community Health. 2021;75(10):1010-20. doi: 10.1136/jech-2020-215975.
10. Pleace N. At what cost? An estimation of the financial costs of single homelessness in the UK. London: Crisis; 2015. Available from: https://www.crisis.org.uk/media/237022/costsofhomelessness_finalweb.pdf.
11. Ponka D, et al. The effectiveness of case management interventions for the homeless, vulnerably housed and persons with lived experience: A systematic review. PLoS One. 2020;15(4):e0230896. doi: 10.1371/journal.pone.0230896.
12. Shaw J, et al. Critical time Intervention for Severely mentally ill Prisoners (CrISP): a randomised controlled trial. Health Serv Deliv Res. 2017;5(8):1-138. doi: 10.3310/hsdr05080.
13. Herman DB, Conover S, Gorroochurn P, Hinterland K, Hoepner L, Susser ES. Randomized trial of critical time intervention to prevent homelessness after hospital discharge. Psychiatr Serv. 2011;62(7):713-9. doi: 10.1176/appi.ps.62.7.713.
14. Kasprow WJ, Rosenheck RA. Outcomes of critical time intervention case management of homeless veterans after psychiatric hospitalization. Psychiatr Serv. 2007;58(7):929-35. doi: 10.1176/appi.ps.58.7.929.
15. Lako DAM, et al. The effectiveness of critical time intervention for abused women leaving women's shelters: a randomized controlled trial. Int J Public Health. 2018;63(4):513-23. doi: 10.1007/s00038-017-1067-1.
16. de Vet R, Beijersbergen MD, Jonker IE, Lako DAM, van Hemert AM, Herman DB, Wolf JRL. Critical time intervention for homeless people making the transition to community living: a randomized controlled trial. Am J Community Psychol. 2017;60(1-2):175-86. doi: 10.1002/ajcp.12150.

For peer review only - http://bmjopen.bmj.com/site/about/guidelines.xhtml

17. Jones K, Colson PW, Holter MC, Lin S, Valencia E, Susser E, Wyatt RJ. Cost-effectiveness of critical time intervention to reduce homelessness among persons with mental illness. Psychiatr Serv. 2003;54(6):884-90. doi: 10.1176/appi.ps.54.6.884.

18. Eldridge SM, Chan CL, Campbell MJ, Bond CM, Hopewell S, Thabane L, et al. CONSORT 2010 statement: extension to randomised pilot and feasibility trials. BMJ. 2016;355:i5239. doi: 10.1136/bmj.i5239.

19. Hoffmann TC, Glasziou PP, Boutron I, Milne R, Perera R, Moher D, et al. Better reporting of interventions: template for intervention description and replication (TIDieR) checklist and guide. BMJ. 2014;348:g1687. doi: 10.1136/bmj.g1687.

20. Herman D, Mandiberg J. Critical Time Intervention: Model Description and Implications for the Significance of Timing in Social Work Interventions. Res Soc Work Pract. 2010;20(5):502-8. doi: 10.1177/1049731509360667.

21. Ware J et al., Medical Care 34(3):p 220-233 (1996), A 12-Item Short-Form Health Survey: Construction of Scales and Preliminary Tests of Reliability and Validity, https://doi.org/10.1097/00005650-199603000-00003

22. EuroQol Research Foundation. EQ-5D-3L User Guide, 2018. Available from: https://euroqol.org/information-and-support/documentation/user-guides/

23. Keeley T, Al-Janabi H, Lorgelly P, Coast J. A Qualitative Assessment of the Content Validity of the ICECAP-A and EQ-5D-5L and Their Appropriateness for Use in Health Research. PLoS One. 2013;8(12):e85287. doi: 10.1371/journal.pone.0085287.

24. Khadjesari Z, White IR, McCambridge J, Marston L, Wallace P, Godfrey C, Murray E. Validation of the AUDIT-C in adults seeking help with their drinking online. Addict Sci Clin Pract. 2017;12(2):2. doi: 10.1186/s13722-016-0066-5.

25. StataCorp. Stata Statistical Software: Release 18. College Station, TX: StataCorp LLC; 2023.

26. Braun V, Clarke V. Thematic Analysis: A Practical Guide. SAGE Publications; 2022.

27. Lumivero. NVivo (Version 12). Lumivero; 2017. Available from: https://www.lumivero.com

28. Ministry of Justice. Transparency Data End of Custody Supervised Licence (ECSL) data, England and Wales. 2024. Available from: https://assets.publishing.service.gov.uk/media/6690004049b9c0597fdafb6c/2024-07-11_ECSL_Transparency_data.pdf

29. Manuel JI, Nizza M, Herman DB, Conover S, Esquivel L, Yuan Y, Susser E. Supporting vulnerable people during challenging transitions: a systematic review of Critical Time

For peer review

For peer review only - http://bmjopen.bmj.com/site/about/guidelines.xhtml

Intervention. Adm Policy Ment Health. 2023;50(1):100-113. doi: 10.1007/s10488-022-01224-z.

30. HMPPS. A draft target operating Model for the Future of Probation Services in England and Wales. 2020. Available from: https://assets.publishing.service.gov.uk/government/uploads/system/uploads/attachment_data/file/873093/A_Draft_Target_Operating_Model_for_the_Future_of_Probation_Services_in_England_and_Wales__in_English_.pdf

31. Cracknell M. The resettlement net: 'revolving door' imprisonment and carceral (re) circulation. Punishm Soc. 2023;25(1):223-40.

32. Inside Time. Probation Reset' stops supervision of ex-prisoners. 2024. Available from: https://insidetime.org/newsround/probation-reset-stops-supervision-of-ex-prisoners/

33. Ministry of Justice. Offender management statistics quarterly: October to December 2023 and annual 2023. 2024. Available from: https://www.gov.uk/government/statistics/offender-management-statistics-quarterly-october-to-december-2023/offender-management-statistics-quarterly-october-to-december-2023-and-annual-2023

34. Fries JF, Krishnan E. Equipoise, design bias, and randomized controlled trials: the elusive ethics of new drug development. Arthritis Res Ther. 2004;6(3):R250-5. doi: 10.1186/ar1170.

DRAFT

w only

For peer review only - http://bmjopen.bmj.com/site/about/guidelines.xhtml

DRAFT V.04 26.11.24

Tables and Figures

FIGURE 1. Housing-led CTI logic model

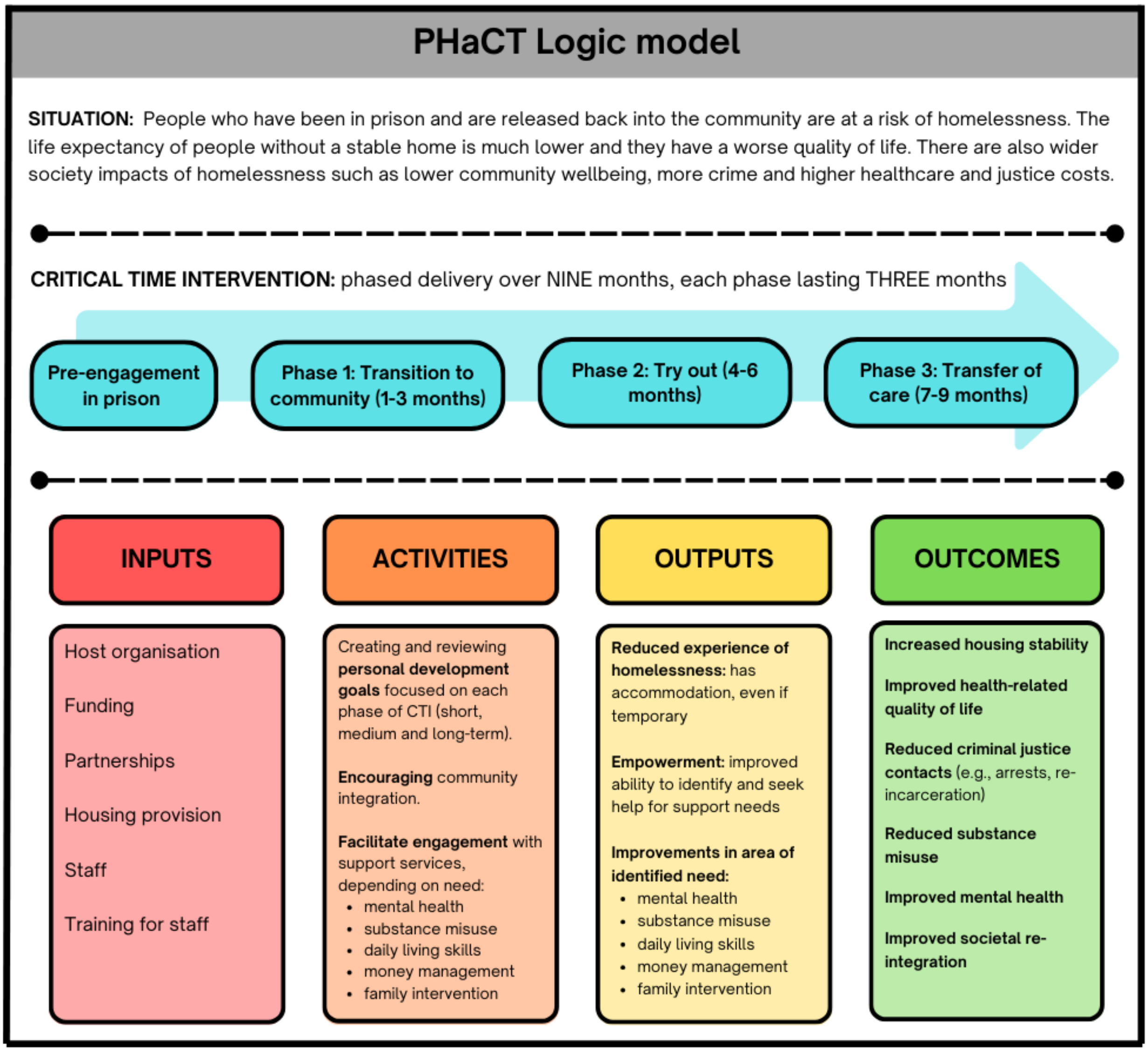

Draft only

For peer review only - http://bmjopen.bmj.com/site/about/guidelines.xhtml

DRAFT V.04 26.11.24

FIGURE 2. PHaCT CONSORT diagram

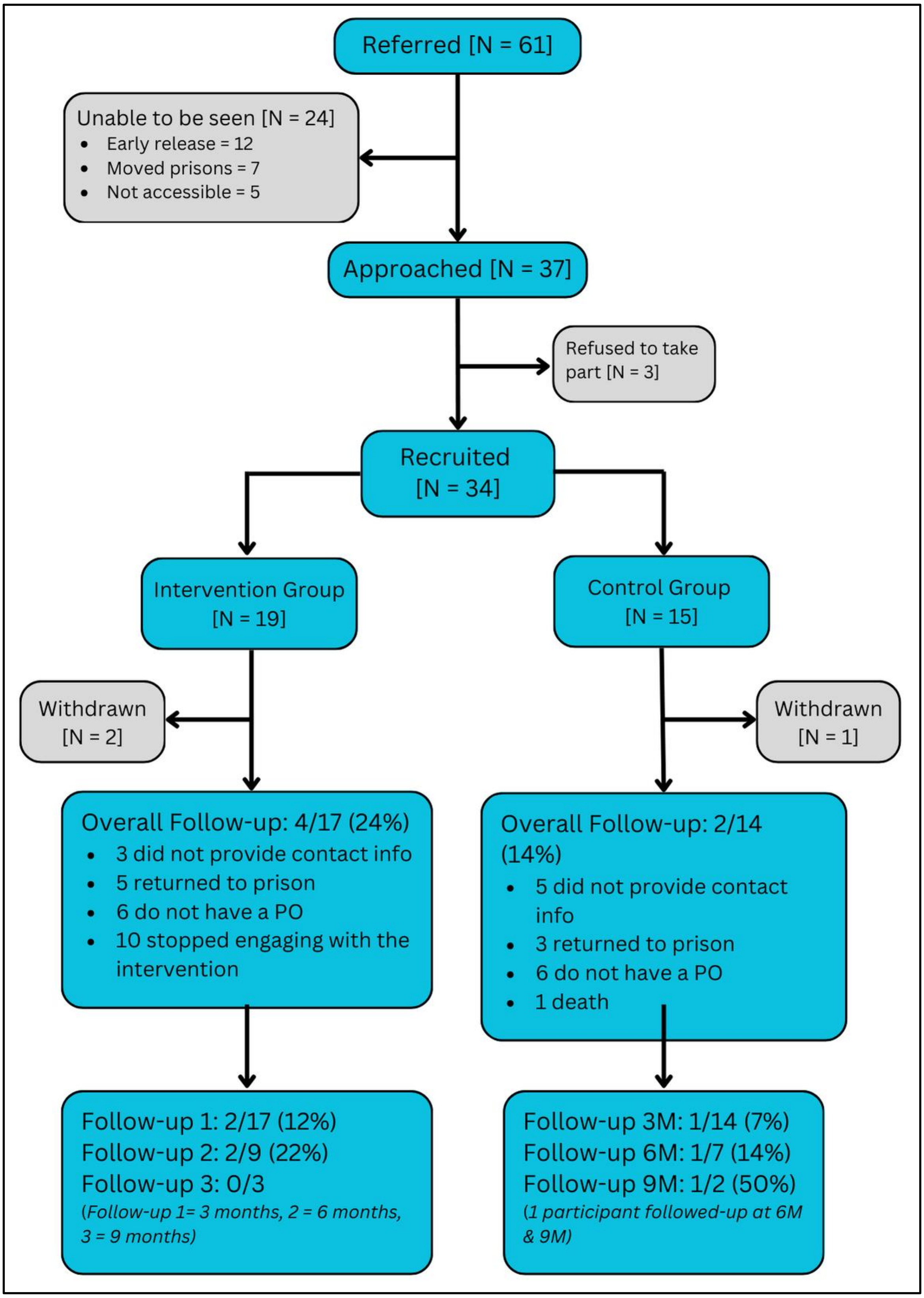

*The denominator in the fractions concerning the follow-up at 3-, 6- and 9 months reflects the number of people who had the opportunity to be followed up in the lifetime of the study.

For peer review only - http://bmjopen.bmj.com/site/about/guidelines.xhtml

DRAFT V.04 26.11.24

973 ***Table 2.*** Descriptive table based on the data collected at baseline for all participants.

	n (%)		
	**Overall sample**	**Intervention**	**Control**
Overall number	**34 (100.0)**	**19 (55.9)**	**15 (44.1)**
Age in years, mean (SD)	38.05 (11.73)	38.68 (13.65)	37.27 (9.14)
Male sex	34 (100.0)	19 (100.0)	15 (100.0)
Ethnicity			
White	31 (91.28)	17 (89.5)	14 (93.3)
Mixed or multiple ethnic groups	1 (2.94)	0 (0.0)	1 (6.7)
Caribbean or Black	1 (2.94)	1 (5.3)	0 (0.0)
Any other ethnic group (Bangladeshi)	1 (2.94)	1 (5.3)	0 (0.0)
Smoking status			
Never/Ex-smoker	8 (23.53)	5 (26.3)	3 (20.0)
Current smoker (not every day/every day)	26 (76.47)	14 (73.7)	12 (80.0)
In the 12 months before entering prison did you use illegal drugs?			
No	3 (8.82)	3 (15.8)	0 (0.0)
Yes	31 (91.18)	16 (84.2)	15 (100.0)
Education (could have more than one, selected the highest)			
No formal education	12 (35.29)	5 (26.3)	7 (46.7)
GCSE, GCE or O-level	8 (23.53)	5 (26.3)	3 (20.0)
Vocational training (e.g., Apprenticeship, City & Guilds)	12 (35.29)	9 (47.4)	3 (20.0)
Higher education	1 (2.94)	0 (0.0)	1 (6.7)
Do not know	1 (2.94)	0 (0.0)	1 (6.7)
Immediately before entering prison, were you in settled accommodation (this is medium to long-term accommodation such as social housing or a private rented or owner-occupied home)?			
No	25 (73.53)	12 (63.2)	13 (86.7)
Yes	9 (26.47)	7 (36.8)	2 (13.3)
If No to the previous question (n=25)			
Hostel	7 (28.00)	3 (25.0)	4 (30.8)
Emergency accommodation (B&B or night shelter)	3 (12.00)	1 (8.3)	2 (15.4)
A caravan or a squat	1 (4.00)	1 (8.3)	0 (0.0)
Temporarily at a friend's or family's house –on an informal basis (sofa surfing)	7 (28.00)	2 (16.7)	5 (38.5)
Rough sleeping, on transport or in a transport hub (bus stop or train station), in a tent or car	2 (8.00)	0 (0.0)	2 (15.4)
Other	5 (20.00)	5 (41.7)	0 (0.0)
Times in life being homeless (Median, IQR)	3. 0 (2 to 4); n=29	2.0 (1 to 2); n=17	3.5 (3 to 5), n=12
Once	7 (20.59)	6 (31.6)	1 (6.7)
2-9 times	19 (55.88)	10 (52.6)	9 (60.0)
10 or more	3 (8.82)	1 (5.3)	2 (13.3)
Do not know	3 (8.82)	2 (10.5)	1 (6.7)
Missing	2 (5.88)	0 (0.0)	2 (13.3)
AUDIT-C* 12 months prior to prison (Median IQR)	5.5 (0 to 12)	3 (0 to 12)	9 (0 to 12)

For peer review only - http://bmjopen.bmj.com/site/about/guidelines.xhtml

Visited a GP in prison			
No	15 (44.12)	9 (47.4)	6 (40.0)
Yes	19 (55.88)	10 (52.6)	9 (60.0)
Visited a nurse in prison			
No	10 (29.41)	5 (26.3)	5 (33.3)
Yes	24 (70.59)	14 (73.7)	10 (66.7)
Have you had a hospital admission while in prison?			
No	29 (85.29)	15 (78.9)	14 (93.3)
Yes	5 (14.71)	4 (21.1)	1 (6.7)
Have used any substance use services (e.g. alcohol or drug treatment or support)?			
No	15 (44.12)	12 (63.2)	3 (20.0)
Yes	19 (55.88)	7 (36.8)	12 (80.0)
Have you had any other healthcare contact/contact with other healthcare workers?			
No	26 (76.47)	12 (63.2)	14 (93.3)
Yes	8 (23.53)	7 (36.8)	1 (6.7)
Do you currently have any supportive relationships?			
No supportive relationships	10 (29.41)	8 (42.1)	2 (13.3)
Supportive relationships with friends	2 (5.88)	1 (5.3)	1 (6.7)
Supportive relationships with family	6 (17.65)	2 (10.5)	4 (26.7)
Supportive relationships with family and friends	16 (47.06)	8 (42.1)	8 (53.3)
ICECAP_A overall score (median, IQR)	0.672 (0.373 to 0.888)	0.698 (0.373 to 0.888)	0.589 (0.36 to 0.898)

*AUDIT-C has three questions, each scored from 0 to 4 resulting in a total score on a scale of 0 to 12 (scores of 0 reflect no alcohol use). In men, a score of 4 or more is considered positive. The higher the AUDIT-C score, the more likely it is that the participant's drinking is affecting health and safety.)

DRAFT

For peer review only

Supplementary Material

Health Economics results

1. Intervention Costs

We liaised with the CTI team, using and adapting data collection systems used by Crisis to capture the cost of delivering CTI. We captured a broad set of intervention cost components, including: staff costs (i.e. time the CTI staff spends with the participant or communicating with an external agency, such as addiction service) , housing costs (i.e. cost of paying rent or purchasing household equipment), travel cost, administrative costs (i.e. time spent by the CTI staff to review and discuss cases) and miscellaneous costs (i.e. any other costs recorded that does not fall under any of the categories above, e.g. costs for clothing).

Table 1 shows the average and total cost of the CTI intervention, by intervention phase. Of the 17 participants randomized to the CTI intervention, 13 completed the pre-engagement phase and phase 1, 9 progressed through phase 2 and only 2 participants completed all the intervention stages. The average cost per participant is £165.5 for the pre-engagement phase only; £194.5 for phase 1, £75.9 for Phase 2, £157.3 for Phase 3. The average cost for those who progress through all the 4 phases of was £674.6.

Table 1 Mean and Median Intervention Cost per Participant by Phases

Phase (n)	**Pre-engagement (17)**	**Phase 1 (13)**	**Pre-engagement+ Phase 1 (13)**	**Phase 2 (9)**	**Pre-engagement to Phase 2 (9)**	**Phase 3 (2)**	**Pre-engagement to Phase 3 (2)**
Mean	£165.5	£194.5	£248.6	£75.9	£355.1	£157.3	£674.6
Median	£74.7	£198.4	£263.7	£37.2	£311.5	£157.3	£674.6

- n= the number of participants who were enrolled in phase

Table 2 shows the total cost of the CTI intervention, by intervention phase and cost category. The cost of staff delivering the intervention represents the main cost component, accounting for 46.3% of total overall costs.

These estimates need to be taken with caution. First, descriptive statistics are based on a very small number of observations, overall and across CTI phases. Delays in recruitment have led to participants not completing all the four phases of the CTI intervention. Descriptive statistics are thus based on a different number of observations, with only 2 participants completing all the CTI intervention phases (from pre-engagement to phase 3). Also, while we initially aimed to collect travel costs as both the actual cost sustained to travel (retrieved from travel claims) and the opportunity costs (i.e. time spent by staff travelling, multiplied by the hourly staff wage), it was not possible to collect the staff opportunity cost of travel to prison. Also, housing costs were only recorded for one participant.

Table 2 Total Intervention Cost by Cost categories and phases

Phases / ***Cost categories***	**Pre-engagement**	**Phase 1**	**Phase 2**	**Phase 3**	**All phases**
Staff Cost	£775.1	£1,585.3	£380.1	£201.3	£2,941.8
% Staff cost	27.5%	62.6%	54.9%	63.9%	46.3%
Travel Cost	£96.8	£147.6	£9.2	£17.8	£271.4
%Travel Cost	3.4%	5.8%	1.3%	5.7%	4.3%

For peer review only

Miscellaneous Cost	£3.6	£87.5	£0.0	£10.0	£101.1
% Miscellaneous	0.1%	3.5%	0.0%	3.2%	1.6%
Housing Cost	£1,467.9	£0.0	£0.0	£0.0	£1,467.9
%Housing Cost	52.1%	0.0%	0.0%	0.0%	23.1%
Review/Admin Cost	£475.8	£710.2	£302.7	£85.5	£1,574.2
% Review Admin Cost	16.9%	28.1%	43.7%	27.2%	24.8%
Total Cost	£2,819.2	£2,530.5	£692.1	£314.6	£6,356.4

2. EQ-5D and ICECAP-A descriptive statistics

Table 3 shows average EQ-5D-3L and ICECAP-A scores at baseline for the 34 PHaCT participants. The average EQ-5D-3L score of PHaCT participants at baseline is 0.57 (SD=0.42), which is lower than the population norm[1]. The average ICECAP-A score of PHaCT participants at baseline is 0.62 (SD=0.29); this is similar to a previous study, considering a prisoner population[2].

Table 4 summarizes EQ-5D and ICECAP-A scores by homelessness status and use of drugs and tobacco before entering prison. As expected, drug and tobacco users have a lower quality of life and capability score than non-users, while the association between living in a settled accommodation before entering prison and wellbeing and capability score seems to go in the opposite direction.

Table 3

**EQ-5D-3L and ICECAP-A, average value at baseline**			
	N	Mean	SD
**EQ-5D-3L index**	34	**0.577**	0.420
**ICECAP-A index**	34	**0.615**	0.285

Table 4

**EQ-5D index and ICECAP-A index by key categories**						
**Lived in settled accommodation before entering prison**						
	**Yes**			**No**		
	N	Mean	SD	N	Mean	SD
**EQ-5D index**	9	**0.492**	0.484	25	**0.608**	0.401
**ICECAP-A index**	9	**0.597**	0.374	25	**0.621**	0.254
**Drug and tobacco use before entering prison**						
	**Yes**			**No**		
	N	Mean	SD	N	Mean	SD
**EQ-5D index**	31	**0.565**	0.434	3	**0.703**	0.251
**ICECAP-A index**	31	**0.603**	0.287	3	**0.733**	0.279

When considering EQ-5D-3L single dimension, we found that the percentage of PHaCT participants reporting severe or moderate problems is lower to the general population across all dimensions. This is especially true for anxiety and depression, where 47% and 27% of PHaCT participants report

For peer review only

experiencing moderate and severe depression and anxiety (vs. respectively, 19% and 1.8% in the general population).

3. Resource use at baseline

Table 5 shows descriptive statistics on healthcare resource use in prison (visits to the GP, nurse, substance use services and hospital admission).

Table 5

Visited GP in prison			
	N	%	**Number of visits (mean, SD)**
Yes	19	55.88	4.9 (8.1)
No	15	44.12	
**Visited nurse in prison**			
	N	%	**Number of visits (mean, SD)**
Yes	24	70.59	10.2 (20.1)
No	10	29.41	
**Hospital admission**			
	N	%	**Number of hospital admissions (mean, SD)**
Yes	5	14.71	1.6 (0.89)
No	29	85.29	
**Substance use services**			
	N	%	**Number of visits (mean, SD)**
Yes	19	55.88	2.2 (26)
No	15	44.12	
**Other**			
	N	%	**Number of visits (mean, SD)**
Yes	8	23.53	2.8 (3.2)
No	26	76.47	

1. Kind P, Hardman G, Macran S. UK population norms for EQ-5D, 1999.
2. Hunter RM, Anderson R, Kirkpatrick T, et al. Economic evaluation of a complex intervention (Engager) for prisoners with common mental health problems, near to and after release: a cost-utility and cost-consequences analysis. The European Journal of Health Economics 2022:1-18

A pilot feasibility randomised controlled trial of a Critical Time Intervention for people leaving prison at risk of homelessness in England and Wales [PHaCT trial]

Journal:	*BMJ Open*
Manuscript ID	bmjopen-2024-097753.R1
Article Type:	Original research
Date Submitted by the Author:	22-Oct-2025
Complete List of Authors:	Williams, Adam; Cardiff University<br>Jacob, Nina; Cardiff University, Centre for Trials Research<br>Grozeva, Detelina; Cardiff University<br>Lloyd, Barry; Cardiff University<br>Moriarty, Yvonne; Cardiff University, Centre for Trials Research<br>Deidda, Manuela; University of Glasgow, Health Economics and Health Technology assessment<br>Achiaw, Samuel Owusu; University of Glasgow<br>Thomas, Ian; Cardiff University<br>Lewis, Kelly; Cardiff University<br>Cannings-John, Rebecca; Cardiff University, Centre for Trials Research<br>Madoc-jones, Iolo; Wrexham University<br>Fitzpatrick, Suzanne; Heriot-Watt University<br>Katikireddi, Srinivasa; University of Glasgow, MRC/CSO Social & Public Health Sciences Unit<br>Mackie, Peter; Cardiff University, School of Geography and Planning<br>White, James; Cardiff University, Centre for Trials Research<br>Lewsey, Jim; University of Glasgow
**Primary Subject Heading**:	Public health
Secondary Subject Heading:	Legal and forensic medicine
Keywords:	Vulnerable Populations, Randomized Controlled Trial, Quality of Life, Prisons, Feasibility Studies

SCHOLARONE™
Manuscripts

I, the Submitting Author has the right to grant and does grant on behalf of all authors of the Work (as defined in the below author licence), an exclusive licence and/or a non-exclusive licence for contributions from authors who are: i) UK Crown employees; ii) where BMJ has agreed a CC-BY licence shall apply, and/or iii) in accordance with the terms applicable for US Federal Government officers or employees acting as part of their official duties; on a worldwide, perpetual, irrevocable, royalty-free basis to BMJ Publishing Group Ltd ("BMJ") its licensees and where the relevant Journal is co-owned by BMJ to the co-owners of the Journal, to publish the Work in this journal and any other BMJ products and to exploit all rights, as set out in our licence.

The Submitting Author accepts and understands that any supply made under these terms is made by BMJ to the Submitting Author unless you are acting as an employee on behalf of your employer or a postgraduate student of an affiliated institution which is paying any applicable article publishing charge ("APC") for Open Access articles. Where the Submitting Author wishes to make the Work available on an Open Access basis (and intends to pay the relevant APC), the terms of reuse of such Open Access shall be governed by a Creative Commons licence – details of these licences and which Creative Commons licence will apply to this Work are set out in our licence referred to above.

Other than as permitted in any relevant BMJ Author's Self Archiving Policies, I confirm this Work has not been accepted for publication elsewhere, is not being considered for publication elsewhere and does not duplicate material already published. I confirm all authors consent to publication of this Work and authorise the granting of this licence.

For peer review only

V1.1 17OCT2025

A pilot feasibility randomised controlled trial of a Critical Time Intervention for people leaving prison at risk of homelessness in England and Wales [PHaCT trial]

Short Title: PHaCT: a pilot RCT

Authors:

Adam DN Williams 1* ORCID ID: 0000-0002-4825-8997
Nina Jacobs 1
Detelina Grozeva 1 ORCID ID: 0000-0003-3239-8415
Barry Lloyd 1 ORCID ID: 0000-0003-4763-1726
Yvonne Moriarty 1 ORCID ID: 0000-0002-7608-4699
Manuela Deidda 2 ORCID ID: 0000-0002-0921-6970
Samuel Owusu Achiaw 2 ORCID ID: 0009-0000-5711-7240
Ian Thomas 3 ORCID ID: 0000-0001-9808-0202
Kelly Lewis 1
Rebecca Cannings-John 1 ORCID ID: 0000-0001-5235-6517
Iolo Madoc-jones 4 ORCID ID: 0000-0002-3501-9639
Suzanne Fitzpatrick 5
Srinivasa Vittal Katikireddi 6 ORCID ID: 0000-0001-6593-9092
Peter Mackie 7, ORCID ID: 0000-0003-1494-0864
James White 1,8 ORCID ID: 0000-0001-8371-8453
Jim Lewsey 2 ORCID ID: 0000-0002-3811-8165

Affiliations:

1 – Centre for Trials Research, School of Medicine, College of Biomedical & Life Sciences, Cardiff University, Wales, UK (United Kingdom).
2 – Health Economics and Health Technology Assessment, School of Health and Wellbeing, University of Glasgow, Glasgow, UK
3 – Administrative Data Research Wales/ Wales Institute of Social and Economic Research and Data, School of Social Sciences, Cardiff University, Wales, UK (United Kingdom).
4 – Cyfiawnder The Social Inclusion Research Centre, Wrexham University, Wales, UK
5 - Institute for Social Policy, Housing and Equalities Research (I-SPHERE), Heriot-Watt University
6 - MRC/CSO Social & Public Health Sciences Unit, School of Health and Wellbeing, University of Glasgow, Glasgow, UK
7 - School of Geography and Planning, Cardiff University, UK
8 - DECIPHer, School of Social Sciences, Cardiff University, UK

Corresponding author: *Adam D N Williams PhD. Centre for Trials Research, School of Medicine, Cardiff University, Wales, UK. E-mail: WilliamsAD7@cardiff.ac.uk.

Abstract

For peer review only - http://bmjopen.bmj.com/site/about/guidelines.xhtml

V1.1 17OCT2025

Objective To determine whether a full-scale randomised control trial (RCT) assessing the efficacy and cost-effectiveness of a housing led Critical Time Intervention (CTI) is feasible and acceptable.

Design Pilot parallel two-arm individual level RCT, including process evaluation and embedded exploratory health economic evaluation.

Setting Four male prisons across England and Wales, UK.

Participants Men leaving prison at risk of homelessness and intervention delivery staff.

Intervention CTI has four components: (1) Pre-engagement phase: assessing client's needs and implementing a plan pre-discharge, (2) Transition to community: forming relationships and goal setting, (3) Try out: encouraging problem-solving and managing practical issues, (4) Transfer of care: developing long-term goals and transferring responsibilities to community providers.

Outcome measures Progression criteria: recruitment, retention, acceptability of the processes (CTI and trial method) and fidelity of intervention delivery. We also assessed the completeness of primary, secondary and exploratory outcome measures and estimated intervention costs.

Results The recruitment progression criterion was met, with 92% (34/37) of approached individuals consenting to participate (target: 50%). However, the overall recruitment target of 80 was not achieved, and retention was low, only 18% (6/34) provided follow-up data, well below the 60% threshold. Retention was hindered by systemic challenges, including changes to prison release policies and reduced probation support. While the CTI model was acceptable to staff and service users, the trial design, particularly randomisation, was not. Intervention fidelity met the progression criteria. Baseline data collection for health economics and resource use was feasible, and intervention costs were estimated.

Conclusions This pilot trial identified significant challenges to conducting a full-scale RCT of CTI in this context, particularly around retention, trial acceptability, and systemic instability. While CTI remains a promising model, a traditional RCT design may not be viable in this setting without substantial structural and ethical adaptations.

Trial Registration: ISRCTN46969988

Funding: National Institute for Health and Care Research (NIHR), grant number NIHR134281.

For peer review only

Keywords: Homelessness prevention, prison discharge, randomised controlled trial, housing-led critical time intervention (CTI)

Strengths and limitations:

1. The study used a pilot parallel two-arm RCT with embedded process and exploratory economic evaluations, offering a robust framework for feasibility assessment.
2. Ethical and practical concerns around randomisation affected trial implementation and acceptability.
3. Logistical challenges, including delays in approvals and difficulties maintaining contact post-release, impacted trial delivery.
4. External factors, such as changes in prison resettlement policies and staffing shortages, posed major barriers to recruitment and retention.

MAIN TEXT [3953 words]

INTRODUCTION

For people leaving prison, re-integrating into the community can be a difficult process. The absence of suitable affordable housing and a stable support network, in the context that many may have complex needs linked to substance use and mental ill-health, makes people leaving prison more susceptible to homelessness [1]. The absence of stable accommodation can then have further repercussions, people experiencing homelessness are at a higher risk than the general population of infectious and noncommunicable diseases, mental health problems, alcohol and substance use, have higher rates of hospital admissions and report lower levels of wellbeing and health-related quality of life [1-4]. Health outcomes are substantially worse amongst people with a history of both imprisonment and homelessness than each in isolation [5]. The detrimental consequences of homelessness following imprisonment can lead to a huge societal burden in terms of healthcare and societal costs [6].

Critical Time Interventions (CTIs) are structured, time-limited case management approaches designed to support individuals during periods of transition, such as release from prison, by enhancing engagement with treatment and community services through problem-solving and continuity of care [7]. Core components include small caseloads, active outreach, personalised case management, psychosocial skill-building, and motivational support, delivered through a phased model that tapers in intensity over time [7-8]. A UK multisite randomised control trial (RCT) demonstrated that CTI could be feasibly and acceptably implemented in prison settings, showing improved service engagement and providing valuable economic evaluation data [8]. While international evidence suggests CTI may improve housing stability among people experiencing or at risk of homelessness, its impact on health outcomes and quality of life remains limited [8-12]. Gaps remain, particularly the limited UK-based RCTs

For peer review only - http://bmjopen.bmj.com/site/about/guidelines.xhtml

For peer review only

among prison-leavers facing housing instability, highlighting the need for further research in this area. The overall aim was to conduct a pilot RCT to determine whether a future full-scale RCT assessing effectiveness and cost-effectiveness of a housing-led CTI is warranted.

METHODS

The full protocol is available in the supplemental material.

Progression criteria

We aimed to recruit 20 participants from each of the four prisons in which the intervention was being delivered, totalling 80 participants. The sample size of 80 participants at the end of the pilot would allow binary outcomes to be estimated. A traffic light system was in place to judge feasibility:

1) Recruitment: 50% of those approached agree to participate.
2) Retention: 60% of those recruited, in both intervention and control groups, are retained at final follow-up.
3) The process evaluation provides evidence that the process is acceptable for participants and staff delivering the intervention.
4) The intervention is implemented with fidelity in all settings.

Trial design

This pilot study undertook a parallel two-arm, individual level RCT of a pre-existing CTI intervention, including a process evaluation and embedded exploratory health economic evaluation.

Setting

Participants were intended to be recruited from four prisons across England and Wales, housing adult males. The locations were predetermined by where the intervention was already being delivered by the intervention provider (CTI team).During recruitment the protocol was updated to include an additional site, but this site did not progress to recruitment. At completion, only three of the four prisons provided participants.

Participants

Inclusion criteria:

- Men leaving prison at risk of homelessness
- Aged over 18 years
- Released into the local authority areas that CTI is being delivered and have a local connection (e.g., lived there previously; close relatives living in these areas)
- Recourse to public funds
- Have experienced prison and homelessness at least once.

For peer review only - http://bmjopen.bmj.com/site/about/guidelines.xhtml

- Have mental health or substance use support needs

Exclusion criteria:

- Under the Multi-Agency Public Protection panel 3 (MAPPA 3)
- In receipt of Housing First (i.e., support needs too high/complex to benefit from CTI)

Randomisation and blinding

Randomisation was completed after baseline data collection. Participants were randomised on a 1:1 ratio and stratified by site, using random block sizes. Randomisation was completed using concealed paper envelopes. Envelopes contained the arm allocation and participant identification number (PID) number. Boxes and envelopes were clearly labelled with the prison name and sequence number. Training was provided to recruitment staff to outline the randomisation method. Participants were randomised from October 2023 to July 2024.

Intervention

Figure 1 shows the logic model for Critical Time Intervention (CTI), a psychosocial approach supporting individuals transitioning from institutions to the community. This UK adaptation follows a "housing-led" model, prioritising rapid access to stable housing as a foundation for addressing broader health and social needs.

CTI strengthens long-term connections to services, family, and peers through time-limited emotional and practical support. Clients co-develop personal plans with short-, medium-, and long-term goals to build self-management skills, empowerment, and improve wellbeing. Stable housing is central, reducing the stress and instability linked to homelessness.

The intervention begins before discharge, with an assessment panel identifying individuals at risk of homelessness. Caseworkers initiate engagement, assess needs, and co-create a tailored transition plan. Post-release, clients are supported into tenancies and progress through three phases:

- **Phase 1: Transition to Community (3 months)** – Intensive support, weekly visits, goal-setting, and crisis resolution.
- **Phase 2: Try Out (3 months)** – Reduced contact; clients practice independence with support adjusted as needed.
- **Phase 3: Transfer of Care (3 months)** – Caseworker steps back, finalising handover to community supports and caregivers.

CTI was delivered by caseworkers from a UK charity specialising in housing support for prison leavers. Support was provided in prisons, offices, community settings, and residences across England and Wales. The intervention was funded by a UK charity.

For peer review only - http://bmjopen.bmj.com/site/about/guidelines.xhtml

Ethics and consent

The study received NHS ethics approval from Wales REC 3 (22/WA/0347), NRC approval (the National Research Council governs research within prison and probation settings), and permissions from each prison Governor and Probation Regional Manager. The study was registered in ISRCTN46969988. Access to routine data via the Secure Anonymised Information Linkage (SAIL) Databank was granted by the SAIL Information Governance Review Panel (SAIL ID:1365).

Potential participants were referred to the CTI team for eligibility screening by the Prison, Community Offender Management Team or a self-referral. The CTI team would engage the individual and complete their referral assessment. Those deemed eligible were provided with a brief verbal explanation of the trial with the support of a summary information sheet and asked if they were interested in taking part. For those interested, prison visits were organised for the study to be explained, information sheets and consent forms to be signed and, following informed consent, baseline data collection occurred.

For peer review only

Data collection

Trial data collection

The CTI team identified eligible individuals based at prisons where they delivered the intervention. At recruitment, baseline data collection occurred in the form of paper surveys in the prisons where participants were located.

Follow-up was scheduled at 3, 6, 9, and 12 months post-randomisation, with a 2-week flexible window around the scheduled timepoint to accommodate participant availability and logistical constraints. Follow-ups were conducted in probation offices, intervention team offices, or via telephone. Probation services were engaged to help track participants post-release and facilitate follow-up contacts, although their involvement varied by site and availability.

The intention was to use Clinical Research Nurses (CRNs, registered nurses specialised in managing and conducting clinical trials and research studies) to complete recruitment, baseline data and follow-up data collection for England sites with the local R&D delivery team (the Welsh equivalent of CRN) being used for Wales. The use of CRNs is standard in health research and was chosen for this study to reflect the approach that would be adopted in a full-scale UK-wide trial. In Wales, recruitment, baseline data collection and follow-up was completed by local R&D delivery team while recruitment and data collection in England was completed by the research team due to logistical challenges with the CRN teams.

Process evaluation

Semi-structured interviews were to be conducted with participants and CTI caseworkers from both England and Wales. The intention was to conduct 24 interviews with participants from

For peer review only - http://bmjopen.bmj.com/site/about/guidelines.xhtml

the intervention group (12 participants to be interviewed at phase 1 and 3 of the intervention) and 16 interviews with participants from the control group.

Twenty-four observations were planned with service users of the intervention, twelve with participants in the RCT control group and twelve with service users of the CTI model who started receiving support before the study randomisation began. These ranged across the phases of the intervention. A full description of the process evaluation is presented in the following paper: [ADD LINK HERE]

Health economic evaluation

Cost logs from the intervention delivery team examined time and resources spent implementing the intervention.

Outcome measures

Feasibility outcomes

These criteria sought to assess the number who agreed to participate, the retention at the end of the study, the acceptability of the delivery of the intervention and RCT design and fidelity of delivery, with thresholds set according to a traffic light system.

Trial outcome measures

The indicative primary outcome for the trial was health related quality of life, measured using the validated SF-12 [13]. This was chosen for being a recognised and validated outcome in public health and social care research, making it a meaningful and policy-relevant indicator for assessing the broader benefits of CTI beyond specific behavioural or clinical endpoints. The secondary outcomes are displayed in Table 1 and include housing stability, capability wellbeing, hazardous alcohol consumption and substance use and resource use and mortality.

The exploratory outcomes assessed whether participants consented to link to routinely collected healthcare and criminal justice data. Of those who consented, we also sought to explore the proportion of people who could be successfully linked to their healthcare records, via SAIL/NHS England, and criminal justice records, via the Ministry of Justice Data Lab, by applying to use these data linkage services. Attempting 'test linkages' sought to inform the potential use of data linkage to provide additional secondary outcomes, such as re-incarceration, re-conviction, education, training and employment, and welfare benefits receipt.

Table 1. Indicative and exploratory outcome measures

Outcome	Measurement	Timepoint
Indicative primary and secondary outcomes		

For peer review only

For peer review only - http://bmjopen.bmj.com/site/about/guidelines.xhtml

Health related quality of life (primary outcome for full-scale trial)	SF-12: Short form health survey [13]	Baseline<br>Follow-up at 3, 6, 9 and 12 months
Health related quality of life	EQ-5D: A standardised measure of health-related quality of life [14]	
Housing stability	• Type of accommodation<br>• Number of days in a stable accommodation	
Substance use and hazardous alcohol consumption	AUDIT-C: Alcohol Use Disorders Identification Test Consumption [15]	
Mortality	Recording participant deaths, including cause of death	
Capability wellbeing	ICECAP-A: ICEpop CAPability measure for Adults [14]	
Resource use	Ad hoc measurement of resource use for health economic evaluation	
Exploratory outcomes		
Data linkage	Percentage of participants who consent to link their data anonymously with routine electronic data through SAIL, NHS England and Ministry of Justice Lab	Single point of data linkage
	Percentage of records successfully linked with data held in SAIL, NHS England, Ministry of Justice Data Lab to inform:<br>• Health and wellbeing<br>• Re-incarceration<br>• Crime records	

Analysis

No power calculation was performed as our aim was to evaluate acceptability of the intervention, not to estimate effectiveness. This study determined rates of recruitment, response and retention, and the distribution of the indicative primary outcome measures to inform a full-scale trial sample size calculation. Given there was capacity to deliver the intervention to 120 participants per year, we aimed to recruit 80 participants (40 per arm) over four months allowing us to estimate a recruitment rate of 50% within a 95% confidence interval (CI) ±11% (75% rate ±9.5%).

Statistical analysis

The primary analysis sought to determine whether the pre-specified progression criteria were met. The analyses were primarily descriptive, providing estimates of recruitment, response and retention rates. Recruitment, randomisation and retention of participants at follow-up were summarised in a CONSORT flow diagram (see Figure 2). We tabulated demographic characteristics of participants within settings by study arm (intervention or control) and assessment time point (baseline or follow-up) using descriptive statistics means and standard

For peer review only

For peer review only - http://bmjopen.bmj.com/site/about/guidelines.xhtml

V1.1 17OCT2025

deviations (or medians and interquartile ranges [IQR], as appropriate) for continuous outcomes, and frequencies and percentages for discrete outcomes. Analysis was performed in Stata version 18 [16].

Qualitative analysis

Qualitative data generated through semi-structured interviews were audio-recorded, transcribed and coded. Fieldnotes from observations and free-text entries in logbooks were coded using a similar system. Members of the research team analysed the data using inductive and deductive thematic analysis [17]. A coding scheme was developed by the researchers. The coding scheme evolved during analysis, with the new codes discussed and confirmed by the team, before being applied to previously coded data. NVivo 12 supported data storage and analysis[18].

Fidelity to the CTI model was measured against five fidelity items: housing-led approach, time limited and phased approach, caseloads and supervision, person centred approach/community focussed, and harm reduction/recovery orientated approach. From the interviews and observations, a score was provided: (Low=1, Medium=2, High=3) against each fidelity item with an overall score calculated.

The qualitative findings will only be briefly mentioned in relation to the feasibility criteria. A full explanation of the findings of the process evaluation is available in the associated article.

Economic evaluation

We reported descriptive statistics for health economics outcomes (EQ-5D-3L and ICECAP-A) and resource use by collection time point and intervention group. Quality of life and capability wellbeing scores were compared with reference values for the general population. The cost of delivering the CTI intervention was captured by adapting a tailored cost log to the existing data collection system used by the charity delivering the intervention.

Stakeholder engagement

An individual with lived experience of homelessness contributed to the design phase when applying for funding. The research questions were developed to align with the needs and experiences of the population at risk of homelessness.

Challenges experienced

The study experienced significant delays that impacted recruitment timelines, beginning with approval and contractual issues. Although originally planned to open within six months, in Wales recruitment began after 16 months and after 22 months in England. Delays were caused by a prolonged National Research Committee (NRC) approval process and challenges

For peer review only

For peer review only - http://bmjopen.bmj.com/site/about/guidelines.xhtml

in obtaining accurate contact details for relevant HMPPS staff. The CTI team in England also faced staff shortages, which paused intervention recruitment for an extended period.

Additional complications arose with CRN teams. Although initially assigned to manage recruitment and follow-up, unexpected site-specific contractual requirements in the prison setting delayed their involvement. For recruitment in England (which was set to recruit 60 of the 80 participants) these delays led to a protocol amendment allowing the research team to take over recruitment and follow-up. However, as the team was not locally based, this required intensive planning and clustered visits, which limited opportunities for participant engagement.

Results

In total, 34 individuals were recruited to the trial from an intended 80 participants (that is, 43% recruited from the target of 80 trial participants). Table 2 presents the recruitment figures across the study components. Fifteen individuals (44%) were randomised to the control arm and 19 (56%) to the intervention arm (see Figure 2, consort diagram). Of the trial participants, the mean age was 38.1 years (standard deviation (SD): 11.2 years, min: 19, max: 67), and 9% were from ethnic minority backgrounds, comprising Mixed or Multiple ethnicities, Bangladeshi, and Caribbean or Black. Overall, 35% (n=12) reported to not have any formal educational qualifications. Illicit drug use in the period of 12 months before entering prison was reported by 91% (n=31) of participants. Participants reported experiencing homelessness on average 3 times since the age of 16 [IQR: 2-4], with 74% (n=25) reporting they were not in settled accommodation before entering prison. Full demographic information is available in the supplemental material.

Table 2. Summary of recruitment across study.

	Wales, site 1	England, site 2
Intervention delivery team	CTI team	CTI team
Data collection team	R&D delivery team	Research team
Number of prisons providing participants	1	2
Trial participants recruited/ planned recruitment	20/20	14/60
Trial participants followed up	5	1
Number of interviews conducted (participants and staff)	4	4
Observation days	10	20
Observation locations	CTI office	CTI office, prison, probation, community settings

For peer review only - http://bmjopen.bmj.com/site/about/guidelines.xhtml

V1.1 17OCT2025

Progression criteria

Table 3 summarises the results against the progression criteria. Two of the four progression criteria were rated green (recruitment and fidelity). The process evaluation was rated amber, with retention receiving a red rating.

Table 3. Summary of results against the progression criteria

Progression Criterion	Red	Amber	Green	Actual
1. 50% of those approached agree to participate.	<40%	40-49%	≥50%	92%
2. 60% of those recruited are retained at final follow-up.	<50%	50-59%	≥60%	18%
3. The process (CTI and trial process) is acceptable for participants and staff.	Neither is accepted by participant or staff	CTI or trial process is accepted by participant or staff	Both are accepted by participant and staff	CTI = acceptable Trial = not acceptable
4. The intervention is implemented with fidelity in all settings.	Fidelity score <20	Fidelity score 20-25	Fidelity score ≥25	Overall score = 26

Recruitment

CTI caseworkers referred 61 people to the research team but 24 people were unable to be approached within the prison setting (so could not be recruited); reasons included early release (12/24, 50%), moving prison (7/24, 29%), not being accessible within the prison, i.e., held in cell for misbehaviour, attending other meetings/prison visit (5/24, 21%). Of the 37 approached, 34 (92%) agreed to take part (the 'green' threshold for this criterion was ≥50%); for the three who refused, reasons included distrust of research and experiencing mental health difficulties. While the green threshold was met based on the criteria's wording, the planned sample size of 80 participants was not achieved.

Retention

Of the 34 participants recruited, there was a death which occurred before follow-up, three withdrawals, and eight participants returned to prison, leaving 22 people to attempt follow-up. The eight participants who returned to prison could not be followed up as it was either unknown which prison they had been sent to, or it was not a prison where we had approvals to conduct research. Six of the 34 recruited participants had follow-up data for at least 1-time point (18%, the 'green' threshold was 60%), two participants were allocated to the control group and four to the intervention. At 3-months, three participants were followed up (9%, 3/34) and 6 months, three participants were followed up (9%, 3/34). One participant was followed up at 9 months (3%, 1/34). One participant was followed up for two time points; the rest were followed up at

For peer review only - http://bmjopen.bmj.com/site/about/guidelines.xhtml

V1.1 17OCT2025

a single time point. Due to delays at trial initiation, no participants achieved the 12-month follow-up point. Of the 22 participants who could be followed up, eight (8/22, 36%) did not provide any contact details (due to not having any contact information or not knowing their details from memory), those who did provide contact details (email, telephone or both) were contacted four times at each follow-up point. For many of the participants who provided a telephone number, it had been disconnected when contact was attempted (8/14, 57%).

To support follow-up, we would engage with probation officers to maintain contact with participants at follow-up, however details of probation officers was not always known. Twelve (12/34, 35%) did not have any details for their probation practitioner at baseline, 12/34 (35%) had only a name with a partial address, and 10 participants (30%) had a name and contact information for their probation practitioner. Throughout the study period the strain on the Probation Service following on from the ECSL and Probation Reset scheme resulted in many of our participants not having contact with probation staff[19].

Process evaluation

A detailed reporting of the findings from the process evaluation and fidelity assessment are provided in the associated paper. Only a summary is reported here for context.

The process evaluation aimed to assess the acceptability of the CTI model and the trial methodology, to both participants and staff delivering the intervention. In summary, the intervention's acceptability was primarily reflected through the positive feedback and success stories shared by CTI caseworkers, as well as observational data indicating high acceptance among service users. The trial design's acceptability was challenged by concerns about randomisation and equipoise, with some staff viewing randomisation as unethical due to limited support for vulnerable populations. This led to an amber rating.

Fidelity

Table 4 presents the summary of the fidelity scores across sites. Across the 5 domains, the intervention was assessed to be delivered with fidelity and scored to be green on the progression criteria.

Fidelity to CTI's housing-led, time-limited model was largely maintained, with stable housing prioritised before addressing other needs. However, limited housing availability, especially for single men leaving prison, often delayed progress, leading to occasional extensions beyond the intended nine months. Caseloads remained within or close to the recommended range, with site 1 averaging 15 and site 2 ranging from 18–28. This was considered manageable due to staff experience and phased support intensity. CTI was delivered with a strong person-centred focus, supporting service users to set goals and navigate complex systems. Harm reduction and recovery principles were embedded throughout, though unsuitable or temporary

For peer review only - http://bmjopen.bmj.com/site/about/guidelines.xhtml

housing placements often compromised recovery efforts, requiring caseworkers to intensify support and connect individuals with relevant services.

Table 4 Fidelity scores across Sites.

	Wales, Site 1	England, Site 2	Overall
Housing-led approach	2	3	5
Time limited and phased approach	2	2	4
Caseloads and supervision	3	2	5
Person centred approach/ community focussed	3	3	6
Harm reduction/ recovery orientated approach	3	3	6
Overall	**13**	**13**	**26**

NB. From the interviews and observations, a score was provided: (Low=1, Medium=2, High=3) against each fidelity item with an overall score calculated.

Indicative outcomes

A breakdown of the indicative outcomes is available in the supplemental material. The planned economic evaluation could not be conducted due to limited follow-up data. At baseline, the average EQ-5D score was 0.57 (SD=0.42) and ICECAP-A was 0.62 (SD=0.29). Most participants (73.5%) reported not being in settled accommodation before prison, with higher rates in the control group. Among six participants with follow-up data, housing situations varied, and two had returned to prison. Control group participants reported higher alcohol use prior to prison. While only two participants completed all CTI phases, missing data were minimal, supporting the feasibility of using SF-12, EQ-5D, ICECAP-A, and resource use tools at baseline. Full details are provided in the supplementary material.

Exploratory outcomes

All participants consented to data linkage to healthcare data sources, and to link to criminal justice data sources. Due to delays in recruitment, and as most of the sample were initially recruited from the Welsh study setting, test linkages using NHS England were not conducted. The intervention delivery organisation attempted to access the Justice Data Lab but were unable to use the service due to issues around the timings of data collection and not enough time had elapsed for one-year offending to have taken place following exit from the CTI intervention. Test linkages were however attempted via the SAIL Databank. As SAIL only relates to people who have been resident in Wales, people recruited from sites in England were excluded from the test linkage (14/34, 41%). In total, data relating to 17 participants at baseline were sent to SAIL for matching. Viable linkages were possible for 11 participants (65%) whose information was sent to SAIL, with the remaining 6 participants (35%) having

For peer review only - http://bmjopen.bmj.com/site/about/guidelines.xhtml

non-viable linkages, either due to no link being possible or because the probabilistic matching threshold was less than 90%.

Economic Evaluation

Due to the small number of participants completing follow-up, it was not possible to conduct the planned economic evaluation or compare outcomes and costs over time. Instead, the study presents a descriptive analysis of CTI intervention costs, which totalled £6,356, with a detailed breakdown provided in the supplementary material. Only two of the 17 participants randomised to CTI completed all intervention stages. Cost estimates should be interpreted cautiously due to limited data and missing travel cost components.

Discussion

This pilot RCT assessed the feasibility of delivering a housing-led Critical Time Intervention (CTI) for prison-leavers experiencing homelessness. Of the four pre-specified progression criteria, only two were met, suggesting that a full-scale RCT of this model, in its current form, is not feasible. While agreement to participate was high (92% of those approached consented), we did not achieve our intended sample and retention was low, with only 18% of participants being contactable for follow-up. This was largely due to contextual disruptions, including the introduction of the End of Custody Supervised Licence scheme, which accelerated release timelines and undermined recruitment and follow-up processes.

Compared to previous studies, such as Shaw et al.'s UK-based RCT of CTI [8], which demonstrated feasibility and positive outcomes in service engagement, our trial faced more significant structural and operational barriers. These included rapid policy changes, high licence recall rates, and staffing shortages in probation services, all of which contributed to instability and reduced participant engagement [19-21]. While quasi-experimental designs are a valid alternative, they are less robust than RCTs for establishing intervention effectiveness. Importantly, RCTs are feasible in prison settings, as demonstrated in previous studies [8], but they require embedded research teams and stable operational conditions. These conditions were not consistently present in this study, which followed a traditional health research model using external partners for data collection managed by a central trial team, an approach that proved less suited to the dynamic and restrictive prison environment.

A key limitation was the inability to offer incentives due to National Research Committee (NRC) restrictions. This policy affected all participants, not just those in the control group, and likely contributed to low retention. The lack of compensation, standard in general population research, raises ethical concerns around equity and fairness, particularly when working with marginalised groups. Alternative engagement strategies, such as maintaining consistent

For peer review only

For peer review only - http://bmjopen.bmj.com/site/about/guidelines.xhtml

researcher contact and offering regular check-ins, should be explored, but these alone may not be sufficient without addressing the broader ethical and structural barriers.

Despite these challenges, the study demonstrated strong staff engagement with the CTI model and high levels of consent to data linkage, suggesting that routine data could be a viable supplement for future evaluations. However, routine data is limited to “hard outcomes” (e.g. healthcare use, criminal justice involvement) and may not capture the full impact of interventions like CTI on wellbeing or quality of life.

The findings highlight the need for systemic changes to support research in prison settings. These include revising NRC policies on incentives, streamlining approvals, and investing in dedicated research teams familiar with the prison context. Future research should consider alternative trial designs that address ethical concerns around equipoise, perhaps by focusing on expected positive outcomes and personal autonomy rather than uncertainty of benefit. Additionally, the current pressures on His Majesty’s Prison and Probation Service (HMPPS), including staffing shortages and policy churn, must be addressed to create a more stable environment for research and service delivery.

In conclusion, while the CTI model remains promising, this pilot trial underscores the practical and ethical challenges of conducting RCTs in this context. Future studies should prioritise flexible, context-sensitive designs, ethical engagement strategies, and structural reforms to enable meaningful evaluation of interventions for prison-leavers experiencing homelessness.

Other information

Acknowledgements We would like to thank all CTI caseworkers and the intervention delivery organisation for working closely with us on this project along with all of our collaborators. As well as the Centre for Homelessness Impact who assisted with the development of participant facing materials and appropriate language use. This study made use of anonymised data held in the Secure Anonymised Information Linkage (SAIL) Databank. We would like to acknowledge all the data providers who made anonymised data available for research via the SAIL Databank. Responsibility for the interpretation of the information supplied by SAIL is the authors’ alone.

Contributors: JL was the chief investigator and acted as guarantor. JL, MD, JW, RCJ, YM, PM, IMJ, SVK, SF, IT designed the study. AW created the first draft of this manuscript. All authors made substantive contributions to the development of the manuscript, critically reviewed and gave final approval to the manuscript. NJ and AW conducted the process evaluation. AW and YM were responsible for the management of the trial. DG conducted the statistical analysis. MD and SOA developed and conducted the economic evaluation.

For peer review only

For peer review only - http://bmjopen.bmj.com/site/about/guidelines.xhtml

V1.1 17OCT2025

Funding: This research was supported by the National Institute for Health and Care Research (NIHR) Public Health Research Programme, grant number NIHR134281.

Disclaimer: The views expressed are those of the authors and not necessarily those of HMPPS, the CTI delivery organisation, the NIHR or the Department of Health.

Competing interests: All authors declare no competing interests.

Data sharing statement: Following completion of the study, data will be made available upon request from the corresponding author. Individual level data from the test data linkage are not publicly available due to data sharing agreements limiting access to the research team only. Researchers wishing to use the SAIL Databank should direct their queries to the contact section of the SAIL webpage at https://saildatabank.com/contact/

References

1. Fazel S, Geddes JR, Kushel M. The health of homeless people in high income countries: descriptive epidemiology, health consequences, and clinical and policy recommendations. Lancet. 2014;384(9953):1529-40. doi: 10.1016/S0140-6736(14)61132-6.
2. Goering P, Tolomiczenko G, Sheldon T, Boydell K, Wasylenki D. Characteristics of persons who are homeless for the first time. Psychiatr Serv. 2002;53(11):1472-4. doi: 10.1176/appi.ps.53.11.1472.
3. Kushel MB, Perry S, Bangsberg D, Clark R, Moss AR. Emergency department use among the homeless and marginally housed: results from a community based study. Am J Public Health. 2002;92(5):778-84. doi: 10.2105/AJPH.92.5.778.
4. Lewer D, Aldridge RW, Menezes D, Sawyer C, Zaninotto P, Dedicoat M, et al. Health-related quality of life and prevalence of six chronic diseases in homeless and housed people: a cross-sectional study in London and Birmingham, England. BMJ Open. 2019;9(4):e025192. doi: 10.1136/bmjopen-2018-025192.
5. Tweed EA, et al. Health of people experiencing co-occurring homelessness, imprisonment, substance use, sex work and/or severe mental illness in high-income countries: a systematic review and meta-analysis. J Epidemiol Community Health. 2021;75(10):1010-20. doi: 10.1136/jech-2020-215975.
6. Pleace N. At what cost? An estimation of the financial costs of single homelessness in the UK. London: Crisis; 2015. Available from: https://www.crisis.org.uk/media/237022/costsofhomelessness_finalweb.pdf.
7. Ponka D, et al. The effectiveness of case management interventions for the homeless, vulnerably housed and persons with lived experience: A systematic review. PLoS One. 2020;15(4):e0230896. doi: 10.1371/journal.pone.0230896.

For peer review only - http://bmjopen.bmj.com/site/about/guidelines.xhtml

8. Shaw J, et al. Critical time Intervention for Severely mentally ill Prisoners (CrISP): a randomised controlled trial. Health Serv Deliv Res. 2017;5(8):1-138. doi: 10.3310/hsdr05080.

9. Herman DB, Conover S, Gorroochurn P, Hinterland K, Hoepner L, Susser ES. Randomized trial of critical time intervention to prevent homelessness after hospital discharge. Psychiatr Serv. 2011;62(7):713-9. doi: 10.1176/appi.ps.62.7.713.

10. Kasprow WJ, Rosenheck RA. Outcomes of critical time intervention case management of homeless veterans after psychiatric hospitalization. Psychiatr Serv. 2007;58(7):929-35. doi: 10.1176/appi.ps.58.7.929.

11. Lako DAM, et al. The effectiveness of critical time intervention for abused women leaving women's shelters: a randomized controlled trial. Int J Public Health. 2018;63(4):513-23. doi: 10.1007/s00038-017-1067-1.

12. de Vet R, Beijersbergen MD, Jonker IE, Lako DAM, van Hemert AM, Herman DB, Wolf JRL. Critical time intervention for homeless people making the transition to community living: a randomized controlled trial. Am J Community Psychol. 2017;60(1-2):175-86. doi: 10.1002/ajcp.12150.

13. Ware J et al., Medical Care 34(3):p 220-233 (1996), A 12-Item Short-Form Health Survey: Construction of Scales and Preliminary Tests of Reliability and Validity, https://doi.org/10.1097/00005650-199603000-00003

14. Keeley T, Al-Janabi H, Lorgelly P, Coast J. A Qualitative Assessment of the Content Validity of the ICECAP-A and EQ-5D-5L and Their Appropriateness for Use in Health Research. PLoS One. 2013;8(12):e85287. doi: 10.1371/journal.pone.0085287.

15. Khadjesari Z, White IR, McCambridge J, Marston L, Wallace P, Godfrey C, Murray E. Validation of the AUDIT-C in adults seeking help with their drinking online. Addict Sci Clin Pract. 2017;12(2):2. doi: 10.1186/s13722-016-0066-5.

16. StataCorp. Stata Statistical Software: Release 18. College Station, TX: StataCorp LLC; 2023.

17. Braun V, Clarke V. Thematic Analysis: A Practical Guide. SAGE Publications; 2022.

18. Lumivero. NVivo (Version 12). Lumivero; 2017. Available from: https://www.lumivero.com

19. Ministry of Justice. Transparency Data End of Custody Supervised Licence (ECSL) data, England and Wales. 2024. Available from: https://assets.publishing.service.gov.uk/media/6690004049b9c0597fdafb6c/2024-07-11_ECSL_Transparency_data.pdf

20. Inside Time. Probation Reset' stops supervision of ex-prisoners. 2024. Available from: https://insidetime.org/newsround/probation-reset-stops-supervision-of-ex-prisoners/

For peer review only - http://bmjopen.bmj.com/site/about/guidelines.xhtml

544 21. Ministry of Justice. Offender management statistics quarterly: October to December 2023 and annual 2023. 2024. Available from: https://www.gov.uk/government/statistics/offender-management-statistics-quarterly-october-to-december-2023/offender-management-statistics-quarterly-october-to-december-2023-and-annual-2023

For peer review only

For peer review only - http://bmjopen.bmj.com/site/about/guidelines.xhtml

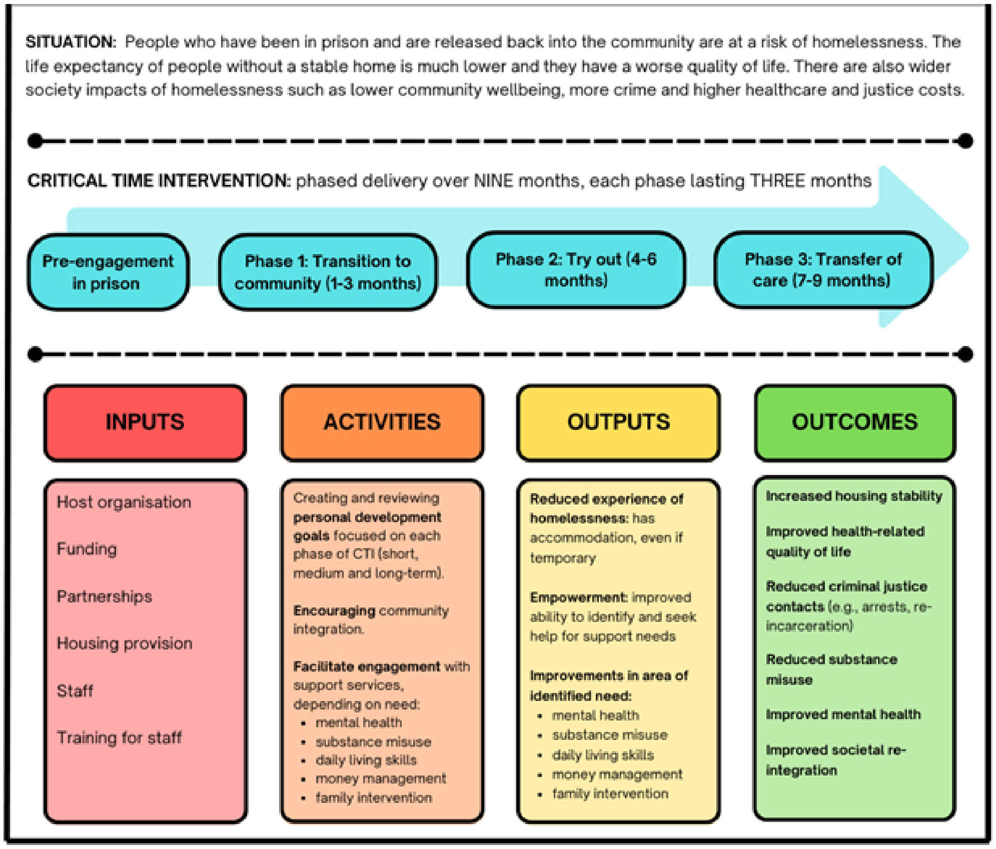

FIGURE 1. Housing led CTI logic model

414x352mm (38 x 38 DPI)

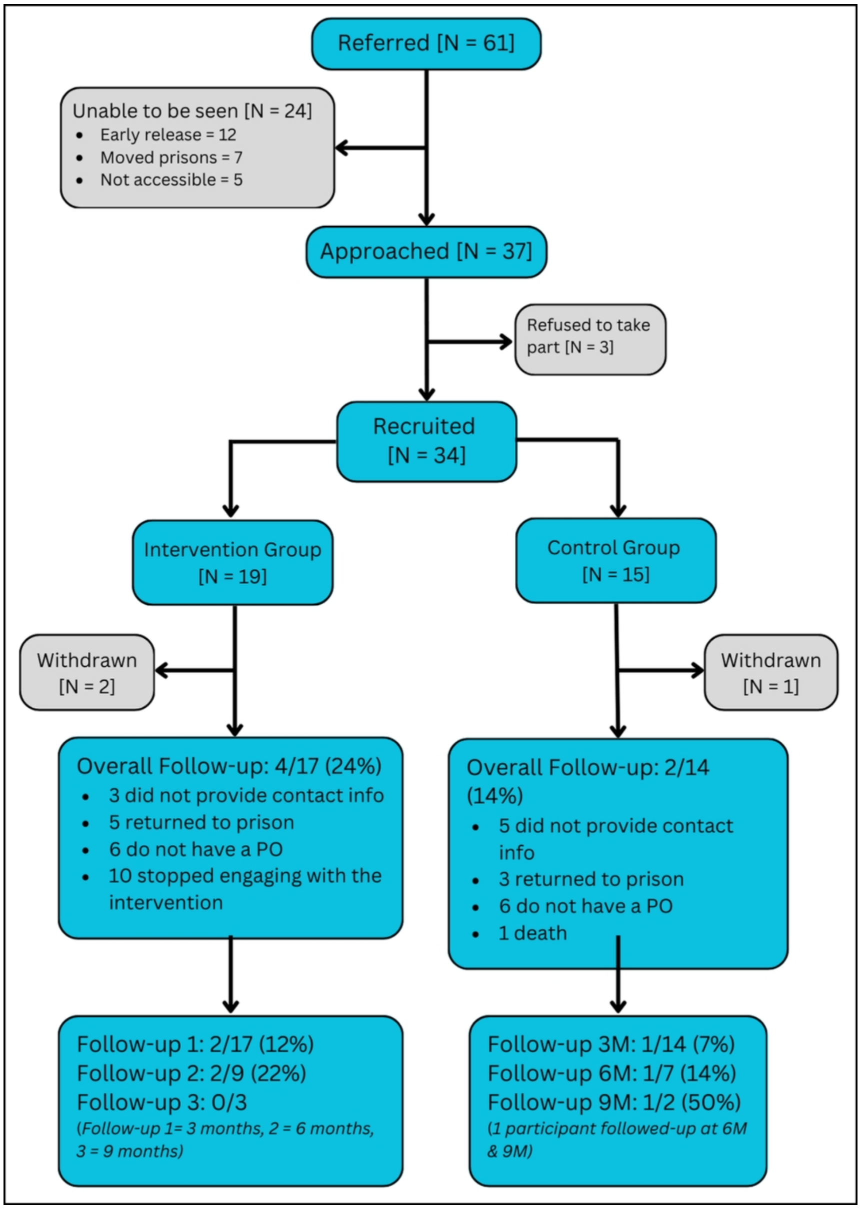

FIGURE 2. PHaCT CONSORT diagram
*The denominator in the fractions with respect to the follow-up at 3-, 6- and 9-months reflects the number of people who had the opportunity to be followed-up in the lifetime of the study.

389x547mm (113 x 113 DPI)

Supplementary Material - Results

Descriptive characteristics for all participants.

	n (%)		
	**Overall sample**	**Intervention**	**Control**
Overall number	**34 (100.0)**	**19 (55.9)**	**15 (44.1)**
Age in years, mean (SD)	38.05 (11.73)	38.68 (13.65)	37.27 (9.14)
Male sex	34 (100.0)	19 (100.0)	15 (100.0)
Ethnicity			
White	31 (91.28)	17 (89.5)	14 (93.3)
Mixed or multiple ethnic group	1 (2.94)	0 (0.0)	1 (6.7)
Caribbean or Black	1 (2.94)	1 (5.3)	0 (0.0)
Any other ethnic group (Bangladeshi)	1 (2.94)	1 (5.3)	0 (0.0)
Smoking status			
Never/Ex-smoker	8 (23.53)	5 (26.3)	3 (20.0)
Current smoker (not every day/every day)	26 (76.47)	14 (73.7)	12 (80.0)
In the 12 months before entering prison did you use illegal drugs?			
No	3 (8.82)	3 (15.8)	0 (0.0)
Yes	31 (91.18)	16 (84.2)	15 (100.0)
Education (could have more than one, selected the highest)			
No formal education	12 (35.29)	5 (26.3)	7 (46.7)
GCSE, GCE or O-level	8 (23.53)	5 (26.3)	3 (20.0)
Vocational training (e.g., Apprenticeship, City & Guilds)	12 (35.29)	9 (47.4)	3 (20.0)
Higher education	1 (2.94)	0 (0.0)	1 (6.7)
Do not know	1 (2.94)	0 (0.0)	1 (6.7)

Employment and benefit receipt before entering prison

Income source of participants before entering prison (part a) and the type of State benefits they received before entering prison.

(a) Source of income before entering prison	N	(b) In receipt of State benefits	N
State benefits only	17	No benefits	6
State benefits & employed	1	Universal credit only	8
State benefits & self employed	1	Universal credit and housing benefits	5
State benefits & proceeds of crime	6	Universal credit, and housing benefits council tax reduction and job seeker's allowance	1
State benefits & help from family/friend	1	Universal credit and sickness or disability	1
State benefits & other (not specified)		Job seeker allowance	1
Employed/self-employed	4	Pension benefit	1
Proceeds of crime	2	Sickness or disability	1
Missing/no response	1		

For peer review only

Note: multiple response allowed

Secondary outcomes results

Immediately before entering prison, were you in settled accommodation (this is medium to long term accommodation such as social housing or a private rented or owner-occupied home)?			
No	25 (73.53)	12 (63.2)	13 (86.7)
Yes	9 (26.47)	7 (36.8)	2 (13.3)
If No to the previous question (n=25)			
Hostel	7 (28.00)	3 (25.0)	4 (30.8)
Emergency accommodation (B&B or night shelter)	3 (12.00)	1 (8.3)	2 (15.4)
A caravan or a squat	1 (4.00)	1 (8.3)	0 (0.0)
Temporarily at a friend's or family's house –on an informal basis (sofa surfing)	7 (28.00)	2 (16.7)	5 (38.5)
Rough sleeping, on transport or in transport hub (bus stop or train station), in a tent or car	2 (8.00)	0 (0.0)	2 (15.4)
Other	5 (20.00)	5 (41.7)	0 (0.0)
**Times in life been homeless (Median, IQR)**	3. 0 (2 to 4); n=29	2.0 (1 to 2); n=17	3.5 (3 to 5), n=12
Once	7 (20.59)	6 (31.6)	1 (6.7)
2-9 times	19 (55.88)	10 (52.6)	9 (60.0)
10 or more	3 (8.82)	1 (5.3)	2 (13.3)
Do not know	3 (8.82)	2 (10.5)	1 (6.7)
Missing	2 (5.88)	0 (0.0)	2 (13.3)
**AUDIT-C* 12 months prior prison (Median IQR)**	5.5 (0 to 12)	3 (0 to 12)	9 (0 to 12)
**Visited a GP in prison**			
No	15 (44.12)	9 (47.4)	6 (40.0)
Yes	19 (55.88)	10 (52.6)	9 (60.0)
**Visited a nurse in prison**			
No	10 (29.41)	5 (26.3)	5 (33.3)
Yes	24 (70.59)	14 (73.7)	10 (66.7)
Have you had a hospital admission while in prison			
No	29 (85.29)	15 (78.9)	14 (93.3)
Yes	5 (14.71)	4 (21.1)	1 (6.7)
**Have used any substance use services (e.g. alcohol or drug treatment or support)?**			
No	15 (44.12)	12 (63.2)	3 (20.0)
Yes	19 (55.88)	7 (36.8)	12 (80.0)
Have you had any other healthcare contact / contact with other healthcare worker?			
No	26 (76.47)	12 (63.2)	14 (93.3)
Yes	8 (23.53)	7 (36.8)	1 (6.7)
**Do you currently have any supportive relationships?**			
No supportive relationships	10 (29.41)	8 (42.1)	2 (13.3)
Supportive relationships with friends	2 (5.88)	1 (5.3)	1 (6.7)
Supportive relationships with family	6 (17.65)	2 (10.5)	4 (26.7)
Supportive relationships with family and friends	16 (47.06)	8 (42.1)	8 (53.3)

**ICECAP_A overall score (median, IQR)**	0.672 (0.373 to 0.888)	0.698 (0.373 to 0.888)	0.589 (0.36 to 0.898)

*AUDIT-C has three questions, each scored from 0 to 4 resulting in a total score on a scale of 0 to 12 (scores of 0 reflect no alcohol use). In men, a score of 4 or more is considered positive. The higher the AUDIT-C score, the more likely it is that the participant's drinking is affecting health and safety.)

Health Economics results

1. **Intervention Costs**

We liaised with the CTI team, using and adapting their data collection systems to capture the cost of delivering CTI. We captured a broad set of intervention cost components, including: staff costs (i.e. time the CTI staff spends with the participant or communicating with an external agency, such as addiction service), housing costs (i.e. cost of paying rent or purchasing household equipment), travel cost, administrative costs (i.e. time spent by the CTI staff to review and discuss cases) and miscellaneous costs (i.e. any other costs recorded that does not fall under any of the categories above, e.g. costs for clothing).
Table 1 shows the average and total cost of the CTI intervention, by intervention phase. Of the 17 participants randomized to the CTI intervention, 13 completed the pre-engagement phase and phase 1, 9 progressed through phase 2 and only 2 participants completed all the intervention stages. The average cost per participant is £165.5 for the pre-engagement phase only; £194.5 for phase 1, £75.9 for Phase 2, £157.3 for Phase 3. The average cost for those who progress through all the 4 phases of was £674.6.

Table 1 Mean and Median Intervention Cost per Participant by Phases

Phase (n)	**Pre-engagemen t (17)**	**Phase 1 (13)**	**Pre-engagement + Phase 1 (13)**	**Phas e 2 (9)**	**Pre-engagemen t to Phase 2 (9)**	**Phase 3 (2)**	**Pre-engagemen t to Phase 3 (2)**
Mean	£165.5	£194.5	£248.6	£75.9	£355.1	£157.3	£674.6
Median	£74.7	£198.4	£263.7	£37.2	£311.5	£157.3	£674.6

- n= the number of participants who were enrolled in phase

Table 2 shows the total cost of the CTI intervention, by intervention phase and cost category. The cost of staff delivering the intervention represents the main cost component, accounting for 46.3% of total overall costs.

These estimates need to be taken with caution. First, descriptive statistics are based on a very small number of observations, overall and across CTI phases. Delays in recruitment have led to participants not completing all the four phases of the CTI intervention. Descriptive statistics are thus based on a different number of observations, with only 2 participants completing all the CTI intervention phases (from pre-engagement to phase 3). Also, while we initially aimed to collect travel costs as both the actual cost sustained to travel (retrieved from travel claims) and the opportunity costs (i.e. time spent by staff travelling, multiplied by the hourly staff wage), it was not possible to collect the staff opportunity cost of travel to prison. Also, housing costs were only recorded for one participant.

Table 2 Total Intervention Cost by Cost categories and phases

Phases / ***Cost categories***	**Pre-engagement**	**Phase 1**	**Phase 2**	**Phase 3**	**All phases**
Staff Cost	£775.1	£1,585.3	£380.1	£201.3	£2,941.8

For peer review only

% Staff cost	27.5%	62.6%	54.9%	63.9%	46.3%
Travel Cost	£96.8	£147.6	£9.2	£17.8	£271.4
%Travel Cost	3.4%	5.8%	1.3%	5.7%	4.3%
Miscellaneous Cost	£3.6	£87.5	£0.0	£10.0	£101.1
% Miscellaneous	0.1%	3.5%	0.0%	3.2%	1.6%
Housing Cost	£1,467.9	£0.0	£0.0	£0.0	£1,467.9
%Housing Cost	52.1%	0.0%	0.0%	0.0%	23.1%
Review/Admin Cost	£475.8	£710.2	£302.7	£85.5	£1,574.2
% Review Admin Cost	16.9%	28.1%	43.7%	27.2%	24.8%
Total Cost	£2,819.2	£2,530.5	£692.1	£314.6	£6,356.4

2. EQ-5D and ICECAP-A descriptive statistics

Table 3 shows average EQ-5D-3L and ICECAP-A scores at baseline for the 34 participants. The average EQ-5D-3L score of participants at baseline is 0.57 (SD=0.42), which is lower than the population norm[1]. The average ICECAP-A score of participants at baseline is 0.62 (SD=0.29); this is similar to a previous study, considering the population[2].

Table 4 summarizes EQ-5D and ICECAP-A scores by homelessness status and use of drugs and tobacco before entering prison. As expected, drug and tobacco users have a lower quality of life and capability score than non-users, while the association between living in a settled accommodation before entering prison and wellbeing and capability score seems to go in the opposite direction.

Table 3

**EQ-5D-3L and ICECAP-A, average value at baseline**			
	N	Mean	SD
**EQ-5D-3L index**	34	**0.577**	0.420
**ICECAP-A index**	34	**0.615**	0.285

Table 4

**EQ-5D index and ICECAP-A index by key categories**						
**Lived in settled accommodation before entering prison**						
	**Yes**			**No**		
	N	Mean	SD	N	Mean	SD
**EQ-5D index**	9	**0.492**	0.484	25	**0.608**	0.401
**ICECAP-A index**	9	**0.597**	0.374	25	**0.621**	0.254
**Drug and tobacco use before entering prison**						
	**Yes**			**No**		
	N	Mean	SD	N	Mean	SD
**EQ-5D index**	31	**0.565**	0.434	3	**0.703**	0.251
**ICECAP-A index**	31	**0.603**	0.287	3	**0.733**	0.279

When considering EQ-5D-3L single dimension, we found that the percentage of participants reporting severe or moderate problems is lower to the general population across all dimensions. This is

especially true for anxiety and depression, where 47% and 27% of participants report experiencing moderate and severe depression and anxiety (vs. respectively, 19% and 1.8% in the general population).

3. Resource use at baseline

Table 5 shows descriptive statistics on healthcare resource use in prison (visits to the GP, nurse, substance use services and hospital admission).

Table 5

**Visited GP in prison**			
	N	%	**Number of visits (mean, SD)**
Yes	19	55.88	4.9 (8.1)
No	15	44.12	
**Visited nurse in prison**			
	N	%	**Number of visits (mean, SD)**
Yes	24	70.59	10.2 (20.1)
No	10	29.41	
**Hospital admission**			
	N	%	**Number of hospital admissions (mean, SD)**
Yes	5	14.71	1.6 (0.89)
No	29	85.29	
**Substance use services**			
	N	%	**Number of visits (mean, SD)**
Yes	19	55.88	2.2 (26)
No	15	44.12	
**Other**			
	N	%	**Number of visits (mean, SD)**
Yes	8	23.53	2.8 (3.2)
No	26	76.47	

References

1. Kind P, Hardman G, Macran S. *UK population norms for EQ-5D*. York: Centre for Health Economics, University of York; 1999.

2. Hunter RM, Anderson R, Kirkpatrick T, Lennox C, Warren F, Taylor RS, et al. Economic evaluation of a complex intervention (Engager) for prisoners with common mental health problems, near to and after release: a cost-utility and cost-consequences analysis. *Eur J Health Econ*. 2022;23(2):193–210. doi: 10.1007/s10198-021-01360-7

For peer review only

PHACT PROTOCOL

Preventing Homelessness, improving health for people leaving prison: a pilot randomised controlled trial of a Critical Time Intervention

V2.2 11.04.2024

**Sponsor:**	University of Glasgow<br>University Avenue, Glasgow G12 8QQ
**Sponsor ref:**	N/P
**Funder:**	NIHR
**Funder ref:**	NIHR134281
**REC ref:**	22/WA/0347
**IRAS number:**	319937
**ISRCTN ref:**	46969988
**Q-Pulse Document Template Number:**	TPL/003/2

For peer review only - http://bmjopen.bmj.com/site/about/guidelines.xhtml

SIGNATURE PAGE

The undersigned confirm that the following protocol has been agreed and accepted and that the Chief Investigator agrees to conduct the trial in compliance with the approved protocol and will adhere to the principles outlined in the relevant trial regulations, GCP guidelines, and CTR's SOPs.

I agree to ensure that the confidential information contained in this document will not be used for any other purpose other than the evaluation or conduct of the clinical investigation without the prior written consent of the Sponsor.

I also confirm that I will make the findings of the trial publicly available through publication or other dissemination tools without any unnecessary delay and that an honest accurate and transparent account of the trial will be given; and that any discrepancies from the trial as planned in this protocol will be explained.

**Trial sponsor:**			
Emma-Jane Gault	Research Regulation & Compliance Manager	EJ Gault	14.05.2024
Name	*Position*	*Signature*	*Date*
**Director:**			
Prof Mike Robling	Co-Director, Centre for Trials Research	M Robling	15.04.2024
Name	*Position*	*signature*	*Date*
**Chief Investigator:**			
Prof Jim Lewsey	Professor of Medical Statistics	J Lewsey	14.04.2024
Name	*Position*	*Signature*	*Date*

General Information This protocol describes the PHaCT clinical trial and provides information about the procedures for entering participants into the trial. The protocol should not be used as a guide, or as an aide-memoire for the treatment of other participants. Every care has been taken in drafting this protocol; however, corrections or amendments may be necessary. These will be circulated to the known Investigators in the trial. Problems relating to the trial should be referred, in the first instance, to CTR.

For peer review only - http://bmjopen.bmj.com/site/about/guidelines.xhtml

Contact details – Chief Investigator / Co-Investigators

CHIEF INVESTIGATOR

**Title and name**	**Professor Jim Lewsey**
**tr**	Professor of Medical Statistics
**Address**	Institute of Health and Wellbeing<br>University of Glasgow<br>1 Lilybank Gardens<br>Glasgow
**Postcode**	G12 8RZ
**Tel**	0141 330 3260
**E-mail**	jim.lewsey@glasgow.ac.uk

CO-INVESTIGATORS

Dr Rebecca Cannings-John
Position: Principal Research Fellow in Statistics, Centre for Trials Research, Cardiff University
E-mail: canningsrl@Cardiff.ac.uk

Dr Manuela Deidda Position: Research Fellow in Health Economics, Institute of Health and Wellbeing, University of Glasgow
E-mail: Manuela.Deidda@glasgow.ac.uk

Professor Suzanne Fitzpatrick
Position: Professor of Housing and Social Policy, School of Energy, Geoscience, Infrastructure and Society, Heriot Watt University
E-mail: S.Fitzpatrick@hw.ac.uk

Professor Peter Mackie
Position: Professor of Housing, School of Geography and Planning, Cardiff University
E-mail: MackieP@cardiff.ac.uk

Professor Iolo Madoc-Jones
Position: Professor in Criminal and Social Justice, Glyndŵr University
E-mail: i.m.jones@glyndwr.ac.uk

Mr Ben Mason
Position: Lived experience specialist, Centre for Homelessness Impact
E-mail: ben@homelessnessimpact.org

Professor Vittal Katikireddi
Position: Professor of Public Health & Health Inequalities, University of Glasgow
E-mail: vittal.katikireddi@glasgow.ac.uk

Ms Yvonne Moriarty
Position Research Fellow/Senior Trial Manager, Centre for Trials Research, Cardiff University
E-mail: MoriartyY@cardiff.ac.uk

Dr Ian Thomas
Position: Research Associate in Administrative Data, Wales Institute of Social and Economic Research, Cardiff University
E-mail: thomasir2@cardiff.ac.uk

Professor James White
Position: Professor of Epidemiology and Public Health, Deputy Director of Population Health Trials, Centre for Trials Research, Cardiff University
E-mail: WhiteJ11@cardiff.ac.uk

Trial Co-ordination:

For peer review only - http://bmjopen.bmj.com/site/about/guidelines.xhtml

The PHaCT trial is being coordinated by the Centre for Trials Research (CTR), Cardiff University, a Clinical Research Collaboration (UKCRC) registered trials unit.

This protocol has been developed by the PHaCT Trial Management Group (TMG).

For **all queries** please contact the PHaCT team through the main trial email address. Any clinical queries will be directed through the Trial Manager to either the Chief Investigator or a Co-Investigators

**Main Trial Email:**	PHaCT@Cardiff.ac.uk	
Trial Administrator:	Ms Kelly Lewis	Tel: 02922 514511
Senior Trial Manager:	Ms Yvonne Moriarty	Email: MoriartyY@cardiff.ac.uk
Trial Manager	Mr Adam Williams	Email: WilliamsAD7@cardiff.ac.uk
Data Manager:	Mr Barry Lloyd	Email: LloydB5@cardiff.ac.uk
Trial Statistician:	Dr Detelina Grozeva	Email: GrozevaD@cardiff.ac.uk
Qualitative Researcher	Dr Jacqueline Hughes	Email: HughesJ33@cardiff.ac.uk
Senior Health Economist	Dr Manuela Deidda	Email: Manuela.Deidda@glasgow.ac.uk
Health Economist	Samuel Owusu Achiaw	Email:Samuel.OwusuAchiaw@glasgow.ac.uk
CTR Director:	Professor Michael Robling	Email: roblingmr@cardiff.ac.uk

Queries:

Queries

PHaCT@Cardiff.ac.uk

All queries will be directed to the most appropriate person.

Safeguarding concerns:

Safeguarding reporting

Any safeguarding concerns should be reported within 24 hours of becoming aware of them either by:

1. **Contacting the trial administrator on 02922 514511 or PHaCT@cardiff.ac.uk using only the Participants PID number.**

For peer review only - http://bmjopen.bmj.com/site/about/guidelines.xhtml

Table of Contents

1. Amendment History 7
2. Synopsis 8
3 Trial summary & schema 10
3.1 Full study schema (v2 17.07.2023) 10
3.2 RCT Participant flow diagram (v1.1 17.10.2022) 11
3.3 Trial lay summary 12
4 Background 13
4.1 Rationale for current pilot trial 16
5 Trial objectives/endpoints and outcome measures 16
5.1 Primary outcomes measure(s) 17
5.2 Secondary outcomes measures 17
6 Trial design and setting 18
6.1 Risk assessment 18
7 Site and Investigator selection 19
8 Participant selection 19
8.1 RCT Inclusion criteria 19
8.2 RCT Exclusion criteria 19
8.3 NON-RCT Inclusion criteria .... **Error! Bookmark not defined.**
9. Recruitment, Screening, and Registration 19
9.1 RCT PARTICIPANTS 19
9.1.1 Participant identification: 19
9.1.2 Screening logs: 20
9.1.3 Recruitment: 20
9.1.4 Informed consent: 20
9.1.5 Randomisation: 21
9.2 NON-RCT PARTICIPANTS 21
9.2.1 Participation identification: 21
9.2.2 Recruitment: 21
9.2.3 Informed consent: 21
10. Withdrawal & lost to follow-up 22
10.1 RCT Withdrawal 22
10.2 RCT Strategies to minimise loss to follow up 22
10.3 NON-RCT Withdrawal 22
11. Trial Intervention 23
11.1 Critical Time Intervention 23
11.2 Compliance 24
11.3 Training 24
12 Trial procedures 24
12.1 Assessments 28
12.2 Follow-up 29
13 Safety reporting 29
13.1 Definitions 30
13.2 Reporting process 31
14 Statistical considerations 31
14.1 Randomisation 31
14.2 Blinding 31
14.3 Sample size 32
14.4 Missing, unused & spurious data 32
14.5 Procedures for reporting deviation(s) from the original SAP 32
14.6 Termination of the trial 32

For peer review only - http://bmjopen.bmj.com/site/about/guidelines.xhtml

14.7 Inclusion in analysis 32
15 Analysis 32
15.1 Main analysis 32
15.1.1 Sub-group & interim analysis 33
15.2 Qualitative analysis 33
15.3 Economic evaluation 34
15.4 Routine data analysis 35
16 Data Management 36
16.1 Data collection 37
16.2 Completion of CRFs 38
16.2.1 Paper CRFs 38
16.2.2 Electronic CRFs 38
17 Protocol/GCP non-compliance 38
18 End of Trial definition 39
19 Archiving 39
20 Regulatory Considerations 39
20.1 Ethical and governance approval 39
20.2 Data Protection 39
20.2.1 Data sharing 40
20.3 Indemnity 40
20.4 Trial sponsorship 40
20.5 Funding 40
21 Trial management 40
21.1 TMG (Trial Management Group) 40
21.2 TSC (Trial Steering Committee) 40
22 Quality Control and Assurance 41
22.1 Monitoring 41
22.2 Audits & inspections 41
23 Publication policy 41
24 Milestones 41
25 References 42
26 Appendices 47
26.1 Logic model 47

For peer review only

Glossary of Abbreviations

**AUDIT-C**	Alcohol Use Disorders Identification Test-Consumtion
**CI**	Confidence interval
**CONSORT**	Consolidated Standards of Reporting Trials
**CRF**	Case Report Form
**CRN**	Clinical Research Network
**CTI**	Critical Time intervention
**CTR**	Centre for Trials Research
**DMC**	Data Monitoring Committee
**DMP**	Data Management Plan
**EoI**	Expression of interest
**EQ-5D**	EuroQoL standardised measure of health-related quality of life
**GCP**	Good Clinical Practice
**HMP**	Her Majesty's Prison
**ICECAP-A**	ICEpop CAPability measure for Adults
**ICH**	International Conference on Harmonization
**IQR**	Interquartile Ranges
**ISRCTN**	International Standard Randomised Controlled Trial Number
**MoJ**	Ministry of Justice
**MRC**	Medical Research Council
**NICE**	National Institute for Health and Care Excellence
**NIHR**	National Institute for Health and Care Research
**NHS**	National Health Service
**NW CRN**	Northwest Clinical Research Network
**PID**	Participant Identification Number
**PPI**	Patient and Public Involvement
**QA**	Quality Assurance
**QALY**	Quality-adjusted Life Years
**QAP**	Qualitative data collection and Analysis Plan
**QC**	Quality control
**QL (QoL)**	Quality of Life
**R&D**	Research and Development
**RCT**	Randomised Controlled Trial
**REC**	Research Ethics Committee
**SAIL**	Secure Anonymised Information Linkage
**SAP**	Statistical Analysis Plan
**SD**	Standard Deviation
**SF-12**	12-item Short Form health survey
**SOP**	Standard Operating Procedure
**TMF**	Trial Master File
**TMG**	Trial Management Group
**TSC**	Trial Steering Committee
**UK**	United Kingdom
**VOI**	Value of Information

For peer review only

For peer review only - http://bmjopen.bmj.com/site/about/guidelines.xhtml

1. Amendment History

The following amendments and/or administrative changes have been made to this protocol since the implementation of the first approved version.

Amendment No.	Protocol version no.	Date issued	Summary of changes made since previous version
3	2.2	11.04.2024	1. Change to progression criteria, removal of 12-month specification.<br>2. Change to end date.
2	2.1	04.01.2024	3. Added 5th potential site, HMP Parc.<br>4. General edits to language.
1	2.0	11.07.2023	1. Updated team members details<br>2. Updated references to 'NHS Digital' to 'NHS England' in line with recent name change<br>3. Inclusion of non-RCT participant interviews and observations to expand recruitment pool.<br>4. Broadening of recruiting team from 'R&D/CRN' to 'recruiter' to enable Cardiff trial team study staff to support recruitment in local sites.<br>5. Added ability to book follow-up visit appointment for baseline selection following TSC advice due to short prison slots.<br>6. Updated Logic model to map against key research questions

For peer review only

2. Synopsis

**Short title**	Preventing Homelessness, improving health for people leaving prison: a pilot randomised controlled trial of a Critical Time intervention
**Acronym**	PHaCT
**Internal ref. no.**	1095
**Development phase**	Phase II
**Funder and ref.**	NIHR134281
**Trial design**	Pilot Randomised controlled trial
**Trial participants**	Men at risk of homelessness leaving prison
**Planned sample size**	80 (1:1 randomisation intervention and usual care) allowing for 40% drop out at final follow-up
**Planned number of sites**	5 prisons (HMP Liverpool, HMP Altcourse, HMP Risley, HMP Swansea and HMP Parc)
**Inclusion criteria**	1. Aged over 18 years.<br>2. Released into the Swansea/Neath or Liverpool/Merseyside local authority areas and have a local connection (e.g., lived their previously; close relatives living in these areas)<br>3. Recourse to public funds<br>4. Have experienced prison and homelessness at least once.<br>5. Mental health or substance use support needs
**Exclusion criteria**	1. Individuals under the Multi-Agency Public Protection arrangements 3 (MAPPA 3)<br>2. Those benefitting from Housing First
**Treatment duration**	9 months consisting of 3 phases lasting 3 months each ('Transition to the Community', 'Try out', 'Transfer of Care')
**Follow-up duration**	3-, 6-, 9- and 12-months post randomisation
**Planned trial period**	33 months
**Primary objective**	To conduct a pilot RCT of housing led CTI for males leaving prison at risk of homelessness to determine if a full scale RCT is warranted.
**Secondary objectives**	1. To identify suitable and acceptable trial methods (recruitment, randomisation, intervention delivery, retention).<br>2. To identify feasible data collection methods and acceptable outcome measures.<br>3. To explore potential for data linkage in this population (acceptability in terms of consent, suitable data sets).
**Primary outcomes**	Meeting progression criteria to full scale trial:<br>1. Recruitment of 50% of those approached<br>2. Retention of 60% of those enrolled atpost randomisation<br>3. Acceptability of intervention delivery to participants and staff<br>4. Intervention delivered with fidelity

For peer review only - http://bmjopen.bmj.com/site/about/guidelines.xhtml

**Secondary outcomes**	1. Health-related quality of life (SF-12, (total, physical and mental health scores); EQ-5D; ICECAP-A)<br>2. Housing stability (i.e., number of days in a stable accommodation)<br>3. Substance use and hazardous alcohol consumption<br>4. Deaths (including cause of death)
**Tertiary/Exploratory outcomes**	We will investigate the possibility of gaining data on:<br>1. Re-incarceration<br>2. Support networks<br>3. Crime (e.g., criminal arrest, criminal convictions, police contacts, rearrest)<br>4. Education training and employment<br>5. Finance, benefits and debt<br>6. Support network/family & friend connections<br>7. Wellbeing<br>8. Community integration and engagement
**Intervention**	Critical Time Intervention (CTI) delivered by Crisis charity case workers to improve an individual's engagement with treatment and community services through developing problem-solving skills and are targeted to continuity of care during a period of transition (i.e., release from prison)

For peer review only

3 Trial summary & schema

3.1 Full study schema (v2 17.07.2023)

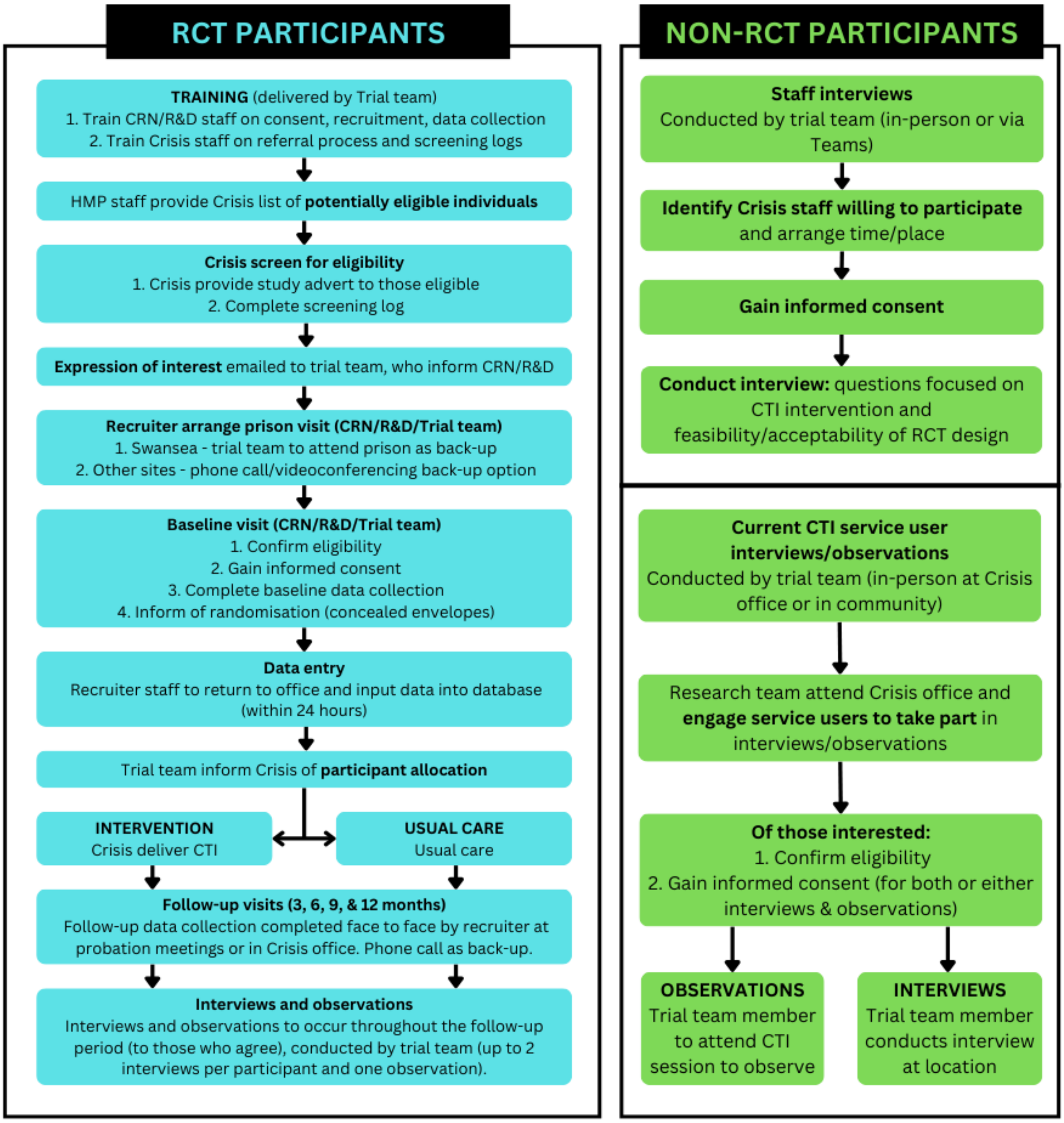

For peer review only - http://bmjopen.bmj.com/site/about/guidelines.xhtml

3.2 RCT Participant flow diagram (v1.1 17.10.2022)

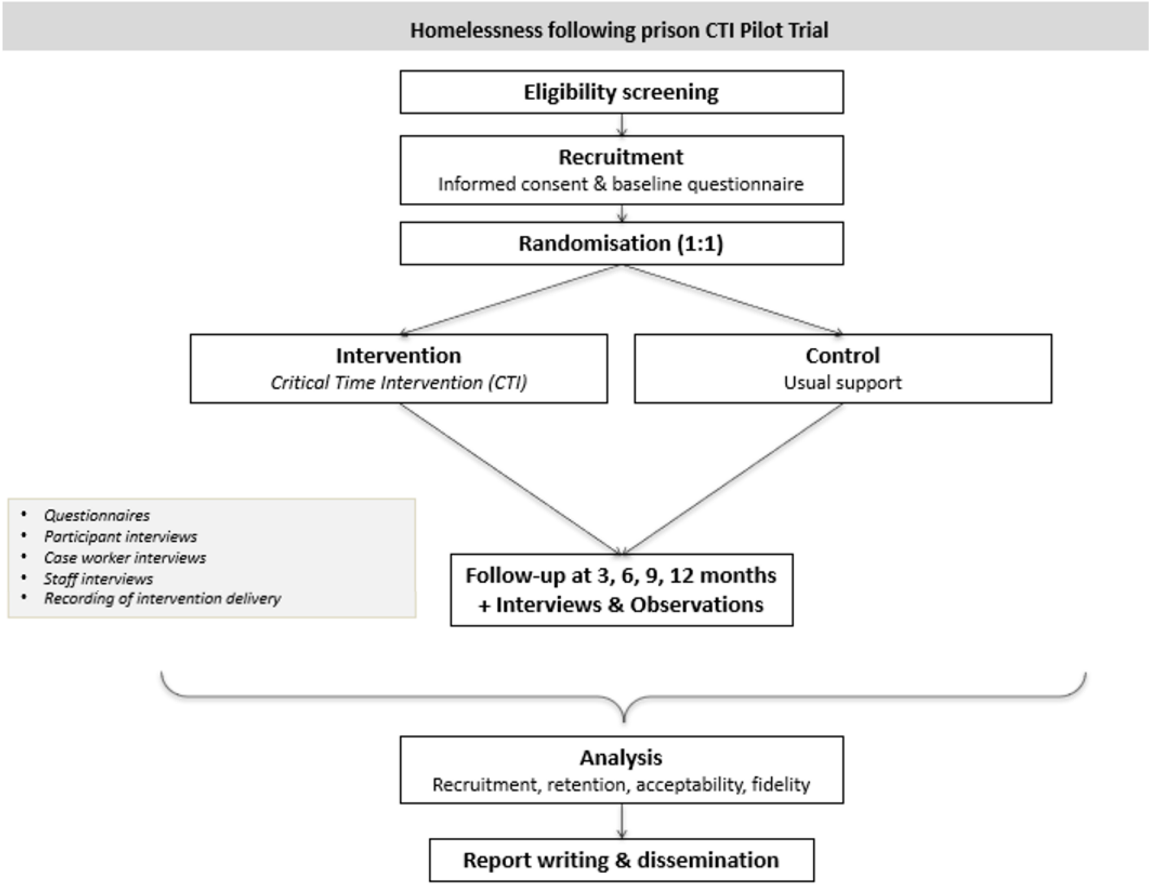

For peer review only - http://bmjopen.bmj.com/site/about/guidelines.xhtml

3.3 Trial lay summary

People who have been in prison and are released back into the community are at a high risk of homelessness. Nearly 1 in 2 who are released do not have a settled home and 1 in 3 are homeless or are in unsettled housing one year later. The life expectancy of people without a stable home is much lower and they have a worse quality of life. There are also wider society impacts of homelessness such as lower community wellbeing, more crime and higher healthcare and justice costs. Crisis (a national charity for people experiencing homelessness) have calculated that on average the yearly cost of one-person rough sleeping could cost £20,128. Although there are good interventions to stop homelessness, they are mostly from the US, and we do not know how these work for people who have been in prison. Critical Time Intervention (CTI) is the name of a type of intervention which aims to support those most in need in society during times of important life changes. They help people at risk of homelessness stabilise their housing situation, particularly those people who are leaving institutions (like prisons). Homelessness services think that this type of intervention works best when it is 'housing-led'. This is where people are given housing quickly without conditions. There have been no studies which have looked at how good housing led CTIs are at stopping homelessness and better the health of people with prison experience in the UK and if they are good value for money.

Working with Crisis we will carry out a 'pilot Randomised Controlled Trial (RCT)' (where people are randomly allocated to one of two groups: CTI or usual care) in four prison populations in England and Wales. In the long term we plan to test if a housing-led CTI betters people's health-related quality of life and housing ('effectiveness') and is 'cost-effective' (i.e., its good value for money), but first we need to find out if the intervention and study design (research methods) are acceptable.

We will recruit 80 adult males who are ready to leave one of 4 prisons (HMP Liverpool, HMP Altcourse, HMP Risley and HMP Swansea) who are at risk of homelessness to take part. Participants will be asked to complete a survey asking about their health-related quality of life, housing stability, substance misuse, alcohol consumption, re-incarceration, support networks etc. We will also ask their permission to access anonymous routinely collected health, educational and aggregated criminal justice information. Participants will then be randomly allocated to either get the CTI or usual care. We will follow-up participants at 3, 6, 9 and 12 months. Some participants, prison staff and intervention staff will also be asked to take part in face-to-face interviews to find out more about their experiences of taking part to find out if they think the intervention and recruitment methods are feasible and acceptable to them. Some of their sessions with their CTI worker will also be observed to see how the CTI intervention is being delivered.

For peer review only - http://bmjopen.bmj.com/site/about/guidelines.xhtml

4 Background

For people leaving prison, re-integrating into the community can be a difficult process. Substance use, poor mental health, personal and situational factors such as low levels of literacy and absence of a stable support network [1], reinforced by a lack of affordable housing and employment opportunities, make them particularly at risk of homelessness. The absence of stable accommodation has a devastating impact on physical and mental health – people experiencing homelessness are more exposed than the general population to infectious and noncommunicable diseases [2], mental health problems (including alcohol and substance use) [3], have higher rates of emergency hospital admissions [4] and report lower levels of wellbeing and health-related quality of life [5, 6]. Poor nutrition, inadequate self-management of preventable conditions and poor adherence to medications contribute to an all-cause mortality rate which is between 3 and 11 times higher in people experiencing homelessness, compared to other socio-economically disadvantaged people [7, 8]. Further, absence of stable accommodation is also strongly associated with unemployment [9] and criminal activity [10, 11]. Homelessness also has a broader societal impact; in terms of decreased community wellbeing (e.g., perceived safety) and higher costs (e.g., increased utilisation of judicial services and welfare sectors) [12]. An analysis conducted by Crisis, a national charity for people experiencing homelessness, has estimated the average annual cost associated with a single case of street homelessness could reach £20,128 [13]. In consideration of its detrimental health and societal consequences, homelessness is a major public health issue which demands an effective preventive approach. This will generate substantial benefits in terms of housing stability, health and wellbeing, and wider societal cost savings.

A UK survey conducted with nearly 500 single people experiencing homelessness across 16 local authorities found that 41% of the interviewees had once served a prison sentence [14]. Although the need to provide housing assistance to those leaving prison has been recognised in homelessness and offender policy and legislation across the UK administrations (e.g., the Offender Rehabilitation Act (2014) [15], the Housing (Wales) Act (2014) [16], the Homelessness Reduction Act (2017) [17]), to date, efforts to meet the housing needs of people on release from prison have not proven effective [18], nor public health interventions routinely implemented to support people in prisons during the critical transition to the community.

Critical Time Interventions (CTIs) are focused and time-limited case management interventions that aim to improve an individual's engagement with treatment and community services through developing problem-solving skills and are targeted to continuity of care during a period of transition

For peer review only - http://bmjopen.bmj.com/site/about/guidelines.xhtml

(e.g., release from prison) [19]. The key elements of CTIs are small caseloads, active community outreach, individualised case management plans, psychosocial skill building, motivational coaching, with a phased approach that decreases in intensity over time [20]. CTIs, by providing emotional and practical support during critical times, endeavour to strengthen long-term ties to services as well as family and friends [21].

A CTI was first developed and evaluated for people with mental illness who were transitioning from shelters to housing in the community [22] and has been adapted and used in other settings (e.g., after discharge from psychiatric hospitalisation [23], for families experiencing homelessness and mental health problems [24] and housing-unstable families with children in foster care [25]).

A key concern for people leaving prison is access to suitable accommodation upon release. In a nested qualitative study as part of a randomised controlled trial (RCT) of CTI for people in prison with severe mental illness [26], health professionals stated that securing suitable accommodation was arguably the top priority for people newly released from prison because it provides a stable base from which to address other re-integration into the community concerns. This was echoed in the views of the RCT participants who stated that finding suitable accommodation was vital for accessing key services and reducing their risk of future reoffending and incarceration [27]. In a mixed-methods study examining the needs and obstacles people who had been in prison face upon release [28], most participants identified housing as crucial in promoting successful re-integration into the community. These findings are consistent with 'Housing First' interventions (assisting people with access to permanent housing as the key initial step in addressing homelessness) being successful in improving housing stability and some aspects of health [29].

For peer review only

Existing evidence:

In a systematic review of case management for people experiencing homelessness [30], two CTI studies were identified – one RCT [31] and one historically controlled study [23]. In the RCT, both the CTI and control groups (usual services only) were discharged from an on-site psychiatry program in a shelter to community-based housing in the New York City region. The CTI group reported significantly fewer homeless nights and a decrease in negative psychiatric symptoms. In the historically controlled study, set in eight sites in the US, housing was not provided as part of the CTI. Those receiving CTI spent more days housed, fewer days in institutional settings, lower alcohol/drug use and less psychiatric problems than those receiving usual services.

In a more recent systematic review of case management for people experiencing homelessness or people who are vulnerably housed [19], five CTI RCT studies were identified – one identified in the

For peer review only - http://bmjopen.bmj.com/site/about/guidelines.xhtml

previous review [31], with the others being set in the Netherlands [32, 33] and the New York region [21, 24, 34]. Of the four RCTs that had housing stability outcomes, one found no significant improvement in outcome for the CTI group [32], two found significant reductions in odds of homelessness [21, 31] and one found higher rates of time spent in housing for the CTI group, but findings converged when CTI services ended [24]. All studies (or in follow-up research) had mental health outcomes; two finding no significant improvement in outcomes [32, 33], one showing a reduction in internalising and externalising behaviours in children under 5 years [24] but no effect on maternal mental health [34], one finding decreases in negative psychiatric symptoms [31] and one finding decreases in psychiatric re-hospitalisation [35]. Only two studies had quality of life as an outcome of interest and both reported no differences between CTI and control group [32, 33].

From our own literature searches using key terms in the aforementioned reviews, and from citation tracking of the key studies above, we found two further CTI RCTs [26, 36], a before and after study [37], a cost-effectiveness study (from a RCT) [38] and a rapid evidence assessment of homelessness services [39]. The effectiveness studies found that those in CTI group had greater continuity of psychiatric outpatient care [36], increased engagement with psychiatric services at six months (but not observed at one year follow-up) [26] and reduced homelessness and psychiatric symptoms [37]. In a cost-effectiveness study from the US, the CTI was found to be cost-effective compared to the status quo for people experiencing homelessness with severe mental illness [38]. The rapid evidence assessment concluded that CTIs were effective services and, more generally, homelessness services that incorporate permanent supported housing elements are effective. The review authors identify RCTs across multiple sites and robust economic evidence (especially in the UK) as gaps in the evidence base.

In summary, there exists some high-quality evidence that CTI improves housing stability outcomes in people experiencing homelessness or groups that are vulnerably housed. The results from the same evidence base on mental health outcomes is not as strong but the majority of studies found CTI achieving favourable outcomes. Only two studies had quality of life as an outcome and only one study, from the UK, was based in a prison. That study [26] did not provide housing as part of CTI, yet provision of suitable accommodation is seen as vital for promoting successful re-integration into the community. In fact, there were only two studies, both from the US [24, 37], that provided housing as part of a CTI intervention. While those two studies suggested the intervention may be effective in improving health, the nature of the housing market and social security support provided in the UK differ markedly and therefore limit generalisability of that evidence.

For peer review only - http://bmjopen.bmj.com/site/about/guidelines.xhtml

4.1 Rationale for current pilot trial

The adverse health impacts of homelessness are compounded by the experience of imprisonment, with health outcomes substantially worse amongst people with a history of both imprisonment and homelessness than either in isolation [40]. In collaboration with the UK's leading charity for single people experiencing homelessness (Crisis), we will pilot a RCT of a housing-led CTI in four prisons, located in Wales and England. The quantitative and qualitative evidence generated will inform whether a future definitive full-scale trial of the CTI is warranted.

5 Trial objectives/endpoints and outcome measures

The overall aim is to conduct a pilot RCT in four male prisons to determine whether a future full-scale RCT assessing effectiveness and cost-effectiveness of a housing-led CTI is warranted. The research questions are:

1 **Intervention and randomised trial delivery**
- 1.1 Is the intervention acceptable to participants, those delivering and those funding?
- 1.2 What do qualitative and quantitative data suggest in terms of refinements to programme theory, implementation, fidelity, reach, scalability, and acceptability?
- 1.3 Are there potential harms and unintended consequences of the intervention? How might these be reduced? How can these be measured?
- 1.4 Are there any potential ethical, practical, statutory, or other legal barriers in running a trial in this population?

2 **Recruitment**
- 2.1 What proportion of eligible people in prison are recruited?
- 2.2 What proportion of recruited participants are retained?
- 2.3 What proportion of people in prison provide consent for linkage to routine health and criminal justice data?

3 **Data collection procedures**
- 3.1 Are methods of data collection feasible and what refinements (if any) are needed?
- 3.2 Are outcome measures suitable and what refinements (if any) are needed?
- 3.3 What consent and governance requirements are acceptable to data providers for linkage to routine health, welfare, and criminal justice data?
- 3.4 What are the likely sources of data on the health and broader social impacts of the intervention to inform an economic evaluation in a full-scale trial?

For peer review only - http://bmjopen.bmj.com/site/about/guidelines.xhtml

5.1 Primary outcomes measure(s)

The primary outcome is progression criteria that will determine whether a full-scale RCT of the intervention is warranted. The progression criteria are:

1) Recruitment: 50% of those approached agree to participate.
2) Retention: 60% of those recruited, in both intervention and control groups, are retained at final follow-up.
3) The process evaluation provides evidence that the process is acceptable for participants and staff delivering the intervention.
4) The intervention is implemented with fidelity in all settings.

The progression criteria will be agreed in collaboration with an independent Trial Steering Committee (TSC), that will carefully consider progress against these criteria using thresholds for progression according to a traffic light system (green: all criteria are met, the study should progress as designed in this pilot; amber: the majority of criteria are met and with adaptations to methods all criteria could be met; red: the minority of criteria are met and the study should not proceed).

5.2 Secondary outcomes measures

Indicative outcome measures for a full-scale trial will be evaluated and will include:

1. **Primary:** Health-related quality of life (SF-12 total score)
2. **Secondary:**
   - Health Related Quality of Life (EQ-5D)
   - Housing stability (i.e., number of days in a stable accommodation)
   - Substance use and hazardous alcohol consumption.
   - Deaths (including cause of death and hospital deaths)
   - Capability wellbeing (ICECAP-A)
3. **Exploratory:**
   - Percentage of participants who consent to link data their data anonymously with routine electronic data through SAIL, NHS England and Ministry of Justice Data Lab.
   - Percentage (of those who consented) of successfully linked records with data held in SAIL, NHS England and Ministry of Justice Data Lab to further inform additional secondary outcomes, for example:
     - Re-incarceration
     - Support networks

For peer review only - http://bmjopen.bmj.com/site/about/guidelines.xhtml

- Crime (e.g., criminal arrest, criminal convictions, police contacts, rearrest)
- Education training and employment
- Finance, benefits, and debt
- Support network/family & friend connections
- Wellbeing
- Community integration and engagement

6 Trial design and setting

This is a multicentre phase 2 pilot Randomised Control Trial (RCT) with an integrated process and economic evaluation. Eighty men leaving prison at risk of homelessness will be recruited and randomised on a 1:1 ratio (stratified by prison) to take part in a CTI or receive usual care. Sites include five male prisons (HMP Liverpool, HMP Altcourse, HMP Risley, HMP Swansea and HMP Parc) where housing-led CTI has recently been introduced and implemented in the first half of 2021. Participants will complete a survey at baseline and followed up at 3-, 6-, 9- and 12-months post randomisation. A subsample of intervention participants (n=12 (x 2 interviews)), control participants (n=16), prison staff (n=6) and interventionists (n=6) will be sampled to maximise variability (based on age, prison, caseworker, differing levels of engagement with the trial (and the intervention specifically), and phases of delivery) to take part in repeated (intervention participants only)/one-off interviews. We will additionally recruit n=12 individuals currently taking part in CTI intervention but not recruited on the trial to take part in a one-to-one interview to find out more about their experiences of the inte4rventions and thoughts on the proposed recruitment methods. Consent will be sought to link with participants routinely collected electronic health records via SAIL and NHS England as well as augmented Criminal Justice data via the Ministry of Justice DataLab.

6.1 Risk assessment

A trial Risk Assessment has been completed to identify the potential hazards/risks associated with the trial and to assess the likelihood of those hazards/risks occurring and resulting in harm. This risk assessment will include:

- The known and potential risks and benefits to human participants
- How high the risk is compared to normal standard practice.
- How the risk will be minimised/managed

This trial has been categorised as a low-risk trial, where the level of risk is comparable to the risk of standard care. A copy of the trial risk assessment may be requested from the Trial Manager.

For peer review only - http://bmjopen.bmj.com/site/about/guidelines.xhtml

7 Site and Investigator selection

This trial will be carried out at five male prisons in England and Wales (HMP Liverpool, HMP Altcourse, HMP Risley, HMP Swansea and HMP Parc) where CTI has recently been introduced and is being delivered by CTI workers employed by Crisis (housing charity).

8 Participant selection

Participants are eligible for the trial if they meet all of the following inclusion criteria and none of the exclusion criteria apply. All queries about participant eligibility should be directed to the Trial Manager before randomisation/registration. Participants are split between those involved in the RCT and those currently receiving CTI who will only be interviewed/observed.

8.1 RCT and non-RCT participants Inclusion criteria

Men leaving prison at risk of homelessness can be included if they are:

1. Aged over 18 years.
2. Released into the Swansea or Liverpool local authority areas and have a local connection (e.g., lived there previously; close relatives living in these areas)
3. Recourse to public funds
4. Have experienced prison and homelessness at least once.
5. Have mental health or substance use support needs.

8.2 RCT Exclusion criteria

Men leaving prison cannot take part if they are:

1. under the Multi-Agency Public Protection panel 3 (MAPPA 3)
2. in receipt of Housing First (i.e., support needs too high/complex to benefit from CTI)

9. Recruitment, Screening, and Registration

9.1 RCT PARTICIPANTS

9.1.1 Participant identification:

Potential participants will be referred to Crisis for eligibility screening by the Community Offender Management Team or Probation service. Crisis will screen potential participants at weekly allocation meetings to see if an individual meets the trial inclusion/exclusion criteria and will invite eligible individuals to take part in the research (using the expression of interest form which provides a summary of the study). Where participants express an interest in taking part, Crisis staff will pass on

For peer review only - http://bmjopen.bmj.com/site/about/guidelines.xhtml

their details to the research team using an Expression of Interest (EoI) from. Following this, the recruiter (North Westcoast CRN, Swansea Bay University Health Board R&D staff, PHaCT trial team) will arrange access to the prison for the baseline visit with support of the local Crisis CTI staff using the prison visitor scheme.

9.1.2 Screening logs:

will be kept by Crisis CTI staff for each site to record all referrals received regardless of eligibility status and whether they were approached/consented or not. Screening logs will capture non-identifiable information on a tally system relating to referral route, eligibility status, EoI, recruitment status, reasons for non-eligibility or non-recruitment (where possible). Logs will be completed electronically by individually designated Crisis CTI staff for each site. Where there are multiple staff recruiting at one site, entries will be aggregated onto one log. Data entered onto the log will only be accessible to named trial staff members which will be outlined in the Site Delegation Log. Logs will be returned to the Data Manager on a weekly basis using the trial email address.

9.1.3 Recruitment:

A total of 80 participants will be recruited. We will also recruit a sub-sample of intervention participants (n=12 (x 2 interviews)), control participants (n=16) prison staff and interventionists (n=12) to take part in face-to-face repeated (intervention participants only)/one-off interviews.

9.1.4 Informed consent:

During the baseline visit the potential recruit will be provided with a participant information sheet and trained recruiter will go over the details explaining the risk and benefits of taking part to the potential participant. It will be explained to them that all information they provide, including their personal details, will be kept strictly confidential and will only be accessible to the immediate research team at Cardiff University and that they have the right to withdraw from the study at any time without having to give a reason. Potential participants will be provided with sufficient time to consider their participation and will have multiple opportunities to ask any questions. We might have to share personal data with the University of Glasgow as the Sponsor of this study so that they can check we are following legal processes. This will be managed in strict confidence.

Should they agree to take part, written informed consent will only be obtained by the recruiter (who will be trained in taking informed consent) using the trial Consent Form. A Site Delegation Log will outline who is responsible for taking participants consent and will be kept centrally with the trial team. Participants will be asked to sign two copies (one copy to be kept by the participant and the other to

For peer review only - http://bmjopen.bmj.com/site/about/guidelines.xhtml

be returned to the TM via registered mail). Only once written informed consent has been obtained from the participant will they be considered a trial participant.

Participants will be asked to provide written consent to take part in a potential qualitative interview, although it will be explained to them that they may not be sampled for this aspect of the trial. It will be further explained that if they later choose not to take part in this aspect of the trial, that this will not impact on their valuable contribution made to the trial so far. They will also be asked if they are willing to consent to trial staff requesting access to their anonymised electronic routine data through a third-party data provider. Participants consent to take part will be verbally reaffirmed at each data collection point.

9.1.5 Randomisation:

Participants will be randomised to receive CTI or usual care following gaining informed consent and completion of the baseline assessment by the recruiter. Randomisation will be on a 1:1 ratio and stratified by prison, using random blocks, and generated by the trial statistician and checked by the senior statistician. Allocation will be revealed to participants by the recruiter by opening a sequential sealed envelope. and communicated to the participant by the recruiter (using a prepared script). Owing to the nature of the intervention, it will not be possible to blind participants, recruiter or CTI staff to the treatment allocation.

9.2 NON-RCT PARTICIPANTS

9.2.1 Participation identification:

Those currently receiving support from Crisis through CTI will be invited by Crisis to take part in an interview and allow observations of their meetings (maximum 3 per person).

9.2.2 Recruitment:

A maximum of 12 interviews and observations will be conducted.

9.2.3 Informed consent:

Those indicating interest will be provided a participant information sheet and PHaCT research staff will go over the details explaining the risk and benefits of taking part to the potential participant. All details of the PIS and CF will be explained with opportunities to ask any questions. Paper consent forms will be used. Participants will be asked to sign two copies (one copy to be kept by the participant and the other to be returned to Cardiff University for storage). Only once written informed consent has been obtained from the participant will they be interviewed/observed.

For peer review only - http://bmjopen.bmj.com/site/about/guidelines.xhtml

10.Withdrawal & lost to follow-up

10.1 RCT Withdrawal

During recruitment it will be explained to potential participants that they have the right to withdraw consent for participation in any aspect of the trial without having to provide a reason. Participants care will not be affected at any time by declining to participate or withdraw from the trial. However, whenever a reason is given, this will be recorded by the study team on a study withdrawal form following standard CTR processes and used anonymously for the process evaluation.

If a participant initially consents but subsequently withdraws from the trial, clear distinction must be made as to what aspect of the trial the participant is withdrawing from (i.e., partial withdrawal from questionnaire interview etc. withdrawal of all data collection, withdrawal from intervention etc.) and recorded on a withdrawal form.

10.2 RCT Strategies to minimise loss to follow up

We aim to follow-up all participants recruited into the trial. To maximise our follow-up success rate where possible we will aim to follow up participants in person and brokered via the participants probation officer. Where it's not possible to follow-up in person, we will attempt to contact participants via alternative contact details they have provided us, or updated details provided by the probation officer. We will make 4 attempts to follow-up with participant at each timepoint. Where a participant has been lost to follow-up at one time point, they will still be contacted at the next follow-up timepoint, unless they have explicitly withdrawn from follow data collection. Information regarding follow-up routes and contact attempts will be collected for the process evaluation.

10.3 NON-RCT Withdrawal

During recruitment it will be explained to potential participants that they have the right to withdraw consent for participation in any aspect of the trial without having to provide a reason. Participants' care will not be affected at any time by declining to participate or withdraw. However, whenever a reason is given, this will be recorded by the study team on a study withdrawal form following standard CTR processes and used anonymously for the process evaluation. If a participant initially consents but subsequently withdraws from the trial, we will inquire if they wish for all previously recorded data to be removed and act accordingly.

For peer review only - http://bmjopen.bmj.com/site/about/guidelines.xhtml

11.Trial Intervention

11.1 Critical Time Intervention

The Critical time Intervention (CTI) was first developed by mental health clinicians and researchers working in New York City men's shelters in the 1980s and 1990s [41]. It was noted that despite clear discharge planning, a large proportion of men experienced homelessness within 18 months of discharge from shelters [42]. The CTI approach is a psychosocial individual-level intervention that facilitates integration and continuity of care during a time of transition from institution to community settings [43]. It achieves this by ensuring that an individual has long term ties to services, family and friends through providing time-limited, but direct, emotional and practical support during the transition. CTI participants create a personal development plan and identify short-medium-long term goals depending on their individual needs, with the aim to develop self-management skills, feeling of empowerment and improving their health and quality of life. Housing provision provides additional stability, removing practical and emotional issues tied to experiencing homelessness.

Key principles of CTI delivered by Crisis:

- Designed around a transition
- Time limited
- Community-based
- Developing social connection and capital
- Linking to long-term support (but not doing)
- Harm reduction / recovery orientated
- Limited focus
- Small caseloads
- Decreasing intensity / phased approach
- Frequent case reviews
- Team based weekly supervision

The intervention involves:

a) A pre-engagement phase:

Starting before discharge, an assessment panel meeting identifies those at higher risk of homelessness. The caseworker meets the client and begins a process of engagement and relationship building. They assess the client's needs and implement a tailored (and agreed) transition plan to link the client to services and supports in the community. After the client is released from the institution, they are placed into tenancy (immediately or following a brief period in temporary accommodation).

For peer review only - http://bmjopen.bmj.com/site/about/guidelines.xhtml

b) 3 phases (lasting 3 months each):

Phase one ("Transition to the community"): an intense period of forming links and relationships and shared goal setting; aiming to improve crisis-resolution skills, provide support and advice tailored to their needs, and mediate any conflicts. This phase involves weekly home visits and other meetings with the client, with the client's caregivers, and community service providers.

Phase two ("Try out"): the caseworker monitors and adjusts the systems of support that were developed during phase one. This phase involves fewer meetings with the client, as the caseworker encourages the client to problem-solve and manage practical issues (e.g., sorting bills and general money/benefits management; living skills; social support and meaningful use of time; managing landlord relationship) with the help of community resources and family members, and intervenes only if the client is receiving inadequate support or if a crisis occurs.

Phase three ("Transfer of care"): the caseworker reduces the level of support and begins to 'step away'. They help the client to develop a plan to achieve long-term goals (e.g., employment, family reunification) and finalises the transfer of responsibilities to caregivers and community providers (i.e., the CTI intervention is terminated with support in place).

11.2 Compliance

Compliance to the intervention delivery will be measured as part of a fidelity assessment in the process evaluation using a previously developed CTI fidelity assessment tool (CTI-TS). A sample (n=24) of sessions will be observed/audio recorded for this purpose.

11.3 Training

Training will be provided for Crisis CTI staff members and recruiters on the recruitment and data collection processes. This will include trial specific training on the referral process, completion of screening logs, completion of the baseline measure and data entry on the database. A communication strategy will be developed to target Probation Officers and Community Offender Management teams to raise awareness of the trial and the referral process.

12 Trial procedures

The PHaCT trial will involve the following procedures:

1. <u>Eligibility assessment</u>

Crisis CTI staff will be informed of potential (i.e., those at risk of homelessness) recruits either by Probation Officers or the Community Offender Management team. On receiving details of potential

For peer review only - http://bmjopen.bmj.com/site/about/guidelines.xhtml

individuals, they will screen these for eligibility at their weekly meetings and where individuals are eligible they will contact them and provide them with a brief verbal explanation of the trial with support of 1 summary information sheet. Where potential recruits express interest in taking part, the Crisis CTI staff member will contact the recruiting staff (i.e CRN/R&D/study staff) and arrange for them to attend the prison for a one off visit to take informed consent and complete the baseline assessment. Should the 45-minute prison visitor slot not allow sufficient time, the recruiter may split the baseline visit in two and book a subsequent visit. Should it not be possible to arrange a face-to-face meeting (i.e., in the case of a prison lockdown), alternative arrangements will be made by the Crisis CTI team for the recruiter to speak to the individual on the phone. This route will only be used as a back-up option.

2. Baseline visit

a. Informed consent

At the baseline visit, the recruiter (if required) will provide the potential recruit with the full participant information sheet. Once a potential recruit has provided informed consent they will be regarded as a participant.

b. Baseline CRF

After gaining informed consent, the recruiter will proceed with the baseline CRF. This will be completed on paper. The participant will be given the choice to either complete this on their own if they wish, or, with the support of the recruiter. See Figure 1 for full breakdown of baseline assessments (page 28).

c. Allocation

Once the CRF has been completed the recruiter will proceed to reveal the allocation to the participant. Sealed envelopes containing the series of randomised allocations will be pre-prepared by trial statisticians for each prison and given to recruitment staff. Envelopes will be sequentially numbered, and the recruiter will be required to open the next envelope in the sequence. The contents of the envelope will state either 'Usual care' or 'Critical Time Intervention', along with the participants unique participant identification number (PID). The recruiter will then verbally explain (with use of a script), which group they have been allocated to and what the next step is for them (i.e., Crisis to make contact or follow usual prison procedure). The participant will have further opportunities to ask any questions. The recruiter will then make a note of the participants PID number on the completed CRF and consent from and file these in separate concealed folders/envelopes.

3. Data entry

For peer review only - http://bmjopen.bmj.com/site/about/guidelines.xhtml

Within 48 hours of returning to the office, the recruiting staff will be required to enter the data on the bespoke online trial database and the paper CRF and consent form will be sent to CTR via registered mail for review and storage. Once the participants details and CRF responses have been entered the PHaCT trial team will receive an email notification of a new participant entry. This will be monitored by the Data Manager and where an individual has been randomised to the intervention arm, they will then inform the Crisis CTI team of the participants allocation. If recruitment is conducted by a trial team staff member, they will input the data after collection within 48 hours of collection.

4. CTI Intervention

Those participants who have been randomised to the intervention group will then be contacted by the Crisis CTI staff to proceed with the first stages of the intervention.

5. Follow-up CRFs at 3-, 6-, 9- and 12-month

All participants will be contacted to complete their follow-up assessments. Every effort will be made to complete all follow-ups in person with the same recruiter to aid in building rapport and maximising retention. However, where this is not possible, phone contact will be attempted by the research team. Where follow-up data collection is conducted in person the data will be collected on paper CRFs and where participants are contacted by phone (back-up option) data will be directly entered on the database. Follow-up visits will try to be arranged through the support of probation officers to maximise retention. At each follow-up assessment, the researcher will reaffirm their verbal consent to take part before completing the survey. Where it has not been possible to contact an individual after four attempts, they will be considered lost to follow-up at that timepoint. They will be contacted again for the next follow-up timepoint. Within 48 hours of returning to the office, the recruiter will be required to enter the data on the bespoke online trial database for each follow-up point and the paper CRF will be sent to CTR via tracked mail for review and storage.

6. Interviews (RCT participants, staff/interventionists, non-RCT participants)

a. RCT Participant interviews

Of the participants who have consented to be interviewed, 12 intervention participants and 16 control participants will be sampled to take part in one-to-one semi-structured interviews with a member of the research team. Intervention participants will take part in repeated interviews split across 2 timepoints with the first interview conducted after phase 1 of the intervention and the 2nd interview after phase 2 of the intervention (total 24 interviews), however these will be augmented if required. Phase 1 interviews will focus on the recruitment methods and phase 2 interviews will focus on experiences of the intervention. Control participant interviews will take place after the final data

For peer review only - http://bmjopen.bmj.com/site/about/guidelines.xhtml

collection timepoint at 12 months and will focus on participants experiences of taking part in the trial. Interviews will be arranged at a time and suitable place (i.e., at their probation officer meeting/phone call) for the participant to maximise retention and could be over the phone or in person. Before starting the interview, the researcher will explain what will happen and reaffirm their consent to take part. The interview will be audio recorded using an encrypted audio recorder.

b. Staff/interventionist interviews

Key prison/probation staff members and intervention staff (n=12) will be approached to take part in a one-to-one semi-structured interview with a member of the research team. Interviews will be arranged at a suitable time and place for the individual and where possible will be conducted face-to-face with phone interviews as a back-up option. Staff/interventionists will be provided with a separate information sheet and will be asked to provide written informed consent. The interview will be audio recorded using an encrypted audio recorder.

c. Non-RCT participant interviews

Individuals currently receiving CTI from Crisis (n=12) will be approached to take part in an interview to assess the intervention. These will be one-to-one semi-structured interviews with a member of the research team. Interviews will be arranged at a suitable time and place for the individual and where possible will be conducted face-to-face in the Crisis offices with phone interviews as a back-up option. Non-RCT participants will be provided with a separate information sheet and will be asked to provide written informed consent. The interview will be audio recorded using an encrypted audio recorder.

7. Session observations/audio recordings

A sample of RCT participants (n=24) and non-RCT participants (n=12) of intervention sessions will be observed (maximum of 3 per person) where possible or audio recorded as a back-up to gain an insight into the delivery of the intervention. This will aid in the fidelity assessment of the intervention. Observations/audio recordings will be split across the different phases of the intervention. A fidelity assessment tool will be developed to support the observation data collection. Where sessions are audio recorded, research staff will use an encrypted recorder.

8. Routine Data

Where participants have consented to have their electronic routine data linked with health and/or criminal justice data, their details (i.e., name, address, gender, date of birth and NHS number where possible) will be sent to the relevant data providers (NHS England, SAIL and MoJ Criminal Justice dataLab) by the research team using a secure system (i.e., portal or secure email account) for linking.

For peer review only - http://bmjopen.bmj.com/site/about/guidelines.xhtml

12.1 Assessments

Figure 1. Schedule of enrolment, interventions and assessments[1]

Procedures	Data collection timepoints							
			Follow Up				Routine data	Rolling
	Screening	Baseline	3 month	6 month	9 month	12 month	(SAIL/NHS England/MoJ DataLab)	CTI & Usual care costs
Eligibility assessment	X	X						
Expression of interest	X							
Informed consent		X						
Affirmed consent			X	X	X	X		
Demographics		X						
Randomisation		X						
Delivery of intervention		X	X	X	X			
SF-12		x	x	x	x	X		
EQ-5D		x	x	x	x	x		
ICECAP-A		X	x	x	x	x		
AUDIT-C		X	x	x	x	x		
Housing		X	x	x	x	x		
Resource use questionnaire		X	x	x	x	x	x	

[1] Taken from the HRA CTIMP protocol template (2016).

For peer review only

For peer review only - http://bmjopen.bmj.com/site/about/guidelines.xhtml

Cost log (intervention and control)								X
Criminal Justice							x	
Participant interviews			Phase 1	Phase 2		Control		
Staff interviews			x	x	x	x		
Interventionist interviews			X	x	x	x		
Intervention observations & Fidelity assessment			x	x	x			
Safeguarding		X	X	X	X	X		
Withdrawal Checklist		X	X	X	X	X		

12.2 Follow-up

Participants will be followed up at 3- 6- 9- and 12-months post randomisation. This frequency of follow-up is used in RCTs with people experiencing homelessness to facilitate tracking of what can be a transitory population [44]. Follow-ups will be conducted in person wherever possible through the support of probation officer (i.e., at regular probation meetings). Four attempts will be made to follow participants up at each timepoint and where it has not been possible to follow them up they will be considered to be loss to follow-up at that timepoint. The team will continue to follow-up participants at all remaining timepoints.

13 Safety reporting

We do not anticipate there to be any serious adverse events as a result of taking part of this trial or which can be attributed to the intervention. However, as this is a vulnerable population, they may experience some levels of distress due to their current situation which they may share with staff. For

For peer review only - http://bmjopen.bmj.com/site/about/guidelines.xhtml

this reason, we will manage and record any safeguarding issues we become aware of during data collection and where possible during intervention delivery. To do this, we will include a safeguarding section in our participant information sheet and data collection forms where we will provide a number of contacts and links where individuals can seek additional support should they require this. Where there are any serious concerns, i.e., risk of harm or death to self or others, these will be reported immediately upon become aware of them to the Chief Investigator and escalated accordingly depending on the nature of the concerns (i.e., calling 999 where relevant or reporting these to the participants probation officers). Consent to escalate these will be collected. To keep a record of any safeguarding concerns, a 'safeguarding form' will be completed by the trial team/CRN/R&D staff within 24 hours of becoming aware of the concerns. Reported safeguarding concerns will be reviewed at monthly TMG meetings and signed off by the Chief investigator. Regular debriefing sessions will be held with the recruiters who are involved with data collection to ensure they are well supported. Where any face-to-face data collection will be conducted (i.e., for baseline recruitment, follow-up data collection, interviews or observations) these will take place in suitable public venues and no home data collection will take place. Data collection in the prison will be completed by named recruiter and will follow the visitor system whereby they will be escorted and accompanied by prison staff key holders/Crisis staff.

Safeguarding concerns may include:

- Participants/others safety
- Participants declined mental health
- Research staff safety
- Research staff wellbeing

For any lone working during the qualitative data collection we will follow the guidelines set out in the SOP on lone working (SOP/003/12) to ensure safety of researchers.

13.1 Definitions

Term	Definition
Safeguarding concern	Any issue that includes but is not necessarily limited to:<br>• Self-reported immediate self-harm or suicide plans<br>• Self-reported immediate plans to harm others

13.2 Reporting process

1. Within 24 hours of being made aware of safeguarding concern this should be reported to the trial team.
2. Where possible (i.e., access to the trial database) the individuals should directly complete a safeguarding form, however if reported by the Crisis CTI team, this should be reported to the trail team by phoning or emailing the trial administrator. No identifiable information should be used and participants should only be referred to by their PID number.
3. The trial team will review/enter details on the safeguarding form. Where external input is needed, i.e., probation officer etc. this will be sought.
4. Safeguarding forms will be reviewed by the Chief Investigator and signed off once appropriately resolved.
5. Safeguarding data will be collated onto a log by the Data Manager and reviewed at monthly TMG meetings.

14 Statistical considerations

14.1 Randomisation

Randomisation will be completed by a trial statistician on a 1:1 ratio and stratified by prison, using random blocks. A randomisation protocol will be developed separately and will include testing of the process and the system. Due to the restrictions of using electronic equipment during recruitment, randomisation will be completed using concealed paper envelopes. Each site will be provided with a box containing 60 sealed sequentially numbered envelopes (total 4 boxes). Envelopes will contain the arm allocation and PID number. Boxes and all envelopes will be clearly labelled with the prison name and sequence number. Training will be provided to site staff to outline the randomisation method. Prepared randomisation boxes will be checked by a member of the trial team to ensure the correct sequence is followed before being sent to sites. Randomisation method will be clearly documented in the randomisation protocol. Randomisation will be checked by TM or DM to ensure correct allocation.

14.2 Blinding

Allocations will be revealed after the baseline assessment, therefore at baseline participants and staff will be blind to group allocation. However, once randomised it will not be possible for participants or staff members to be blind as they will either receive the intervention or receive usual care. However, the trial statistician and health economist will remain blinded to group allocation during analysis. It will not be possible to blind the qualitative researcher.

For peer review only - http://bmjopen.bmj.com/site/about/guidelines.xhtml

14.3 Sample size

No power calculations have been performed as our aim is to evaluate acceptability of the intervention, not to estimate effectiveness. This study will determine rates of recruitment, response and retention, and the distribution of the indicative primary outcome measures in a full-scale RCT, to inform a full-scale trial sample size calculation. Given there is capacity to deliver the intervention to 120 participants per year, we aim to recruit 80 participants (40 per arm) which allows us to estimate a conservative recruitment rate of 50% within a 95% confidence interval (CI) of ±11% (75% rate ±9.5%).

14.4 Missing, unused & spurious data

The rate of completeness for baseline and follow-up measures will be described. No imputation will be performed.

14.5 Procedures for reporting deviation(s) from the original SAP

Any deviations from the original statistical analysis plan (SAP) will be submitted as substantial amendments where applicable and recorded in subsequent versions of the protocol and SAP.

14.6 Termination of the trial

There will be no formal 'stopping rules' or 'discontinuation criteria' for individual participants, parts of study and entire study.

14.7 Inclusion in analysis

The trial population will be all participants who are eligible and consent to participate in the trial.

15 Analysis

15.1 Main analysis

The reporting of findings will be in accordance with the CONSORT guidelines for pilot RCTs [47]. The primary analysis of the pilot RCT will determine whether the prespecified progression criteria to a full-scale trial are met. All outcomes related to feasibility will be reported as point estimates with 95% confidence intervals (CIs).

Recruitment, baseline response, randomisation, follow-up at each time point, and consent to routine data linkage will be summarised in a CONSORT flow diagram. We will tabulate demographic characteristics of participants by study arm using descriptive statistics: means and standard deviations (SDs)(or medians and interquartile (IQR) ranges, as appropriate) for continuous outcomes, frequencies and percentages for categorical outcomes.

For peer review only - http://bmjopen.bmj.com/site/about/guidelines.xhtml

We will examine rates of completion of outcomes at each follow-up time point. We will pilot the analyses of effectiveness outcomes that would be done in a full-scale trial. Each outcome will be summarised at each time point (number (%), mean (SD), median (IQR)) and will be analysed in a separate multi-level regression model following the intention to treat approach and using data at baseline and follow up. The model will include fixed effects for intervention group, assessment time point (3, 6, 9, 12 months) and an interaction term between group and time. The model will allow us to examine the change in intervention over time compared to changes in the control arm. These estimates (odds ratios, mean differences) will be adjusted for participants nested within prison and will be presented alongside 95% CIs.

As this is a pilot trial, not powered for effectiveness, no hypothesis testing will be performed and no p-values presented. We will also estimate the clustering of outcomes within prisons and partial clustering of prisoners those imprisoning caseworkers (CTI arm only) by estimating intra-cluster correlation coefficients (with 95% CIs). Full details of the statistical analysis will be detailed in the statistical analysis plan (SAP). All analyses will be performed in Stata or R.

15.1.1 Sub-group & interim analysis

No sub-group analyses are planned.

15.2 Qualitative analysis

All interviews will be transcribed verbatim (audio recordings will be uploaded and transcribed by an approved Cardiff University supplier via a secure portal for the purpose of transcription and deleted from the portal once transcription is complete) and analysed using Thematic Analysis [48]. A coding framework will be developed which will be closely aligned with the trial specific process evaluation framework and data will be categorised accordingly. Commonly expressed themes as well as unusual cases will be identified. Intervention delivery observations/audio recordings will be coded against key intervention components to assess fidelity of the intervention delivery. All interview transcripts will be checked for accuracy/anonymisation and 20% will be double-coded to check for reliability of the coding framework.

The qualitative analysis will then feed into the overall process evaluation which will be conducted in accordance with the MRC guidance [45] and will use a mixed methods approach. The aim of the process evaluation will be to report on each of the process evaluation components (Reach, Recruitment and Retention, Fidelity and Acceptability, Compliance, Contamination and Context) [45]. Fidelity will be assessed with support from a previously developed CTI fidelity assessment tool (CTI-TS) [46]. Data sources will be mapped against each of these components and data will be triangulated

For peer review only - http://bmjopen.bmj.com/site/about/guidelines.xhtml

(where relevant) to provide a detailed overview of participants and staff experiences and acceptability of the intervention as well as the delivery methods and fidelity of delivery.

This analysis will ultimately inform understanding of acceptability, views and experiences of the intervention and acceptability of trial, to inform acceptability of a full-scale trial.

15.3 Economic evaluation

The broader health and social impacts of the intervention, and associated costs will be considered to inform an economic evaluation in a full-scale trial. In particular, we will: test the feasibility of data collection instruments in capturing costs associated with participants' short-term use of health and community services and broader costs (e.g., criminal justice, welfare costs); test feasibility of using cost logs to capture the costs to deliver the intervention (e.g., staff costs); examine the distribution of the health-related quality of life and capability measures (EQ-5D and ICECAP-A has previously been successfully used in the people experiencing homelessness population [5, 6, 49]) and measure its association with housing stability, physical and mental health. Descriptive statistics (including levels of missing data) for each outcome and cost variable will be reported at each data collection time point and for each intervention group. The patterning and percentage of missing data will inform likely analytical strategy in full-scale trial (e.g., whether multiple imputation is necessary). The distribution of outcome and cost variables will inform likely distributional assumptions to be made when propagating uncertainty through the cost-effectiveness analyses.

An exploratory cost-consequences analysis [50] will report costs alongside consequences for intervention and control groups (this will involve participation of PPI representatives to ensure all relevant costs and consequences are captured). Although cost-effectiveness analyses will be considerably uncertain at this pilot trial stage, we will conduct a short-term (1 year time horizon) cost-utility analysis. Kaplan-Meier estimates of survival probabilities will be combined with the repeated measurements of costs and EQ-5D to obtain mean costs and quality adjusted life years (QALYs) for both intervention and control groups (allowing estimation of the incremental cost-effectiveness ratio) [51]. Scenario sensitivity analysis [52] will be used to identify prices of the CTI intervention at which it would be cost-effective (using NICE threshold) given a range of plausible short-term QALY gains. We will use the cost-utility analysis results to inform value of information (VOI) [53] analyses. These VOI analyses will also assist in identifying the likely key parameters that will drive decision uncertainty and therefore help in planning the design of the full-scale trial.

A review of existing evidence will assist in: 1) identifying suitable modelling frameworks to assess the cost-effectiveness of the CTI intervention over the long-term; 2) identifying the key sources of

For peer review only - http://bmjopen.bmj.com/site/about/guidelines.xhtml

uncertainty in relation to the probability of the intervention being cost-effective when a long-term time horizon is used. This review and the pilot economic evaluation outlined above will be used in combination to develop an economic conceptual model [54] for the long-term, to be developed at full-scale trial stage. The process evaluation, cost consequences analysis and cost-utility analysis in this pilot trial will inform the optimal time horizon for the economic evaluation alongside the full-scale trial. We will explore using the linked data sources (see section 15.4) to inform longer-term cost-effectiveness modelling in the full-scale trial stage.

15.4 Routine data analysis

We will review existing routine data sources that could be used to assess outcomes at the main stage trial. Provisional outcomes of interest (see logic model in Appendices) include housing stability, criminal justice contacts, alcohol and substance use, and mental health. We will attempt to harmonize potential data sources so that similar outcomes can be measures from across the devolved UK nations. based on this review, data owners will be contacted to assess whether data are accessible for linkage-based studies, and any practical and financial implications if accessing these data. We will also work with data owners to develop trial materials, such as consent questions and participant information sheets, which meet their requirements to enable access to data for the main stage trial without the need for these to be further developed. The review will also seek to explore potential codes within data sources that align with measurable outcomes of interest, i.e., diagnosis codes for hospitalisations due to substance use.

In tandem with this data exploration and engagement activity, we will also be conducting test data linkages to healthcare data sets in England and Wales, and criminal justice data. Test linkages will allow us to assess the percentage of participants who consent to have their data linked and the percentage of data records which can be linked successfully with these data providers. We will generate summary statistics of the completeness of data needed to undertake linkage, i.e., percentage of people providing personal details. As each data owner uses different data matching methods when extracting records, information on the proportion of people from the original trial cohort who we are able to link to will aid in assessing the extent to which linkage at a full trial will generate samples with enough power to detect intervention effects.

De-identified healthcare data will be accessed via NHS England and the Secure Anonymised Information Linkage (SAIL) databank, in England and Wales respectively. Applications to access healthcare data will be made to NHS England via the Data Access Request Service and to SAIL via their Information Governance Review Panel. Information requested from NHS England and SAIL will be

For peer review only - http://bmjopen.bmj.com/site/about/guidelines.xhtml

limited for the purposes of a test linkage to records related to periods spent in hospital, use of accident and emergency services, outpatients' services, and death records [55]. We will request data from 5-years prior to enrolment in the study, to assess the potential of linkage to develop baseline healthcare use estimates, up to the latest data available, to assess the use of linkage to evaluate difference in health outcomes between study arms.

We will also seek to link to criminal justice data via the Ministry of Justice Data Lab ('the Lab'). The Lab is a service operated by the Ministry of Justice that links details of clients engaging with third sector organisations and provides summary reports of re-offending rates for clients–compared to a matched comparison group that the Lab generates form the wider prisoner population. As the Lab's services may conflict with trial procedures, such as having two trial study arms and the requirement that summary reports be published on the MoJ website, the test linkage to criminal justice data may not occur if deemed incompatible with study goals.

To link records, personal details will need to be shared with data owners, including name, date of birth, gender, and postcode. It may help data owners if unique identification numbers used by their computer systems are provided; therefore, Police National Computer number and NHS number will also be collected and shared with the appropriate data owner. We will use consent questions and participant information materials based on previous studies from the CTR.

The trial Data Manager will be responsible for the processing of personal data prior to sending it to relevant data owners. Only pre-approved secure methods will be used to transfer personal data between the CTR and data owners. Trial researchers will have access to either aggregated data reports (in the case of the MoJ Data Lab) or de-identified data which will be accessed via a secure gateway (SAIL Gateway)—see data flow diagrams in Appendices.

16 Data Management

A Data Management Plan (DMP) and Qualitative data collection and Analysis Plan (QAP) will be developed to outline the different types of data being collected, collection methods, processing of data, data storage and data sharing. The DMP will include details of the management of the following source data:

Trial data	*Source data*

For peer review only - http://bmjopen.bmj.com/site/about/guidelines.xhtml

	Screening	CRF	Electronic routine health data	Aggregated criminal justice data	Participant interviews	Staff interviews	Observational notes/audio recordings	Safeguarding	Withdrawal
Recruitment	*x*				*X*	*X*			
Health		*X*	*X*		*X*	*X*			
Mental health		*X*	*X*		*X*	*X*			
Substance misuse		*x*	*x*		*X*	*X*			
Police & MoJ contact				*x*	*X*	*X*			
Intervention Engagement					*X*	*x*	*x*		
Intervention content delivery					*X*	*X*	*X*		
Safeguarding								*X*	
Withdrawal									*x*

Source Data is defined as *"All information in original records and certified copies of original records of clinical findings, observations or other activities in a trial necessary for the reconstruction and evaluation of the trial. Source data are contained in source documents."* There is only one set of source data at any time for any data element, as defined in site source data agreement.

16.1 Data collection

Data will be collection directly from participants, staff and through secure data providers. Data will be collected either via paper, electronic, audio recordings or observation notes form the intervention delivery. Any paper based data collection will be securely transported and entered on the trial database on return to the office, personal details will be shared securely with data providers, audio

For peer review only - http://bmjopen.bmj.com/site/about/guidelines.xhtml

recordings will be uploaded to a secure portal for secure transcription and any hand written observational notes will be word processed as soon as possible.

stor16.2 Completion of CRFs

16.2.1 Paper CRFs

Data will be collected via specifically designed trial paper CRFs. Paper CRFs will be designed with support from the trial PPI lead to sure it is easy to follow. The recruiter (CRN/R&D staff/research team) will securely transport CRFs. Once the recruiters return to the office they will enter the data directly onto the trial database. This will reduce any unnecessary data queries. Once the data has been entered, paper CRFs will be stored in the site file and later returned to the trial team in registered post. CRF pages and data received by the trial team from participating trial sites will then be checked against the database for missing, illegible or unusual values (range checks) and consistency over time. If missing or questionable data are identified, a data query will be raised on a data clarification form. The data clarification form will be sent to the relevant participating site. The site shall be requested to respond to the data query on the data clarification form. The case report form pages should not be altered. All answered data queries and corrections should be signed off and dated by a delegated member of staff at the relevant participating site. Where common queries or errors arise on data entry these will be entered onto a self-evident correction log with agreement from TMG members. The completed data clarification form should be returned to the trial team and a copy retained at the site along with the participants' CRFs. The trial team will send reminders for any overdue data. It is the site's responsibility to submit complete and accurate data in timely manner.

16.2.2 Electronic CRFs

An electronic bespoke database will be developed using Qualtrics to capture data collected via CRFs, withdrawal and any safeguarding concerns. This is a secure encrypted system and will be hosted on the secure Cardiff University server accessed through a password protected website and only named individuals (outlined on a trial and site delegation log) will have access. All individuals who have access will be trained in Good Clinical Practice (GCP) and data handling. A training log will keep record of this. Electronic data entry will be quality controlled through the use of appropriate skips and parameters and the system will be tested prior to the trial opening for recruitment.

17 Protocol/GCP non-compliance

Any reports of any non-compliance to the trial protocol or the conditions and principles of Good Clinical Practice need to be reported to the Chief Investigator and Trial Manager in writing as soon as they become aware of it. All serious non-compliances to be reported to the Sponsor representative.

For peer review only - http://bmjopen.bmj.com/site/about/guidelines.xhtml

18 End of Trial definition

The end of the trial is defined as the date of final data capture to meet the trial endpoints. In this case end of trial is defined as the 12 months-post randomisation follow-up timepoint.

The trial team will notify the REC of the end of the trial within 90 days of its completion or within 15 days if the trial is terminated early.

19 Archiving

The Trial Master File (TMF) containing essential documents will be archived at an approved external storage facility for a minimum of 15 years. The CTR will archive the TMF on behalf of the Sponsor according to the CTR Archiving Standard Operating Procedure. Any data shared with and held at University of Glasgow for the purposes of the health economic analysis will be stored securely according with University of Glasgow policy for a minimum of 10 years.

20 Regulatory Considerations

20.1 Ethical and governance approval

This protocol has approval by the sponsor (University of Glasgow) and Wales Research Ethics Committee (REC) 3 that is legally "recognised" by the United Kingdom Ethics Committee Authority for review and approval.

This trial protocol will be submitted through the relevant permission system for global governance review.

Approval will be obtained from each prison/Northwest CRN/Swansea Bay University Health Board R&D who will consider local governance requirements and site feasibility. The Research Governance approval of the host organisation will be obtained before recruitment of participants commences.

20.2 Data Protection

The trial team will act to preserve participant confidentiality and will not disclose or reproduce any information by which participants could be identified, except where specific consent is obtained. Data will be stored in a secure manner and will be registered in accordance with the UK General Data Protection Regulation. The independent data controllers for this trial are the University of Glasgow and Cardiff University.

For peer review only - http://bmjopen.bmj.com/site/about/guidelines.xhtml

20.2.1 Data sharing

Data will be shared with the University of Glasgow for the purpose of conducting the Health Economic analysis. De-identified data may be made available following review against the CTR policies on data sharing and with an agreed/signed data sharing agreement.

20.3 Indemnity

The Sponsor has appropriate insurance to indemnify the site against claims arising from the acts and/or omissions of the Sponsor or its employees in connection with the Trial (including the design of the Protocol to the extent that the Protocol was designed solely by the Sponsor and co-investigators and that the Site has adhered to the approved version of the Protocol) save to the extent that any such claim is the result of negligence on the part of the Site or its employees.

20.4 Trial sponsorship

University of Glasgow will act as Sponsor for the trial. Delegated responsibilities will be assigned to the Centre for Trials Research (CTR), Cardiff University in order to manage and run the trial and detailed responsibilities will be outlined in the Roles and Responsibilities Log.

20.5 Funding

The Trial has been funded by the National Institute of Healthcare Research (NIHR) Ref: NIHR134281 with a start date of 1st June 2022.

21 Trial management

21.1 TMG (Trial Management Group)

The TMG will meet on a monthly basis online with video conferencing details provided. Face to face meetings will be arranged throughout the duration of the trial, with the option of these being hybrid for those wishing to attend virtually instead. Meeting documentation will be sent via email one week prior to each meeting to allow members time to read any required materials. TMG members will be required to input into the study developments throughout all stages. TMG members will be required to sign up to the remit and conditions as set out in the TMG Charter.

21.2 TSC (Trial Steering Committee)

The TSC will meet on 3 occasions or more often if deemed necessary. The TSC will consist of external independent experts within prison or homelessness fields and will also include a trial statistician. The TSC will have oversight of the trial development and will support the development of the trial in any which way deemed appropriate. They will additionally take on the responsibility of the Data Monitoring Committee (DMC).

For peer review only - http://bmjopen.bmj.com/site/about/guidelines.xhtml

TSC members will be required to sign up to the remit and conditions as set out in the TSC and DMC Charters.

22 Quality Control and Assurance

22.1 Monitoring

The trial risk assessment has been used to determine the intensity and focus of central and on-site monitoring activity in the PHaCT trial. Low monitoring levels will be employed and are fully documented in the trial monitoring plan. This will include monitoring of the trial screening/recruitment process, randomisation and PID allocation as well as monitoring of data entry. Investigators should agree to allow trial related monitoring, including audits and regulatory inspections, by providing direct access to source data/documents as required. Participant consent for this will be obtained.

Findings generated from on-site and central monitoring will be shared with the Sponsor, Chief Investigator, & NW CRN/ Swansea Bay University Health board R&D.

22.2 Audits & inspections

The trial may be audited and inspected by the University of Glasgow under their remit as Sponsor. The study may be audited by NHS England Audit Team.

23 Publication policy

A publication policy will be developed in line with CTR processes. This will outline planned publications and presentations, targeted journals and conferences and writing groups. All publications and presentations relating to the trial will be authorised by the Trial Management Group. All appropriate acknowledgment statements will be used for any publications.

24 Milestones

A study Gantt will be used to keep track of key study timelines. This will be reviewed at regular meetings and kept up to date by the trial manager.

For peer review only - http://bmjopen.bmj.com/site/about/guidelines.xhtml

25 References

1. B. Volker, R. De Cuyper, G. Mollenhorst, A. Dirkzwager, P. van der Laan, and P. Nieuwbeerta, "Changes in the social networks of prisoners: A comparison of their networks before and after imprisonment," Social Networks, vol. 47, pp. 47-58, 2016.
2. S. Fazel, J. R. Geddes, and M. Kushel, "The health of homeless people in highincome countries: descriptive epidemiology, health consequences, and clinical and policy recommendations," The Lancet, vol. 384, no. 9953, pp. 1529-1540, 2014.
3. P. Goering, G. Tolomiczenko, T. Sheldon, K. Boydell, and D. Wasylenki, "Characteristics of persons who are homeless for the first time," Psychiatric Services, vol. 53, no. 11, pp. 1472-1474, 2002.
4. M. B. Kushel, S. Perry, D. Bangsberg, R. Clark, and A. R. Moss, "Emergency department use among the homeless and marginally housed: results from a community based study," American journal of public health, vol. 92, no. 5, pp. 778-784, 2002.
5. D. Lewer et al., "Health-related quality of life and prevalence of six chronic diseases in homeless and housed people: a cross-sectional study in London and Birmingham, England," BMJ Open, vol. 9, no. 4, p. e025192, 2019.
6. S. Sun, R. Irestig, B. Burström, U. Beijer, and K. Burström, "Health-related quality of life (EQ-5D) among homeless persons compared to a general population sample in Stockholm County, 2006," Scandinavian journal of public health, vol. 40, no. 2, pp. 115-125, 2012.
7. R. W. Aldridge et al., "Morbidity and mortality in homeless individuals, prisoners, sex workers, and individuals with substance use disorders in high-income countries: a systematic review and meta-analysis," The Lancet, vol. 391, no. 10117, pp. 241-250, 2018.
8. S. W. Hwang, E. Gogosis, C. Chambers, J. R. Dunn, J. S. Hoch, and T. Aubry, "Health status, quality of life, residential stability, substance use, and health care utilization among adults applying to a supportive housing program," Journal of Urban Health, vol. 88, no. 6, pp. 1076-1090, 2011.
9. J. Travis, A. L. Solomon, and M. Waul, "From prison to home: The dimensions and consequences of prisoner reentry," 2001.
10. D. A. Snow, S. G. Baker, and L. Anderson, "Criminality and homeless men: An empirical assessment," Social Problems, vol. 36, no. 5, pp. 532-549, 1989.
11. K. Reeve, "The hidden truth about homelessness," 2011.

For peer review only - http://bmjopen.bmj.com/site/about/guidelines.xhtml

12. D. Dixon, "Economic benefits of supporting homeless young people," Sykes H Youth Homelessness,. Courage and Hope, Melbourne University Press, Melbourne, 1993.
13. N. Pleace, "At what cost? An estimation of the financial costs of single homelessness in the UK," Crisis, July, 2015.
14. P. T. Mackie, et al., "Nations apart? Experiences of single homeless people across Great Britain. London: Crisis." ed, 2014. https://www.crisis.org.uk/endinghomelessness/homelessness-knowledge-hub/types-of-homelessness/nations-apartexperiences-of-single-homeless-people-across-great-britain-2014/
15. "Offender Rehabilitation Act 2014." The National Archives. https://www.legislation.gov.uk/ukpga/2014/11/contents/enacted
16. National Pathway for Homelessness Services to Children, Young People and Adults in the Secure Estate. 2015 [Online] https://gov.wales/sites/default/files/publications/2019-03/homelessness-services-for-children-young-people-and-adults-in-the-secure-estate_0.pdf
17. "Homelessness Reduction Act 2017." The National Archives. https://www.legislation.gov.uk/ukpga/2017/13/contents/enacted
18. Her Majesty's Inspectorate of Probation, "Accommodation and support for adult offenders in the community and on release from prison in England," 2020.
19. D. Ponka et al., "The effectiveness of case management interventions for the homeless, vulnerably housed and persons with lived experience: A systematic review," (in eng), no. 1932-6203 (Electronic), 2020.
20. J. Draine and D. B. Herman, "Critical time intervention for reentry from prison for persons with mental illness," (in eng), no. 1075-2730 (Print), 2007.
21. D. B. Herman, S. Conover, P. Gorroochurn, K. Hinterland, L. Hoepner, and E. S. Susser, "Randomized trial of critical time intervention to prevent homelessness after hospital discharge," Psychiatric services, vol. 62, no. 7, pp. 713-719, 2011.
22. E. Susser, E. Valencia, S. Conover, A. Felix, W.-Y. Tsai, and R. J. Wyatt, "Preventing recurrent homelessness among mentally ill men: a" critical time" intervention after discharge from a shelter," American Journal of Public Health, vol. 87, no. 2, pp. 256-262, 1997.
23. W. J. Kasprow and R. A. Rosenheck, "Outcomes of critical time intervention case management of homeless veterans after psychiatric hospitalization," Psychiatric Services, vol. 58, no. 7, pp. 929-935, 2007.

For peer review only

24. M. Shinn, J. Samuels, S. N. Fischer, A. Thompkins, and P. J. Fowler, "Longitudinal Impact of a Family Critical Time Intervention on Children in High-Risk Families Experiencing Homelessness: A Randomized Trial," (in eng), no. 1573-2770 (Electronic), 2015.
25. R. Bai, C. Collins, R. Fischer, and D. Crampton, "Family critical time intervention with housing unstable, child welfare-involved families: Service providers' and families' experiences with the phases," Children and Youth Services Review, vol. 119, p. 105592, 2020/12/01/ 2020, doi: https://doi.org/10.1016/j.childyouth.2020.105592.
26. J. Shaw et al., "Critical time Intervention for Severely mentally ill Prisoners (CrISP): a randomised controlled trial," 2017.
27. C. Lennox et al., "Critical Time Intervention: a qualitative study of the perspectives of prisoners and staff," The Journal of Forensic Psychiatry & Psychology, vol. 31, no. 1, pp. 76-89, 2020/01/02 2020, doi: 10.1080/14789949.2019.1665699.
28. D.-Y. Kim and S. M. Gayadeen, "Use of Critical Time Intervention (CTI) to Promote Continuity of Reintegration After Incarceration," Criminal Justice Review, vol. 46, no. 3, pp. 281-303, 2021/09/01 2020, doi: 10.1177/0734016820958139.
29. A. J. Baxter, E. J. Tweed, S. V. Katikireddi, and H. Thomson, "Effects of Housing First approaches on health and well-being of adults who are homeless or at risk of homelessness: systematic review and meta-analysis of randomised controlled trials," J Epidemiol Community Health, vol. 73, no. 5, pp. 379-387, 2019.
30. R. de Vet, S. N. van Luijtelaar Mj Fau - Brilleslijper-Kater, W. Brilleslijper-Kater Sn Fau - Vanderplasschen, M. D. Vanderplasschen W Fau - Beijersbergen, J. R. L. M. Beijersbergen Md Fau - Wolf, and J. R. Wolf, "Effectiveness of case management for homeless persons: a systematic review," (in eng), no. 1541-0048 (Electronic), 2013.
31. D. Herman, A. Opler L Fau - Felix, E. Felix A Fau - Valencia, R. J. Valencia E Fau-Wyatt, E. Wyatt Rj Fau - Susser, and E. Susser, "A critical time intervention with mentally ill homeless men: impact on psychiatric symptoms," (in eng), no. 0022-3018 (Print), 2000.
32. R. de Vet et al., "Critical time intervention for homeless people making the transition to community living: a randomized controlled trial," American journal of community psychology, vol. 60, no. 1-2, pp. 175-186, 2017.
33. D. A. M. Lako et al., "The effectiveness of critical time intervention for abused women leaving women's shelters: a randomized controlled trial," (in eng), Int J Public Health, vol. 63, no. 4, pp. 513-523, 2018, doi: 10.1007/s00038-017-1067-1.

For peer review only

Cardiff University Centre for Trials Research; University of Glasgow; Heriot Watt University; Prifysgol Glyndŵr Wrecsam; Wrexham Glyndŵr University; PHaCT
Funded by NIHR | National Institute for Health and Care Research; Centre for Homelessness Impact; Crisis Together we will end homelessness

34. J. Samuels, P. J. Fowler, A. Ault-Brutus, D.-I. Tang, and K. Marcal, "Time-limited case management for homeless mothers with mental health problems: Effects on maternal mental health," Journal of the Society for Social Work and Research, vol. 6, no. 4, pp. 515-539, 2015.
35. A. Tomita and D. B. Herman, "The impact of critical time intervention in reducing psychiatric rehospitalization after hospital discharge," (in eng), Psychiatr Serv, vol. 63, no. 9, pp. 935-937, 2012, doi: 10.1176/appi.ps.201100468.
36. L. Dixon et al., "Use of a Critical Time Intervention to Promote Continuity of Care After Psychiatric Inpatient Hospitalization," Psychiatric Services, vol. 60, no. 4, pp. 451-458, 2009/04/01 2009, doi: 10.1176/ps.2009.60.4.451.
37. C. Clark, C. C. Guenther, and J. N. Mitchell, "Case management models in permanent supported housing programs for people with complex behavioral issues who are homeless," Journal of Dual Diagnosis, vol. 12, no. 2, pp. 185-192, 2016.
38. K. Jones et al., "Cost-effectiveness of critical time intervention to reduce homelessness among persons with mental illness," Psychiatric Services, vol. 54, no. 6, pp. 884-890, 2003.
39. S. Sheikh and D. Teeman, "A rapid evidence assessment of what works in homelessness services," ed: UK. Retrieved from www. pc. gov. au/rogs, 2018.
40. E. A.-O. X. Tweed et al., "Health of people experiencing co-occurring homelessness, imprisonment, substance use, sex work and/or severe mental illness in high-income countries: a systematic review and meta-analysis. LID - jech-2020-215975 [pii] LID -10.1136/jech-2020-215975 [doi]," (in eng), no. 1470-2738 (Electronic), 2021.
41. D. Herman, A. Conover S Fau - Felix, A. Felix A Fau - Nakagawa, D. Nakagawa A Fau - Mills, and D. Mills, "Critical Time Intervention: an empirically supported model for preventing homelessness in high risk groups," (in eng), no. 0278-095X (Print), 2007.
42. C. L. Caton, A. Wyatt Rj Fau - Felix, J. Felix A Fau - Grunberg, B. Grunberg J Fau - Dominguez, and B. Dominguez, "Follow-up of chronically homeless mentally ill men," (in eng), no. 0002-953X (Print), 1993.
43. D. Herman and J. Mandiberg, "Critical Time Intervention: Model Description and Implications for the Significance of Timing in Social Work Interventions," Research on Social Work Practice - RES SOCIAL WORK PRAC, vol. 20, 09/01 2010, doi: 10.1177/1049731509360667.
44. T. Aubry et al., "A randomized controlled trial of the effectiveness of Housing First in a small Canadian City," BMC Public Health, vol. 19, no. 1, p. 1154, 2019/08/22 2019, doi: 10.1186/s12889-019-7492-8.

For peer review only

45. G. F. Moore et al., "Process evaluation of complex interventions: Medical Research Council guidance," BMJ, vol. 350, 2015.
46. T. F. C. da Silva, G. M. Lovisi, and S. Conover, "Developing an instrument for assessing fidelity to the intervention in the Critical Time Intervention-Task Shifting (CTITS)—Preliminary report," Archives of Psychiatry and Psychotherapy, vol. 16, no. 1, pp. 55-62, 2014, doi: 10.12740/APP/23279.
47. S. M. Eldridge et al., "CONSORT 2010 statement: extension to randomised pilot and feasibility trials," (in eng), no. 1756-1833 (Electronic), 2016.
48. V. Braun and V. Clarke, "Using thematic analysis in psychology," Qualitative research in psychology, vol. 3, no. 2, pp. 77-101, 2006.
49. N. Kozloff, A. D. Pinto, V. Stergiopoulos, S. W. Hwang, P. O'Campo, and A. M. Bayoumi, "Convergent validity of the EQ-5D-3L in a randomized-controlled trial of the Housing First model," BMC health services research, vol. 19, no. 1, p. 482, 2019.
50. NICE, "Methods for the Development of NICE Public Health Guidance", ed. London: National Institute for Clinical Excellence, 2012.
51. R. Etzioni, M. Urban N Fau - Baker, and M. Baker, "Estimating the costs attributable to a disease with application to ovarian cancer," (in eng), no. 0895-4356 (Print), 1996.
52. N. Latimer et al., "HEDS Discussion Paper 11/10 - Modelling the cost effectiveness of a potential new neck collar for patients with motor neurone disease," University of Sheffield, 2011.
53. E. Fenwick et al., "Value of Information Analysis for Research Decisions—An Introduction: Report 1 of the ISPOR Value of Information Analysis Emerging Good Practices Task Force," Value in Health, vol. 23, no. 2, pp. 139-150, 2020/02/01/ 2020, doi: https://doi.org/10.1016/j.jval.2020.01.001.
54. H. Squires, J. Chilcott, R. Akehurst, J. Burr, and M. P. Kelly, "A framework for developing the structure of public health economic models," Value in Health, vol. 19, no. 5, pp. 588-601, 2016.
55. M. Farrell and J. Marsden, "Acute risk of drug-related death among newly released prisoners in England and Wales," Addiction, https://doi.org/10.1111/j.1360-0443.2007.02081.x vol. 103, no. 2, pp. 251-255, 2008/02/01 2008, doi: https://doi.org/10.1111/j.1360-0443.2007.02081.x.

For peer review only - http://bmjopen.bmj.com/site/about/guidelines.xhtml

26 Appendices

26.1 Logic model

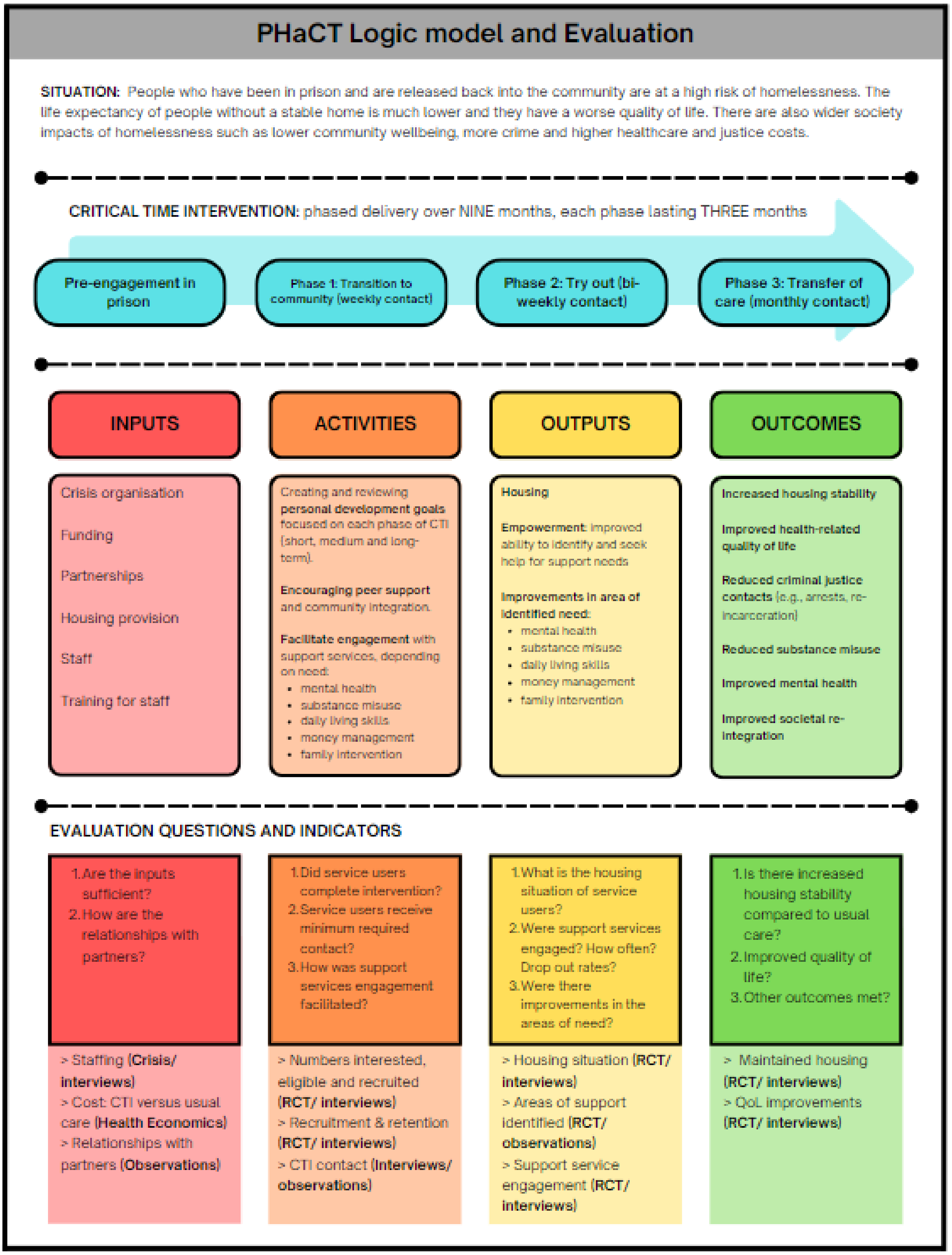

For peer review only - http://bmjopen.bmj.com/site/about/guidelines.xhtml
